# Cell type- and time-dependent biological responses in *ex vivo* perfused lung grafts

**DOI:** 10.3389/fimmu.2023.1142228

**Published:** 2023-07-03

**Authors:** Carla Gouin, Thien-Phong Vu Manh, Luc Jouneau, Claudia Bevilacqua, Julien De Wolf, Matthieu Glorion, Laurent Hannouche, Céline Urien, Jérôme Estephan, Antoine Roux, Antoine Magnan, Morgan Le Guen, Bruno Da Costa, Christophe Chevalier, Delphyne Descamps, Isabelle Schwartz-Cornil, Marc Dalod, Edouard Sage

**Affiliations:** ^1^ Université Paris-Saclay, INRAE, UVSQ, VIM, 78350, Jouy-en-Josas, France; ^2^ Aix-Marseille University, CNRS, INSERM, CIML, Centre d'Immunologie de Marseille-Luminy, Turing Center for Living Systems, Marseille, France; ^3^ Université Paris-Saclay, INRAE, UVSQ, BREED, 78350, Jouy-en-Josas, France; ^4^ Université Paris-Saclay, INRAE, AgroParisTech, GABI, 78350, Jouy-en-Josas, France; ^5^ Department of Thoracic Surgery and Lung Transplantation, Foch Hospital, Suresnes, France; ^6^ Department of Pulmonology, Foch Hospital, Suresnes, France; ^7^ Department of Anesthesiology, Foch Hospital, Suresnes, France

**Keywords:** transplantation, lung, single cell RNA-seq, monocyte/macrophages, inflammation

## Abstract

In response to the increasing demand for lung transplantation, *ex vivo* lung perfusion (EVLP) has extended the number of suitable donor lungs by rehabilitating marginal organs. However despite an expanding use in clinical practice, the responses of the different lung cell types to EVLP are not known. In order to advance our mechanistic understanding and establish a refine tool for improvement of EVLP, we conducted a pioneer study involving single cell RNA-seq on human lungs declined for transplantation. Functional enrichment analyses were performed upon integration of data sets generated at 4 h (clinical duration) and 10 h (prolonged duration) from two human lungs processed to EVLP. Pathways related to inflammation were predicted activated in epithelial and blood endothelial cells, in monocyte-derived macrophages and temporally at 4 h in alveolar macrophages. Pathways related to cytoskeleton signaling/organization were predicted reduced in most cell types mainly at 10 h. We identified a division of labor between cell types for the selected expression of cytokine and chemokine genes that varied according to time. Immune cells including CD4^+^ and CD8^+^ T cells, NK cells, mast cells and conventional dendritic cells displayed gene expression patterns indicating blunted activation, already at 4 h in several instances and further more at 10 h. Therefore despite inducing inflammatory responses, EVLP appears to dampen the activation of major lung immune cell types, what may be beneficial to the outcome of transplantation. Our results also support that therapeutics approaches aiming at reducing inflammation upon EVLP should target both the alveolar and vascular compartments.

## Introduction

In order to find a solution to donor lung shortage, a technique called *ex vivo* lung perfusion (EVLP) has emerged in the 2000s, in which isolated lungs are perfused and ventilated at normothermia for several hours prior to lung transplantation (LT) (1). This technique permits the safe preservation of organs between procurement and transplantation and upon EVLP, lungs falling outside organ acceptance standards recover the functional criteria requested for LT, making EVLP an important organ reassessment technique (2). In clinical practice, EVLP is conducted during 4-6 h. When that duration is prolonged, oedema occurs systematically and stands as a major issue of EVLP management (3). Yet prolongation is desired to optimize operating room logistics (4), monitor advanced biomarkers of LT prognosis (5), and apply specific targeted treatments with production of effects such as tissue repair (6), or immunomodulation (7). Hence the mechanisms leading to oedema need to be understood in order to control that issue. EVLP is also associated to inflammatory and stress responses (8, 9) whose extent correlates with primary graft dysfunction (PGD) after lung transplantation in patients (5). This inflammatory milieu that includes inflammatory cytokines (9), reactive oxygen species (8), and damage-associated molecular pattern molecules (10) might trigger the priming of lung donor myeloid antigen-presenting cells such as dendritic cells and lymphoid cells for the induction of adverse immune responses leading to PGD and rejection events.

In order to develop treatments for further improving the results of EVLP, we need to fill the gap of knowledge regarding the biological response induced by EVLP. Indeed the cell-signaling related to the extracorporeal perfusion with synthetic liquid ± erythrocytes and the non-physiological positive pressure of ventilation may differentially affect epithelial, vascular, and immune cell compartments. Several reports have provided transcriptomic information on the global organ response (11–13), but so far not at the cell type level. The single-cell RNA-sequencing (scRNA-seq) technique allows to simultaneously evaluate the gene response of multiple cell types in a tissue. Therefore its implementation for EVLP would both advance our knowledge on the biological effects of EVLP and provide an investigating platform to test therapeutic strategies aiming at reducing inflammation and potentially reconditioning initially damaged organs. In this study, we generated scRNA-seq datasets using two human lungs undergoing prolonged EVLP up to 10 h. Despite the scarcity of donor lungs, these lungs, although well-functional, were declined for transplantation due to lack of recipient of compatible ABO blood group or due to age, therefore such biological material is rare for its availability to research. We robustly identified the different lung cell types and revealed the enriched functional pathways common to the two different donors. Inflammatory pathways were predicted to be upregulated in blood endothelial and epithelial cells, monocyte-derived macrophages (MoMacs) and only transiently in alveolar macrophages (AMs), whereas pathways related to cell activation were absent or reduced upon EVLP in several immune cell types, i.e. T cells, NK cells, mast cells and conventional type 2 dendritic cells (cDC2s). Pathways related to cytoskeleton signaling/organization showed reduction in most cell types, especially at 10 h. Together with providing new insight in the biological response to *ex vivo* perfusion, this study also shows that scRNA-seq applied to EVLP will be a highly useful tool for assessing the effects of therapeutic regimens that may further improve the reconditioning of lungs through targeting the alveolar and vascular compartments.

## Results

### Identification of lung cell types from scRNA-seq data that display important transcriptomic changes upon EVLP at 4 h and 10 h

**Figure 1:**
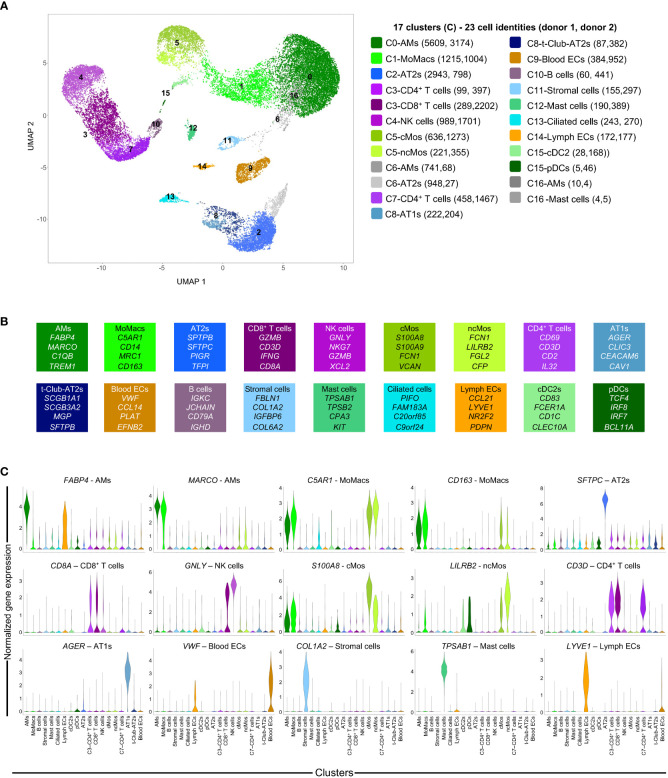
Single cell RNA-seq analysis of lung samples at 3 EVLP timing (0, 4, 10 h) and definition of cell identities. **(A)** The scRNA-seq data of the 6 samples (2 donors, 3 timings) were processed for high quality transcriptomes, integrated, clustered and submitted to cell annotation analysis with the Azimuth package (see **Additional file 2** and Methods). Cells with low annotation scores (< 0.6) and minor identities (<10% of a cluster) were excluded and the remaining cells were projected onto the “integrated UMAP-filtered” shown in A, with 17 clusters (C0 to C16, shown on the UMAP). Cell identities (23 in total) were determined by i) the belonging to a cluster of the “integrated UMAP-filtered” (Cx number), ii) the Azimuth-based annotation and iii) a grouping of “close cell subtypes” into a generic cell type annotation (see **Additional files 2**, **6**). The 23 cell identities are each associated to a distinct color related to a dominant tint corresponding to major cell type family (green for myeloid, pink for lymphoid, blue for epithelial and stromal, gold for vascular cells); these colors will be kept in the subsequent figures. The scRNA-seq data are available on an interactive viewer (https://applisweb.vim.inrae.fr/Human_EVLP). The number of cells originating from donor 1 and donor 2 in each cell identity is reported between brackets. Ambiguous identities were excluded (C6-AMs, C6-AT2s, C16-AMs, C16-Mast cells)) leading to 19 cell identities used for subsequent analyses. **(B)** Hallmark genes supporting the identification of the different cell identities were selected from the top expressed genes common to both donors (**Additional file 7**). **(C)** Violin plots showing the original level of expression of selected hallmark genes in the 19 cell identities of donor 2 (similar in donor 1).

In order to implement a scRNA-seq workflow for analyzing human lungs undergoing acellular EVLP, we collected lung tissue samples from two donor lungs declined for transplantation. Donor 1’s lung was declined due to absence of suitable recipient of the ABO blood group, and donor 2’s lung was declined due to age. These lungs did not show initial macroscopic inflammatory lesions nor any signs of injury, their respiratory functions were both very good during the whole duration of EVLP (> 400 mmHg) and of a “transplantable” quality on that basis, as shown in **Additional file 1**. The samples were collected at 0 h (just before EVLP initiation, i.e. “untreated”), and after 4 h and 10 h EVLP from the two donors, with care to take similar upper lung zones. The scRNA-seq (6 in total) were conducted on 2x10^4^ loaded cells from each sample using 10X Genomics 3’end RNA-seq V3 chemistry. The work flow of the analysis of the scRNA-seq data sets is presented in **Additional file 2** and extensively detailed in the Methods. High-quality transcriptomes from 43,569 cells were generated upon removal of mitochondrial genes and cell doublets (**Additional files 3**, **4**). The clustering of the scRNA-seq data of each donor in “time-combined UMAPs” showed that EVLP induced major transcriptomic changes (**Additional file 5**). In order to identify clusters corresponding to the same cell types across donors and timings, we integrated our six samples using a batch correction algorithm, generating an initial “integrated UMAP” with 17 clusters that are populated by cells from both donors at all timings (**Additional file 6**). In order to determine the cell identity, we applied Azimuth to map our scRNA-seq data onto the Human Lung Cell Atlas (Human lung reference v2). Upon filtration of the data based on Azimuth scores and exclusion of minor identities (see **Additional files 2**, **6**), we generated an “integrated UMAP-filtered” (**Figure 1A**) encompassing 23 cell identities based on cluster (Cx) belonging and Azimuth annotation, i.e. C0-alveolar macrophages (AMs), C1-monocyte-derived macrophage (MoMacs), C2-alveolar type 2 (AT2s), C3-CD4^+^ T cells, C3-CD8^+^ T cells (CD8^+^ T cells), C4-NK cells (NK cells), C5-classical monocytes (cMos), C5-non-classical monocytes (ncMos), C6-AMs, C6-AT2s, C7-CD4^+^ T cells, C8-AT1s (AT1s), C8-transitional-Club-AT2 (t-Club-AT2s), C9-blood endothelial cells (blood ECs), C10-B cells (B cells), C11-stromal cells (stromal cells), C12-mast cells (mast cells), C13-multi-ciliated epithelial cells (ciliated cells), C14-lymphatic endothelial cells (lymph ECs), CD15-conventional type 2 dendritic cells (cDC2s), CD15-plasmacytoid dendritic cells (pDCs), C16-AMs and C16-mast cells. In the next analyses, we excluded C6-AMs/C6-AT2s and C16-AMs/C16-mast cells, due to their ambiguity; indeed despite the use of Scrublet to remove doublets in the data processing (see **Additional file 2** and Methods), these clusters included distant subtypes yet clustering together, and C16 included very few cells (<15 per donor). **Figure 1A** shows that the remaining 19 cell identities were represented in both donor data sets (see numbers in brackets), indicating that our experimental setting is not biased to a donor. In addition as shown in **Figures 1B, C**, the cell type identity was confirmed by the expression of recognized hallmark genes (14, 15) that were found among the top expressed markers of our 19 cell identities (**Additional files 7**, **8** for gene symbols), such as *FABP4* and *MARCO* in AMs, *C5AR1 and CD163* in MoMacs, *S100A8* in cMos, *LILRB2* in ncMos, *CD1c* in cDC2s, *TPSAB1* in mast cells, *AGER* in AT1s*, SFTPC* in AT2s, *VWF* in blood ECs, *LYVE1* in lymph ECs, *COL1A2* in stromal cells, *GNLY* in NK cells, *CD3D* in CD8^+^ T cells and CD4^+^ T cells, and *CD8A* in CD8^+^ T cells as well as in C3-CD4^+^ T cells but not in C7-CD4^+^ T cells (**Additional files 7**, **8**). Compared to C7-CD4+ T cells, C3-CD4^+^ T cells share more gene expression with CD8^+^ T cells as they cluster together, and expression of *CD8A* is known to be expressed by some activated CD4^+^ T cells (16). We calculated the percentage of representation of the cell identities for each donor during EVLP (**Additional file 9**). We found that a similar representation of the 19 identities was obtained over time, indicating that the cellular composition of the lung samples was not substantially modified by EVLP.

In the rest of our study, we grouped the analyses by cell type families i.e. epithelial (AT1s, AT2s, t-Club-AT2s, ciliated), endothelial (blood and lymph ECs), myeloid (AMs, MoMacs, cMos, ncMos, mast cells, cDC2s, pDCs), and lymphoid cell type families (CD4^+^ and CD8^+^ T cells, NK cells, B cells). The gene expression across cell type identities and clusters can be visualized with an interactive viewer (https://applisweb.vim.inrae.fr/Human_EVLP).

For each of the 19 cell identities obtained by our strategy based on Azimuth scoring and exclusion of minor identities and ambiguous clusters, we extracted differentially expressed genes (DEGs, adjusted p-value < 0.05, absolute fold change > 0.4 (Log2), **Additional file 10**) for each donor between 0 versus 4 h and 0 versus 10 h, for downstream functional enrichment analyses. DEGs were processed through Ingenuity Pathways Analysis (IPA), which predicts modulation of functions and pathways by integrating the orientation of gene expression modulation and a scientific literature-based curated database. We selected the meaningful pathways and functions that were predicted to be modulated similarly in both donors at least at one time point, and we systematically added the IPA functions designated as Inflammatory Responses, Inflammation of Lung, Apoptosis and Cell Viability. No consistent pathways/functions were found for stromal cells, t-Club-AT2s, ciliated cells, B-cells and pDCs, that were thus not considered for further analyses. In order to confirm and possibly complete the IPA analysis, we performed a high-throughput Gene Set Enrichment Analysis (GSEA) using BubbleMap applied to the Hallmark collection from the Molecular Signatures Database (MsigDB). GSEA performs statistical enrichment analyses using genesets mapped onto the gene expression comparison between two timings, and hence is not restricted to the DEGs; in addition, IPA and GSEA use different knowledge-based annotations for a given function/pathway, leading to often similar but sometimes different results between the two approaches (see Discussion). The functional predictions inferred from the gene expression modulations will be detailed next for the different cell type families.

### Lung epithelial cells during EVLP present gene pathways related to increased inflammation and decreased cytoskeleton signaling

**Figure 2:**
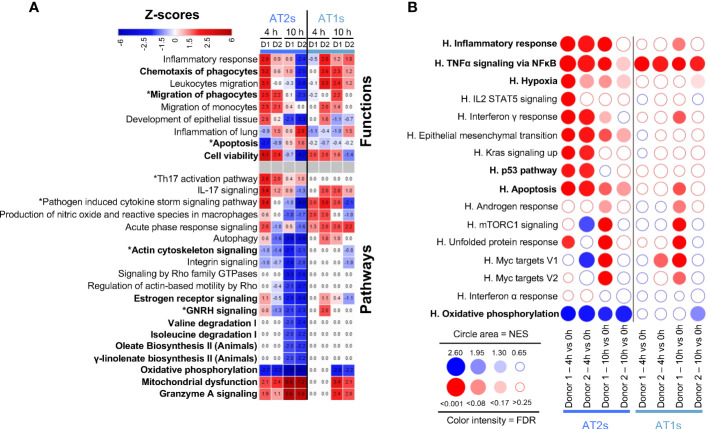
Computational inference of the changes induced by EVLP in lung epithelial cells. **(A)** The differentially expressed genes (DEGs, adjusted p-values p < 0.05, Log2FC > 0.4) in alveolar type 2 (AT2s) and alveolar type 1 (AT1s) between 4 h vs 0 h and 10 h vs 0 h were submitted to Ingenuity Pathways Analysis. The pathways and functions that showed z-scores < -1.9 or > 1.9 consistently in both donors (D1, D2) at 4 h or 10 h are presented as a heatmap, with stop colors indicated on the scale. The z-score values are reported for each condition (donor, timing), with values < -2 or > 2 being considered by IPA as significantly predictive of inhibition or activation respectively. Functions/pathways in bold fonts are mentioned in the main text. The modulated expressions of the contributing genes to the functions/pathways marked with a star (*) are provided in **Additional file 11**. **(B)** Bubblemap showing the Gene Set Enrichment Analysis performed on comparisons between 4 h vs 0 h and 10 h vs 0 h for AT2s and AT1s using the Hallmark (H) gene sets of the MSigDB. The leading edge genes that contribute the most to the enrichments are provided in **Additional file 12**. The genesets enriched at 4 h or 10 h vs 0 h are represented as red bubbles when positively enriched and as blue bubbles when negatively enriched. The circle area is proportional to the Normalized Enrichment Score (NES) and the color intensity corresponds to the False Discovery Rate (FDR, significance < 0.25).

As shown in **Figure 2A**, functions/pathways related to inflammation were predicted by IPA to be activated in AT1 and AT2 cells. In particular, Chemotaxis and Migration of phagocytes were predicted to be stimulated in several instances (z-scores > 2), with upregulation of *CXCL2*, *CXCL8*, *CCL2, CCL20* and *CSM1* gene expression (**Figure 2A**, **Additional file 11**). Viability was predicted activated at 4 h but tended to be reduced at 10 h in both cell types in link with stimulated Granzyme A Signaling and Mitochondrial Dysfunction, and reduction of Oxidative Phosphorylation, fatty acid and amino acid metabolisms and hormonal signaling. In parallel, Actin Cytoskeleton Signaling pathways were predicted downmodulated at 10 h, with reduced expression of actin family genes (*ACTB, ACTG1, ARPC1-5*), myosin genes (*MYO5C*, several *MYL)* as well as RHOA, a GTPAse that controls cytoskeleton organization (17) (**Figure 2A**, **Additional file 11**). GSEA confirmed an enrichment of Inflammatory Responses and TNFα Signaling via NFκB, in association with reduction of Oxidative Phosphorylation as found in many inflammatory situations (18) (**Figure 2B**). Upregulation during EVLP of the Hypoxia geneset in AT2s implicated increased expression of oxidative stress genes, such as metallothionein *(MT)1E*, *MT2A* (**Additional file 12** for the leading edge genes of GSEA). Furthermore, the p53 pathway was upregulated at 4 h in AT2s, with implication of *GADD45A* whose transcript levels are increased following stressful growth arrest conditions. Regarding Apoptosis, IPA predicted significant downmodulation of the function at 4 h in the case of AT2 cells of donor 1 (**Figure 2A**) and GSEA pointed to a significant positive enrichment (**Figure 2B**) Explanations for this discrepancy will be discussed next (see Discussion). Therefore, no clear conclusion can be drawn regarding prediction of Apoptosis in AT2s.

Altogether our scRNA-seq data analysis indicates that epithelial cells upon EVLP engaged gene pathways related to increased inflammatory responses, and particularly at 10 h, gene pathways related to decreased cytoskeleton signaling and decreased general metabolism.

### Blood endothelial cells show upregulation of gene pathways related to inflammation

**Figure 3:**
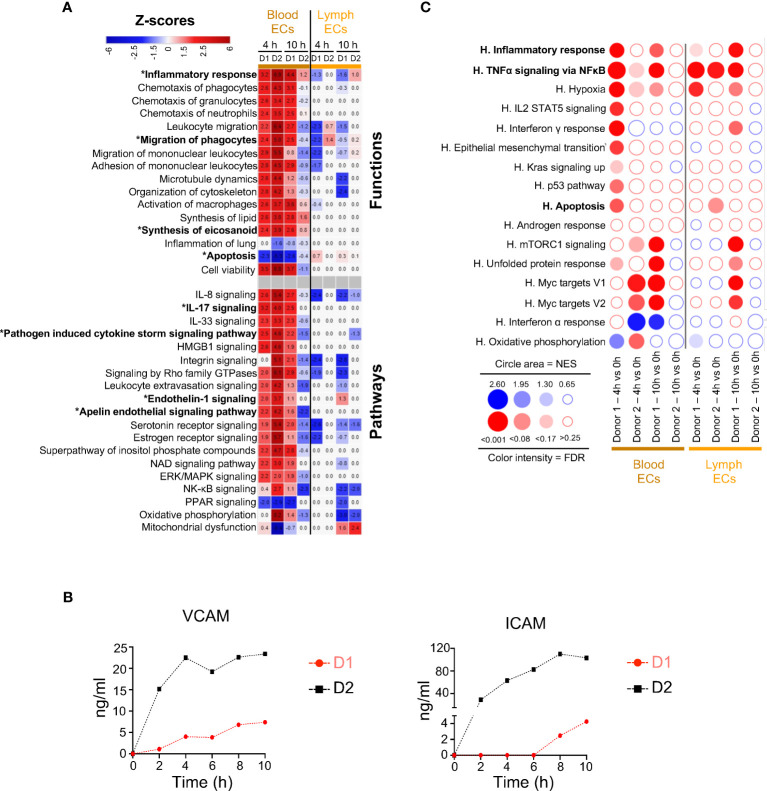
Computational inference of the changes induced by EVLP in endothelial cells (ECs). **(A)** The differentially expressed genes (DEGs, adjusted p-values p < 0.05, Log2FC > 0.4) in the blood and lymph endothelial cells (ECs) between 4 h and 10 h vs 0 h were submitted to Ingenuity Pathways Analysis. The predicted pathways and functions are illustrated as in **Figure 2A**. Functions/pathways in bold fonts are mentioned in the main text. The modulated expressions of the contributing genes to the functions/pathways marked with a star (*) are provided in **Additional file 13**. **(B)** Dosage of soluble ICAM and VCAM in the perfusion liquids of donor 1 and 2 by ELISA. **(C)** Bubblemap showing the Gene Set Enrichment Analysis performed on comparisons between 4 h vs 0 h and 10 h vs 0 h for the endothelial cells, like done in **Figure 2B**. The leading edge genes that contribute the most to the enrichments are provided in **Additional file 12**.

We next examined the functional gene modules altered by EVLP in the endothelial cell family, i.e. blood ECs and lymph ECs (**Figure 3**). In blood ECs at 4 h, IPA predicted activation of Inflammatory Response (z-scores > 3, reaching > 6 in several instances), such as Migration of phagocytes, Synthesis of eicosanoid, IL-17, and Pathogen Induced Cytokine Storm pathways (**Figure 3A**). The contributing genes to these pathways included many chemokine genes (*CXCL1, CXCL2, CXCL3, CXCL8, CCL2, CCL14*), cytokine (*IL6, MIF*) and cytokine receptor (*IL1R1*) genes as well as eicosanoid synthesis genes (*PTGS2*) and coagulation-implicated genes (*PLAT, PLAUR, SERPINE1*), that were upregulated in both donors at 4 h as well as at 10 h in donor 1 and less so in donor 2 (**Additional file 13**). Genes associated to endothelial cell activation (19), such as ICAM1 and VCAM1 were upregulated and their protein shedding also increased with time in the perfusion liquid (**Figure 3B**). The Endothelin-1 and Signaling Apelin pathways, that are implicated in the control of vascular permeability (20), were also predicted to be modulated (**Figure 3A**). The GSEA analysis (**Figure 3C**) confirms the enrichment of TNFα Signaling via NFκB and Inflammatory Responses in blood ECs and also in lymph ECs, although no clear prediction could be inferred from IPA in the latter cell type. As in the case of epithelial cells, no consistent conclusion from IPA and GSEA could be deduced regarding Apoptosis.

These data indicate that the gene expression profiles of blood ECs upon EVLP are associated with activation of inflammatory response and of Endothelin/Apelin pathways.

### Lymphoid cells display gene pathways related to decreased activation, cytoskeleton signaling and migration

**Figure 4:**
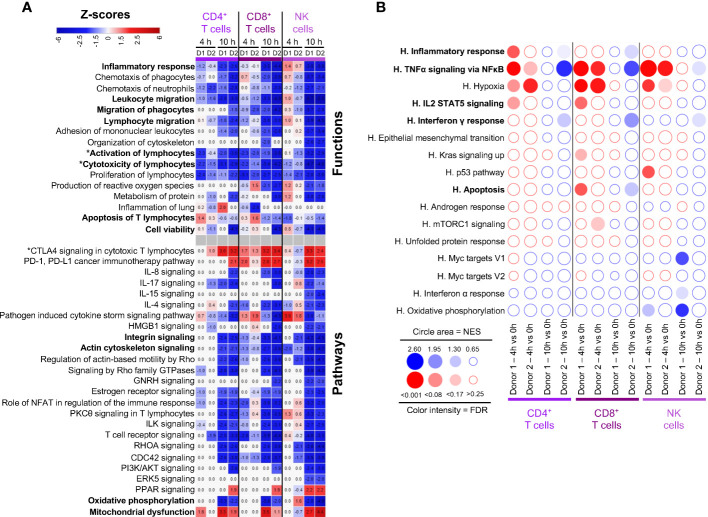
Computational inference of the changes induced by EVLP in lymphoid cells. **(A)** The differentially expressed genes (DEGs, adjusted p-values p < 0.05, Log2FC > 0.4) in the C7-CD4^+^ T cells, CD8^+^ T cells and NK cells between 4 h and 10 h vs 0 h were submitted to Ingenuity Pathways Analysis. The predicted pathways and functions are illustrated as in **Figure 2A**. Functions/pathways in bold fonts are mentioned in the main text. The modulated expressions of the contributing genes to the functions/pathways marked with a star (*) are provided in **Additional file 14**. **(B)** Bubblemap showing the Gene Set Enrichment Analysis performed on comparisons between 4 h vs 0 h and 10 h vs 0 h for the lymphoid cells, like done in **Figure 2B**. The leading edge genes that contribute the most to the enrichments are provided in **Additional file 12**.

Gene expression changes in lymphoid cells were analyzed by IPA, i.e. in C3-CD4^+^ T cells and C7-CD4^+^ T cells, CD8^+^ T cells, and NK cells. Similar IPA profiles were obtained with C3- and C7-CD4^+^ T cells, therefore only C7-CD4^+^ T cells (the dominant population) are shown in **Figure 4A**. IPA does not show prediction of activation of the Inflammatory Response pathway (z-score all < 2), in most cases in accordance with the GSEA analysis (**Figure 4B**). However GSEA points to enrichment of TNFα Signaling Pathway at 4 h in both donors; in that case, many of the leading edge genes were not statistically found as DEGs and thus not significantly/substantially upregulated (*IL18, IL23A, IL1A, NFKBIE, CCL2, SOD2, CXCL3, IL1B, NFKB2, RELB, VEGFA, MAFF, CXCL2, CCL20*, **Additional files 10**, **12**). Inhibition of Activation and Cytotoxicity of lymphocytes was predicted with IPA, already at 4 h in several instances, and in all cases at 10 h, reaching low negative z-scores (< -4, **Figure 4A**). Indeed the expression of several cytokine genes was reduced, i.e. *CCL3, CCL4, CCL5, IFNG*, as well as that of genes encoding key effector molecules (*GZMA*) and immune receptors (*CD3E, CD3G, TRBC1* and *2*, **Additional file 14**). Accordingly no significant enrichment of IL2-Stat5 Signaling and of Interferon gamma response was consistently found with GSEA at 4 h and both pathways tended to be downregulated at 10 h (**Figure 4B**). Importantly, the expression of genes involved in active suppressive functions were upregulated at 4 h in CD8^+^ T cells, such as *TNFAIP3*, a de-ubiquitinase gene that inhibits functional activation of immune cells mediated through NF-κB/STAT pathways (21), *SOCS1*, a negative regulator of cytokine receptors (22), and in some cases *CTLA4*, an immune checkpoint inhibitor (**Additional files 10**, **14**). Actin Cytoskeleton Signaling and Integrin Signaling pathways were predicted by IPA to be reduced particularly at 10 h, in link with decreased migration properties (**Figure 4A**). The Cell Viability function of IPA showed negative z-scores particularly at 10 h, in link with predicted Mitochondrial dysfunction and reduced Oxidative Phosphorylation (**Figure 4A**). Yet no significant prediction for activation of apoptosis-related pathways could be retrieved from IPA nor from GSEA.

Altogether, the gene signatures of lymphoid cells appear mainly related to blunted activation during EVLP, with upregulation of suppressive immune genes and reduction of cytoskeleton signaling and cell mobility pathways.

### Monocytic subsets and AMs show upregulation of gene pathways related to inflammatory processes whereas mast cells and cDC2s display predicted blunted activation

**Figure 5:**
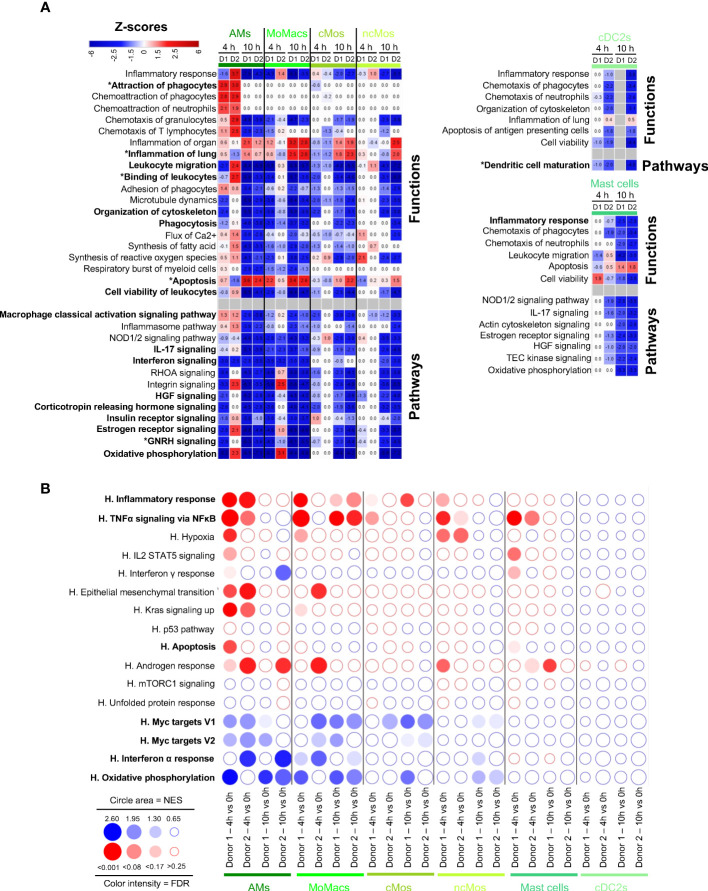
Computational inference of the changes induced by EVLP in myeloid cells. **(A)** The differentially expressed genes (DEGs, adjusted p-values p < 0.05, Log2FC > 0.4) in alveolar macrophages (AMs), monocyte-derived-macrophages (MoMacs), classical monocytes (cMos), non-classical monocytes (ncMos), mast cells and conventional type 2 dendritic cells (cDC2s) between 4 h and 10 h vs 0 h were submitted to Ingenuity Pathways Analysis. The predicted modulated pathways and functions are illustrated as in **Figure 2A**. Functions/pathways in bold fonts are mentioned in the main text. The modulated expressions of the contributing genes to the functions/pathways marked with a star (*) are provided in **Additional file 15**. **(B)** Bubblemap showing the Gene Set Enrichment Analysis performed on comparisons between 4 h vs 0 h and 10 h vs 0 h of myeloid cell types, like done in **Figure 2B**. The leading edge genes that contribute the most to the enrichments are provided in **Additional file 12**.

We finally conducted functional enrichment analysis on the six myeloid subtypes, i.e. AMs, MoMacs, cMos, ncMos, mast cells, cDC2s. Through IPA, AMs presented a predicted activated function of Attraction of Phagocytes at 4 h, implicating the upregulation of *CCL20, CXCL5, IL6* and *IL1B* genes in both donors at 4h that was dampened at 10 h (**Figure 5A**, **Additional file 15**). Consistently, GSEA showed a significant enrichment of the Inflammatory Response and TNFα Signaling via NFkB at 4 h and not at 10 h (**Figure 5B**). Through IPA, MoMacs and cMos displayed a predicted participation to Inflammation of Lung merely at 10 h, with upregulated expression of IL-1β related genes (*IL1B, IL1R1, IL1RL*1), eicosanoid synthesis gene (*PTGS2*) and *CXCL3* gene (**Figure 5A**, **Additional file 15**). GSEA also showed enrichment of the inflammatory pathways at 10 h in MoMacs and a tendency in Mos (**Figure 5B**). IPA predicted in most instances the reduction of Macrophage Classical Activation Signaling Pathways, IL-17 and Interferon Signaling, Organization of cytoskeleton, Phagocytosis, Binding of Leukocytes and Migration, hormone-driven pathways, often already at 4 h and in almost all cases at 10 h. In particular the expression of integrin genes was downmodulated (*ITGB1, ITGB2, ITGA4*), as well as that of vimentin (*VIM*), and tetraspanin (*CD37*) (**Additional file 15**). The prediction of engagement of gene pathways related to Apoptosis was again not consistent between IPA and GSEA (see discussion). Particularly at 10 h, IPA predicted reduction of Viability and reduction of Oxidative Phosphorylation as confirmed by GSEA that also pointed to reduction of MYC target and Interferon Alpha Response pathways. Mast cells and ncMos did not present a clearly predicted participation to inflammatory functions and their functions were essentially predicted to be inhibited.

Despite that DEGs could not be retrieved in the case of the cDC2s of donor 1 at 10 h due to too few cells, we decided to investigate the functional genomic results of this major cell subtype involved in orchestration of immunity. Reduction of cell activation and maturation appeared to apply to cDC2s, at 4 h and 10 h in donor 2, and following the same trend at 4 h in donor 1. Notably, *CD83*, and several genes encoding for MHC class 2 proteins (*HLA-DR, -DQ* and *-DP*), *CD1D, IL1B* and *IL18* were downmodulated. Similarly GSEA confirmed that cDC2 tended to downregulation of most pathways upon EVLP.

Collectively our data indicate that myeloid cells differentially responded to EVLP: AMs transiently upregulated inflammatory pathways at 4 h only, MoMacs and cMos upregulated inflammatory gene pathways particularly at 10h, and ncMo, mast cells and cDC2 essentially showed dampened activation upon EVLP. In all our analyzed myeloid subsets, organization of cytoskeleton and migration were predicted to be reduced particularly at 10 h.

### Relative contribution of cell types to the cytokine and chemokine transcript synthesis during EVLP

**Figure 6:**
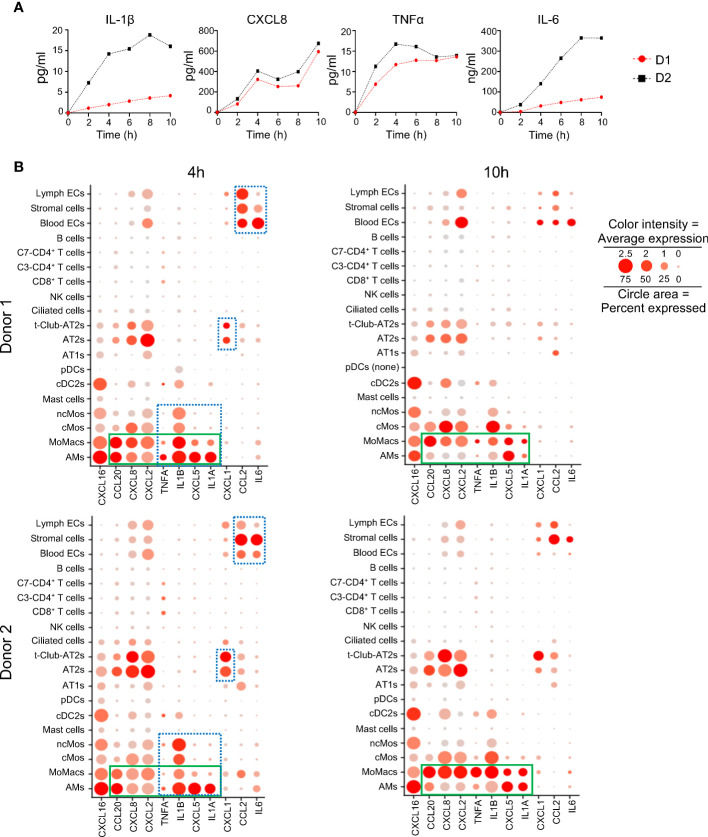
Changes induced by EVLP in cytokine and chemokine protein or mRNA expression. **(A)** Cytokine detection in perfusion fluid. Perfusion fluid was collected every 2 h of the 10 h EVLP procedure. IL-1β, CXCL8, TNFα were detected with a Multiplex Luminex assay and IL-6 with ELISA. The values of donor 1 (D1) and donor 2 (D2) are in red black, respectively. **(B)** The normalized average gene expression of each cytokine/chemokine in the different clusters of the two donors is illustrated by the red color intensity in a dot plot. The proportion of cells expressing the reported genes in the cluster is proportional to the circle area. The cytokine/chemokine expression at 4 h that is specific to a cell type family (myeloid, epithelial, stromal/endothelial) is highlighted by a blue rectangle with dash lines. The cytokine/chemokine expression that switches to dominance in MoMacs versus AMs at 10 h is highlighted by a green rectangle. Note that in the samples of donor 1 at 10 h, pDCs were absent (pDC none).

The inflammatory response revealed to be a dominant function induced upon EVLP in several cell types. This activated function was reflected by the production of cytokines and chemokines in the perfusion liquid (IL-1β, CXCL8, TNFα, IL-6), that accumulated overtime despite perfusion fluid replacement during the process (**Figure 6A**). The absolute average expression values of cytokine and chemokine genes (**Figure 6B**, dotted blue squares) indicate selective expression per cell type families particularly at 4 h; for instance *TNFA, IL1B, CXCL5 and IL1A* were mainly expressed by monocyte/macrophages, CXCL1 by epithelial cells, *CCL2* and *IL6* by vascular cells and stromal cells. *CXCL2*, *CXCL8, CXCL16 and CCL2 genes* were expressed by most cell types except by lymphoid cells. Notably the expression of *CCL20, CXCL8, CXCL2, TNFA, IL1B, CXCL5 and IL1A* show a dominant expression in MoMacs as compared to AMs at 10 h (**Figure 6B**, green square).

These results show that lung cell types display a division of labor for the selective expression of cytokine/chemokine genes that varies with time during EVLP.

## Discussion

By performing a thorough scRNA-seq analysis focusing on the common responses of two lung donors undergoing EVLP, we showed that EVLP induces changes in the expression of genes involved in inflammation and cytoskeleton signaling/organization, depending on cell types and timing. Inflammatory functions were predicted to be induced in epithelial cells, blood endothelial cells, MoMacs and transiently in AM at 4 h. Globally, NK cells, T-cells, mast cells, ncMos and cDC2s displayed absent or reduced activation. Reduction of cytoskeleton signaling/organization appeared in most cell types mainly at 10 h associated with a prediction for reduced migration properties in lymphoid and myeloid cells. This latter result may explain the reduction of passenger leukocyte transfer encountered after transplantation of lungs treated with EVLP (23). As rapid transfer of passenger leukocytes is known to promote acute cellular rejection (23), this reduction of migration, together with the global downmodulation of activation in cDC2s and lymphoid cells, might stand as a positive effect of EVLP. Indeed several papers suggest that LT with EVLP leads to better clinical outcomes, although this statement needs to be confirmed with additional clinical studies (24–26).

In most instances, the IPA and GSEA approaches gave consistent results except in the case of Apoptosis, a form of non-inflammatory and controlled cell death. The discrepancy can be explained by i) the differences (in terms of numbers and identities) of the Apoptosis genes found in the IPA-knowledge database (4597 genes) and in MSigDB (161 genes) and ii) the difference of methodologies that involves DEGs in IPA and ranked gene expression in GSEA (see **Additional file 16**). For example, in monocytes/macrophages at 10 h EVLP, most IPA Apoptosis-contributing genes are not taken into account in the GSEA analysis because only a minority of the IPA genes (8 to 12%) is present in the Hallmark Apoptosis gene set (**Additional file 16**). Reciprocally in AT2s and blood ECs at 4 h EVLP, a majority of the genes found in the GSEA Apoptosis leading edges with positive enrichment (about 60%) are not DEGs and thus not taken into account in the IPA analysis. Finally IPA results are expected to be more robust because, at the difference with the Hallmark Apoptosis gene set that includes both pro-apoptosis (*BAX, CASP1 to 9*) and anti-apoptosis (*BCL2L1, BCL2L10, BCL10*) genes, IPA takes into account the pro- and anti-apoptotic functions and their orientation of expression in our data set. In any event, complementary analyses should be conducted in additional studies to conclude about the possible activation of apoptosis in specific cell subsets upon EVLP according to its duration. Notably based on IPA, Cell Viability appeared activated at 4 h in AT1s, AT2s and blood ECs, but reduced at 10 h in most cell types (**Figures 2**, **3**).

EVLP is associated to an ischemia-reperfusion-like response, inherent to lung retrieval and preservation followed by a reperfusion process at normothermia (27). Secondly the intensive care unit ventilators currently used in EVLP are proposed to induce positive pressure ventilation-induced lung injury which results from an interplay of cell responses to supra-physiological mechanical forces (28). Both phenomena may be involved in inducing the EVLP inflammatory response via the implication of alveolar and vascular cells as revealed by this study. We propose that the inflammatory response expressed by these cell types triggers the oedema that occurs upon prolonged EVLP. Indeed inflammation is known to drive perturbations of cytoskeleton signaling that was found in AT2s for instance, and alteration of cytoskeleton signaling is a master regulator mediating disruption of junctions and creation of leaky barriers (17). In addition the activation of the endothelin pathway that was inferred from gene expression modulation in blood ECs may also contribute to an increased blood vessel permeability and thus to oedema formation (20).

The magnitude of the inflammatory response during EVLP correlates with worse outcome after lung transplantation in patients (5, 29). Indeed CXCL8 and CXCL1 protein levels in the EVLP perfusate were previously shown to correlate with occurrence of PGD (5). In addition, an inflammation score based on CXCL8 and IL-6 expression in the perfusate during EVLP was also correlated with PGD (p=0.03) (29). The genes encoding for these cytokines were upregulated in the endothelial, epithelial and mononuclear phagocyte compartments in our study (**Additional file 17**), with major producers being epithelial cells for *CXCL1* and endothelial and stromal cells for *IL6* (**Figure 6B**). These cytokine responses could be modulated through treatments targeting the right cell compartments. First, the deleterious effects of positive pressure ventilation could be controlled through development of negative pressure ventilation, which was recently shown to reduce inflammatory responses in EVLP (30). We are currently evaluating the impact of positive versus negative pressure ventilation on EVLP using scRNA-seq, in order to provide mechanistic insight in the lung cell response to mechanical forces, and potentially identify specific pathways and specific cell targets. Secondly, the additional use of anti-inflammatory regimens, delivered in the perfusate and in the airways, could complement the methylprednisolone that is used as a standard of care and does not suffice to control the inflammatory response as shown here. Promising results were obtained with vectorized IL-10 (7), 1-anti-trypsin (31), Pyrrolidine dithiocarbamate (an inhibitor of NF-κB) (32) and mesenchymal stem cells (33). Interestingly our results also indicate that gene pathways related to hormonal signaling are decreased in several cell types (epithelial, myeloid, lymphoid families) particularly at 10 h. Therefore addition of hormones, the nature of which remains to be evaluated, might mitigate the response to EVLP. Importantly the scRNAseq-based workflow that we designed here with unbiased lung cell-type identification and functional genomics analyses constitutes a powerful approach to evaluate the effects of new treatments during EVLP that could be applied both on the airway and vascular compartments.

Our results point to the added value of scRNA-seq versus bulk RNA-seq approaches. Activation of gene expression related to inflammatory responses has been identified in EVLP with bulk approaches (12, 13). However, the bulk approach led to conclude that prolonged EVLP resolved the inflammatory response, with circulating leukocyte cell-specific gene expression dropping down, suggesting a washout of circulating leukocytes (12). In contrast, our scRNA-seq results show that the representation of the leukocyte clusters is stable over time and that expression of leukocyte integrins are reduced with EVLP duration (**Additional file 10**), supporting gene downmodulation rather than cell subset emigration.

A limitation of our study resides in the use of only two donors. This is because of (i) the difficulty to access to lungs in good physiological conditions, with a schedule compatible with availability of all the team members required to launch the EVLP and harvest the cells at the 3 time points chosen (0h, 4h and 10h), and (ii) cost of the experiments. However, we believe that this limitation has been overcome by the depth and rigor of our functional genomic study of the lung cell subset responses according to EVLP duration. Indeed, we implemented a specific data integration strategy to correct for batch effects and focused on the commonalities of the EVLP response, shared between the two donors. This enabled us to reveal the essence of the core cellular response to EVLP, while systematically illustrating the corresponding individual gene responses of the two donors. The predicted modulation of functions and pathways in the lung cell types sometimes presented different temporality and different levels of IPA z-scores between the two donors. For instance, in AT2s and blood ECs, the Inflammatory Response appeared less sustained in donor 2 than in donor 1 (**Figures 2**, **3**). We also limited our analysis to 3 time points; gene expression in some of the lung cell subsets may follow different changes at other timings. In addition, the conventional method of lung transplantation implies cold storage of the whole organ, that can also alter RNA expression as shown for kidney in a pig model for instance (34). Comparison between EVLP and cold storage of same durations would be interesting to perform in order to strengthen the conclusion that the gene modulations that we observed are indeed specific of EVLP. Finally, we can speculate that reduction of costs of scRNA-seq experiments in the future will allow performing robust correlation studies between modulation of gene expression in specific subsets and donor characteristics or transplantation outcomes.

Altogether our study shows that scRNA-seq is a fruitful approach to provide refined knowledge on the biological response of organs undergoing normothermic *ex vivo* perfusion, at the cell type level. This workflow could be followed to finely analyze the effects of treatments during EVLP, such as negative ventilation, anti-inflammatory and immunomodulatory regimens, on the response of key cell subsets. Exploiting the scRNA-seq approach will help to get the highest benefit of EVLP with the goal of improving lung transplantation results.

## Material and methods

### Study design and *ex vivo* lung perfusion procedure

This study was approved by the Agence de la Biomédecine and by the Ministère de l’éducation nationale, de l’Enseignement Supérieur et de la Recherche, Direction Générale de la Recherche et de l’Innovation under the number 2020-007, as well as by the ethic committee of the Foch Hospital (IRB00012437). The two donor lungs used in this study to generate 6 scRNA-seq samples were from donation after brain death and were determined to be unsuitable for transplantation (**Additional file 1**). The main reason for decline were absence of recipient (AB+ blood group) for donor 1 and age for donor 2. The respiratory functions of the two lungs during EVLP is reported on **Additional file 1**. Donor lung retrieval was carried out according to current clinical practice using Perfadex (Vitrolife, Göteborg, Sweden) flush preservation. After transportation at 4°C, lungs were processed to EVLP that was conducted according to the Toronto protocol for 10 h at the Foch Hospital (Suresnes, France) (35). The circuit was filled with 1.5 L of Steen solution supplemented with 1 g methylprednisolone, 1 g cefuroxime, and 15000 UI heparin. A flow rate at 40% of the theoretical cardiac output was applied at normothermia. Steen perfusate (200 ml) was replaced with fresh one every 2 h.

### Sampling

Perfusates were collected every 2 hours and frozen at -80°C for cytokine detection. Lung biopsies (2 g each) were taken from similar lung zones from the upper lobes (wedges) just before EVLP (0 h), after 4 and 10 h EVLP, cut in 4 pieces, placed in 10 ml HypoThermosol^®^ (STEMCELL Technologies, Vancouver, Canada) and kept on ice for about 24 h. This process was shown to maintain lung tissue stability up to 72 h for scRNA-seq (36).

### Detection of cytokines, VCAM and ICAM in perfusion liquid

Perfusates were thawed and briefly centrifuged. VCAM1 was measured by a Human VCAM-1 Picokine™ ELISA kit (Boster Biological Technology Co., Ltd., Pleasanton, CA), ICAM1 by the Human ICAM-1/CD54 allele-specific Quantikine ELISA kit (R&D Systems, Inc., Minneapolis, MN) and IL-6 by the LEGEND MAX™ Human IL-6 ELISA kit (BioLegend, San Diego, CA) according to each manufacturer’s instructions. IL-1β, CXCL8, CXCL10 and TNFα were assessed with a Human ProcartaPlex™ Mix&Match 4-plex (ThermoFischer Scientific, Waltham, MA) using a MagPix instrument (Luminex, Austin, TX) and the data were analyzed with the Bio-Plex Manager software (Bio-Rad, Hercules, CA).

### Single cell suspensions from lung biopsies

Lung tissue from biopsies kept in HypoThermosol was minced finely with scissors, placed in Multi Tissue Dissociation Kit 1 solution as recommended by the manufacturer (Miltenyi Biotec, Bergisch Gladbach, Germany), and incubated at 37°C for 45 min in gentle agitation. The minced preparation was crushed on nylon mesh (1 mm) and filtered through successive nylon filters (500 µm, 100 µm, 40 µm). The cell suspension was washed in PBS (470 g, 15 min), processed to erythrocyte lysis, resuspended in RPMI + 2% FCS, filtered twice on 40 µm and counted by 3 independent measurements with a counting Malassez chamber slide. Trypan blue staining indicated that over 90% cells were viable.

### scRNA-seq and analysis of sequencing data

Fresh single-cell suspensions (2 x 10^4^ cells) were loaded onto the 10x Chromium to produce sequencing libraries, which were processed according to methods provided by 10x Genomics (v3 Chemistry). Cell cDNA was sequenced using the Truseq Illumina Stranded protocol and the Illumina NextSeq 550 sequencing machine (> 3x10^8^ reads/sample). The reads were aligned with Cell Ranger v3.1.0 on the human genome using the GRCh38 assembly and the GTF file downloaded from Ensembl release 101. The 6 samples (3 times points, 2 donors) were pre-processed and normalized together using Seurat v4.3.0. Cells expressing less than 1000 genes were removed. Dead or lysed cells were excluded by removing cells with a percentage of mitochondrial genes above a threshold calculated using the Scater package (median percentage of mitochondrial genes across all individual cells + 3 median absolute deviations). Filtration of the data also included the removal of doublets using Scrublet (https://github.com/AllonKleinLab/scrublet (37), expected doublet rate set to 0.08), see **Additional files 3** and **4** for the details on the filtering procedure and the quality check features.

### Clustering strategies

The clustering process, annotation and downstream analyses are summarized in a workflow figure (**Additional file 2**). We first combined the scRNA-seq data of each donor (3 time points) and proceeded to a graph-based clustering resulting in an UMAP named “time-combined UMAP” (see **Additional file 5**) that included 18 clusters for each donor. In order to correct for the donor and time effect, we anchor-integrated the data of the 6 samples (2 donors and 3 time points) using the FindIntegrationAnchors and IntegrateData functions in Seurat and we produced the “integrated UMAP” (**Additional file 6A** **)**. Cells were preprocessed as described above. Parameters of the dimensionality reduction and graph-based clustering were adjusted to obtain 17 clusters in the “integrated UMAP” (**Additional files 3**, **6A**). Note that the anchor integration procedure was used only for integration of the 6 samples and assignment of cells to clusters. All downstream analyses use the expression values normalized for each donor separately, in order to avoid the data transformation linked to the anchor integration.

### Definition of cell identities in the scRNA-seq data sets

The original cell x gene matrix of each donor were pre-processed and normalized separately as described above and cells were assigned the cluster number of the “integrated UMAP”. Each donor dataset was then analyzed with Azimuth, an automated reference-based algorithm for single-cell annotation (https://azimuth.hubmapconsortium.org/, version 2.0.0), using the Human Lung Cell Atlas as a core consensus reference model which encompasses 584,884 human cells of the lung and nose. The finest level of annotation was used. Cells presenting Azimuth annotation scores below 0.6 were discarded. The remaining cells were hence associated to both a cluster number and a cell identity. In order to generate robust downstream analyses, the cell identities representing less than 10% of the associated cluster were eliminated for the subsequent analyses (**Additional file 6**). The cells selected with this Azimuth annotation-based process were projected onto the “integrated UMAP-filtered”, illustrated in **Figure 1A**. The final cell identities were based on i) their belonging to a cluster of the integrated UMAP, ii) their Azimuth-based annotation (> score 0.6) and iii) a grouping of “close cell subtypes” in the cases of the B lymphocytes (B cells, plasma cells), stromal cells (smooth muscle, adventitial and alveolar fibroblasts) and blood endothelial cells (arterial, aerocyte capillary, general capillary, venous systemic, venous pulmonary). Twenty three identities were thus obtained, from which we eliminated the C6 and C16 clusters, due to their ambiguity, leading to 19 final identities, i.e. C0-AMs, C1-MoMacs, C2-AT2s, C3-CD4^+^T, C3-CD8^+^T, C4-NK cells, C5-cMos, C5-ncMos, C7-CD4^+^ T cells, C8-AT1s, C8-transitional Club AT2, C9-blood ECs, C10-B cells, C11-stromal cells, C12-mast cells, C13-multi-ciliated, C14-lymphatic ECs, CD15-cDC2, CD15-pDC. The top markers of the 19 analyzed cell identities found in the “integrated UMAP-filtered” were extracted separately for each donor, using the normalized expression values of each dataset before integration [1000 top marker genes per cell identity versus the other identities (minimal log2FC>=0.8, Bonferoni adjusted p-value<0.05)]. The two lists were then merged into a single list to keep only common markers to both donors, ranked in a decreasing order using the lowest gene expression fold change between the two donors (**Additional file 7**).

### Generation of differentially expressed genes

Genes differentially expressed (absolute log2FC>0.4, Bonferroni adjusted p-value<0.05) between cells from the same identity at 0 h vs 4 h and 0 h vs 10 h were extracted using the FindMarkers function in Seurat applied on the data normalized separately for each donor **Additional file 10**. The fold change cut-off was established to comply to Ingenuity pathway analysis that recommends 200 to 3000 DEGs.

### Ingenuity pathway analysis

The DEG lists were submitted to IPA software on the IPA knowledge database restricted to immune cells, epithelial, endothelial and fibroblasts, with stringent filters, according to IPA Ingenuity Web Site, www.ingenuity.com. We selected the meaningful pathways and functions with z-scores > 1.9 or < -1.9) in the two donors at least at one time point for each cell type family. The z-score values are systematically reported on Figures. IPA considers that z-scores > 2 or < -2 correspond to function/pathways that are significantly predicted to be up- or down-modulated respectively. In all cases, apoptosis, cell viability, inflammation of lung and inflammatory response functions were additionally selected. For the most pertinent modulated functions or pathways, we illustrated the fold changes of the contributing genes (**Additional files 11**, **13**-**15**).

### GSEA results and BubbleGUM-based illustrations

To better characterize the gene expression changes that occurred upon EVLP using an alternative method to IPA, we applied high-throughput GSEA with the BubbleMap module of the BubbleGUM software (38) on the scRNA-seq data normalized separately for each donor, comparing 0 h vs 4 h and 0 h vs 10 h. We used the Hallmark (H) geneset collection from the Molecular Signatures Database [MSigDB v2023, https://www.gsea-msigdb.org/gsea/index.jsp (39)]. The retrieved annotations were selected based on two criteria: i) a false discovery rate (FDR) < 0.1 in GSEA and ii) a consistent and significant enrichment common to the two donors at least at one time point across all cell type identities. The leading edge genes that contribute the most to the enrichments are provided in **Additional file 12**. A correction for multiple testing across cell identities and conditions was applied in the frame of BubbleMap.

### Statistical analyses

The biostatistics tests used in this study are included in the DEG, IPA and GSEA sections.

## Data availability statement

The raw data obtained from the Cell Ranger analysis have been deposited on the Gene Expression Omnibus repository of the NCBI, under the accession number GSE218788. https://www.ncbi.nlm.nih.gov/geo/query/acc.cgi?acc=GSE218788.

## Ethics statement

The studies involving human participants were reviewed and approved by the Agence de la Biomédecine and by the Ministère de l’éducation nationale, de l’Enseignement Supérieur et de la Recherche, Direction Générale de la Recherche et de l’Innovation under the number 2020-007, as well as by the ethic committee of the Foch Hospital (IRB00012437). Written informed consent for participation was not required for this study in accordance with the national legislation and the institutional requirements.

## Author contributions

Conceptualization, IS-C and ES, Legal aspects and authorization, ES, Resources, ES, Investigation with surgery, JW, MG, JE, MLG., Investigation with tissue and cell processing, CG, CU, BC, CC, DD, IS-C., Investigation with bank construction, CB, CG, CU, IS-C, Software and Formal analysis using Bio-statistics and informatics, T-PM, LJ, LH, Formal analysis, MD, T-PM, LJ, LH, CG, IS-C. Visualization, CG, IS-C, LJ, T-PM, Writing – original draft IS-C, Writing edits T-PM, MD, Supervision, IS-C, MD,T-PM Project administration, IS-C,ES, AR, AM, Funding acquisition, IS-C and ES. “All authors contributed to the article and approved the submitted version.
