# Supplementary material for: Cell type- and time-dependent biological responses in ex vivo perfused lung grafts
Source: Front Immunol. 2023 Jul 3;14:1142228. doi: 10.3389/fimmu.2023.1142228 (PMC10351384; doi:10.3389/fimmu.2023.1142228)

**Additional file 15. Heat map of the expression modulation of the genes contributing to selected IPA pathways and functions across the cell subtypes of the myeloid cell family**. For pathways and functions of the IPA results mentioned in the main body text, a list of contributing genes was established from the union of the cases with absolute z-scores > 1.9. The gene expression fold changes (log2) of the contributing gene list is illustrated as a heat map, based on the shown scale. For monocytes/macrophages, the pathways/functions illustrated are : Attraction of Phagocytes, Binding of Leukocytes, Inflammation of Lung, Apoptosis, GnRH signaling. For conventional type 2 dendritic cells, the Dendritic cell maturation is illustrated. Arrows point to the genes mentioned in the main text.

Myeloid cells – Alveolar macrophages, monocyte-derived macrophages, classical monocytes and non-classical monocytes – Attraction of phagocytes


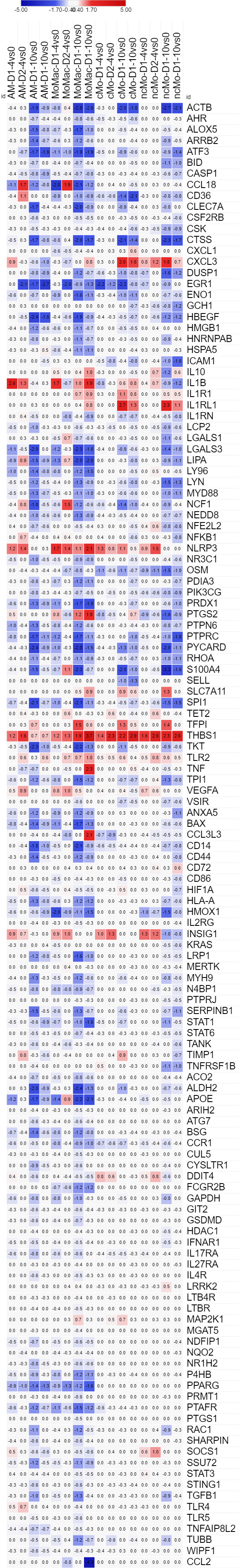


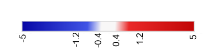


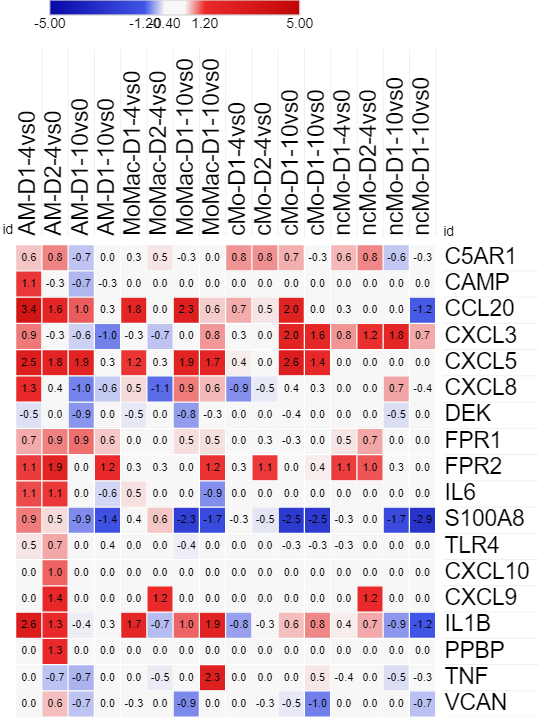


Myeloid cells – Alveolar macrophages, monocyte-derived macrophages, classical monocytes and non-classical monocytes – Inflammation of lung


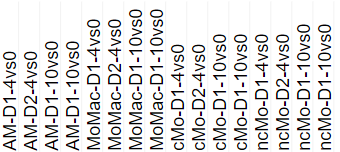

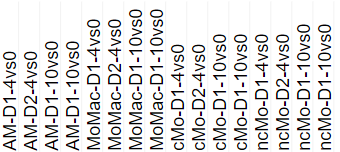


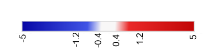

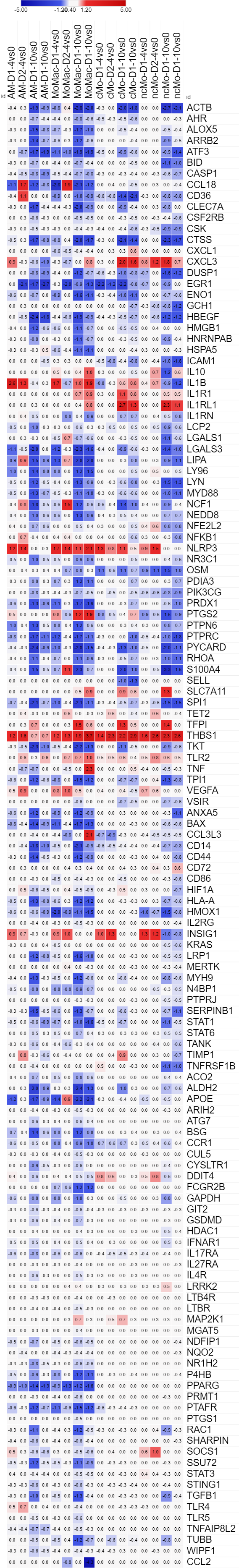

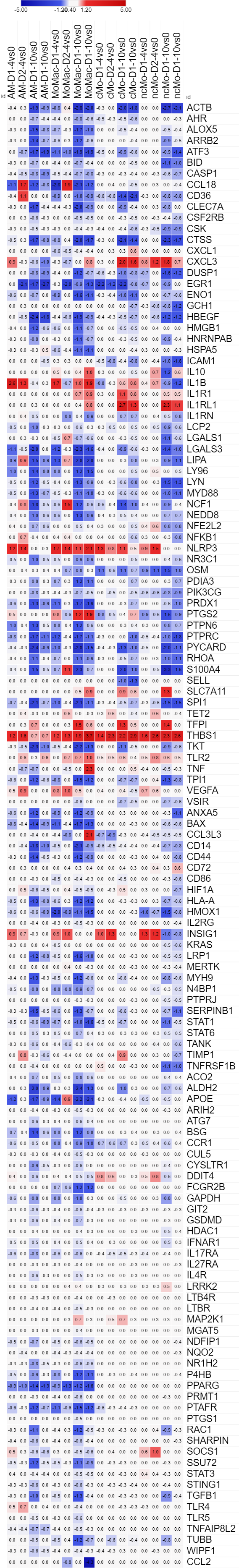


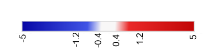
Myeloid cells – Alveolar macrophages, monocyte-derived macrophages, classical monocytes and non-classical monocytes – Binding of leukocytes


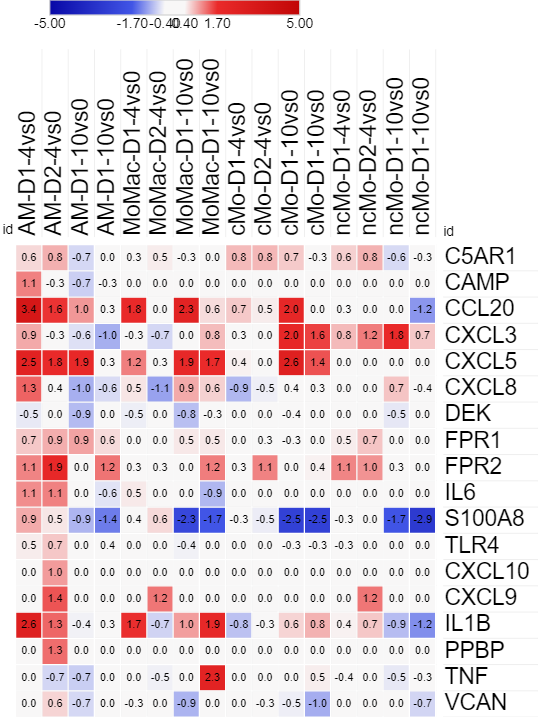

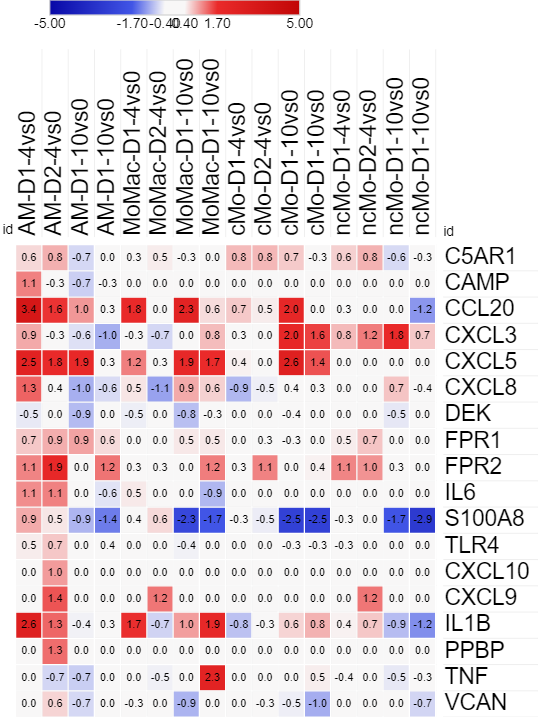

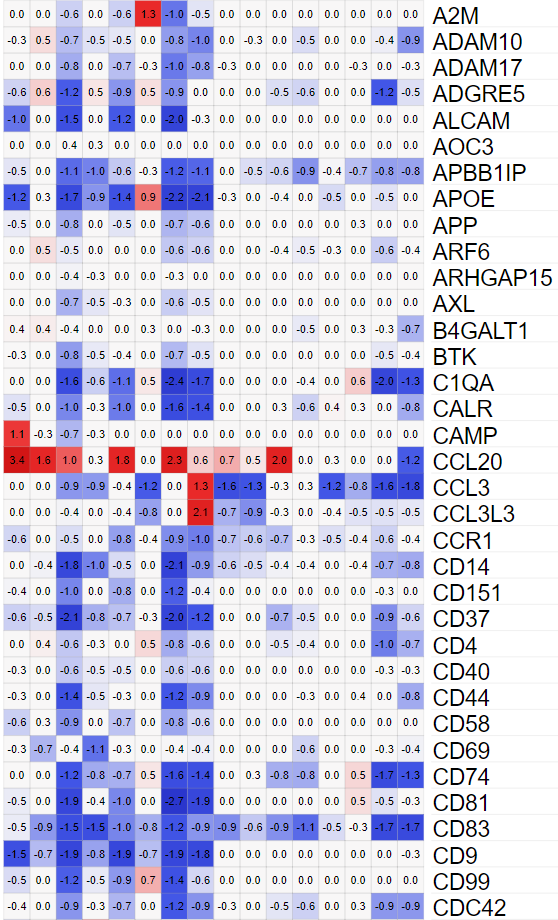

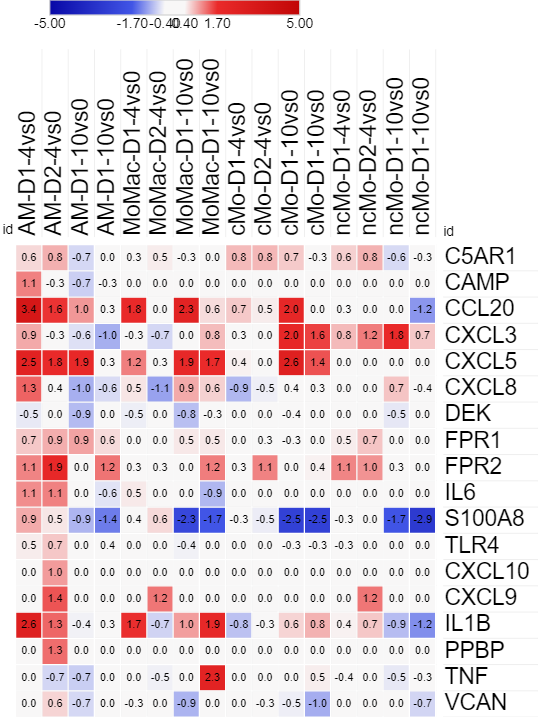


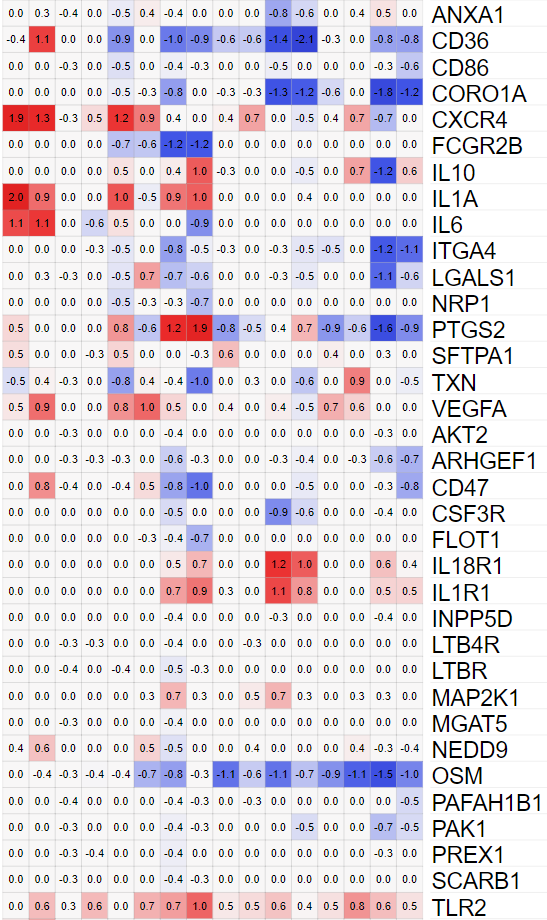

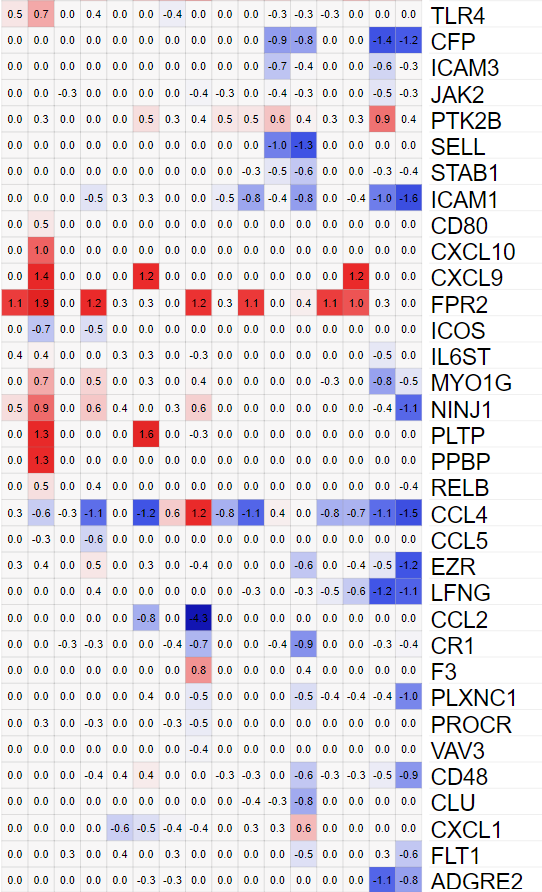

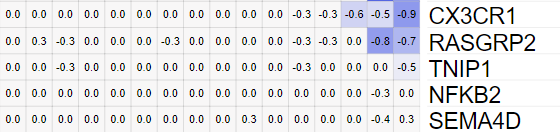

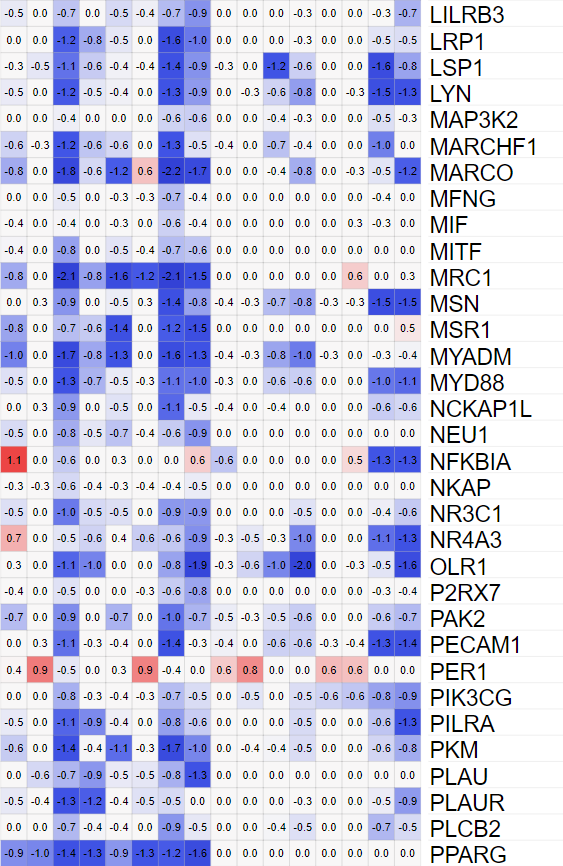

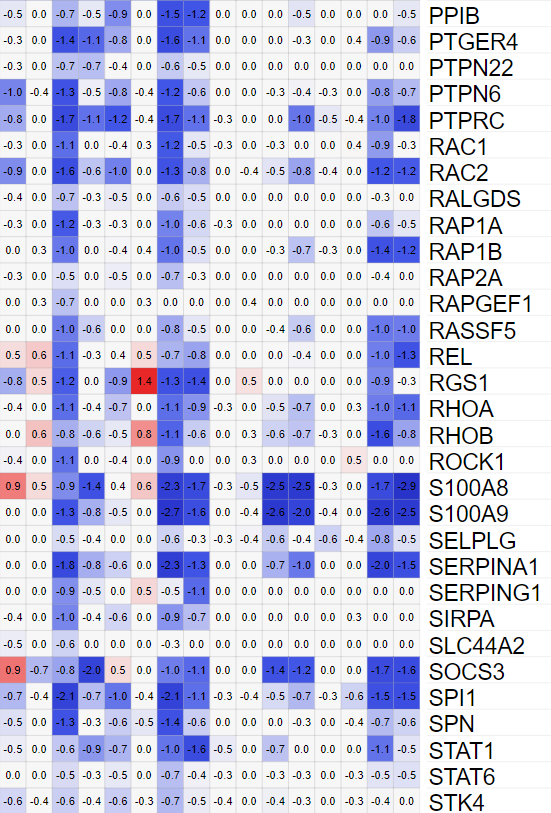

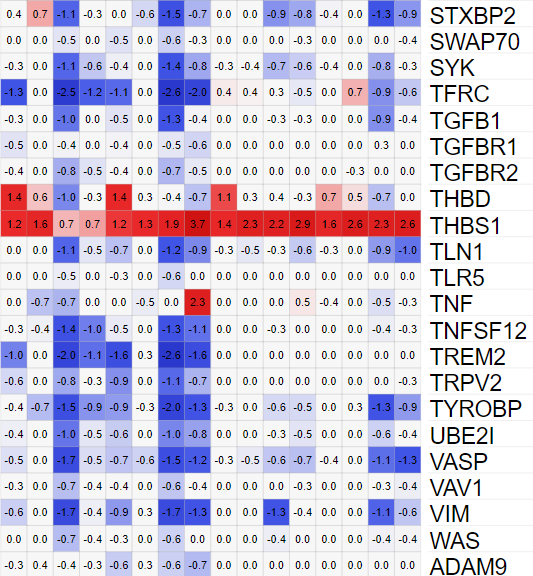

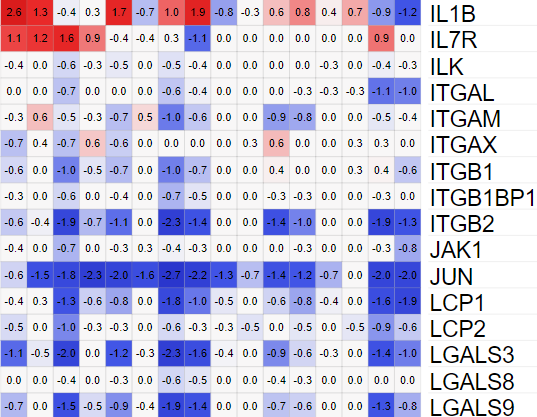

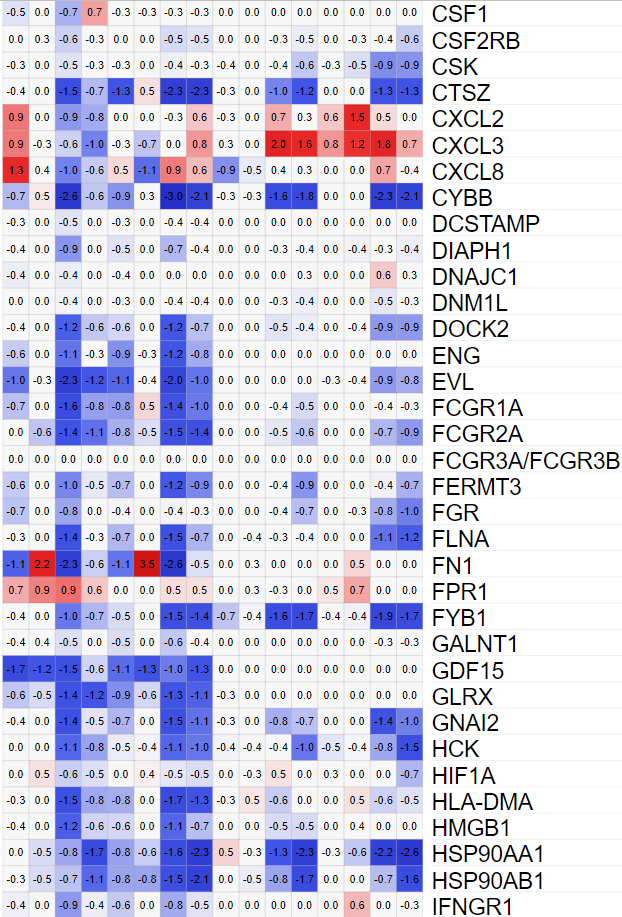


Myeloid cells – Alveolar macrophages, monocyte-derived macrophages, classical monocytes and non-classical monocytes – Apoptosis (page 1)


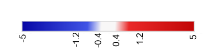

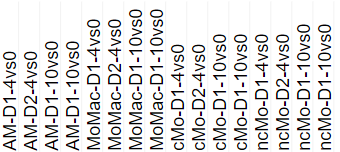

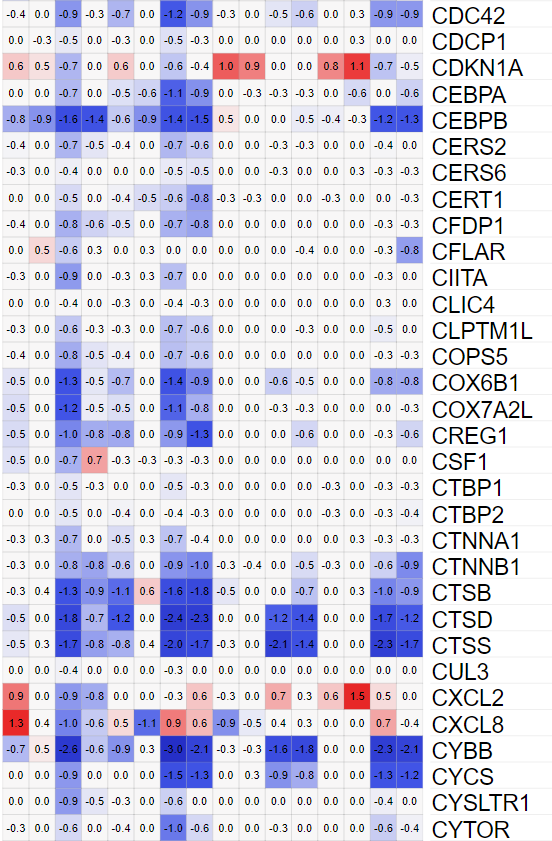

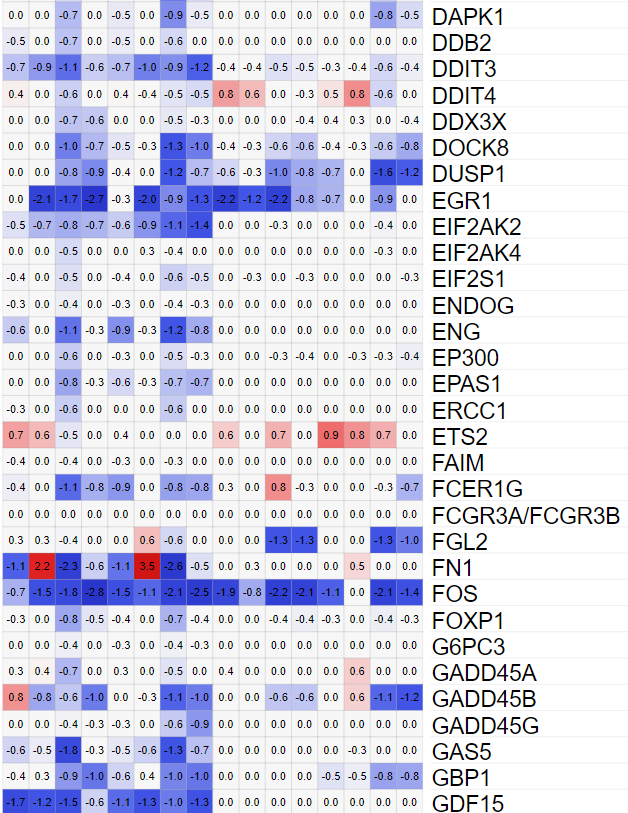

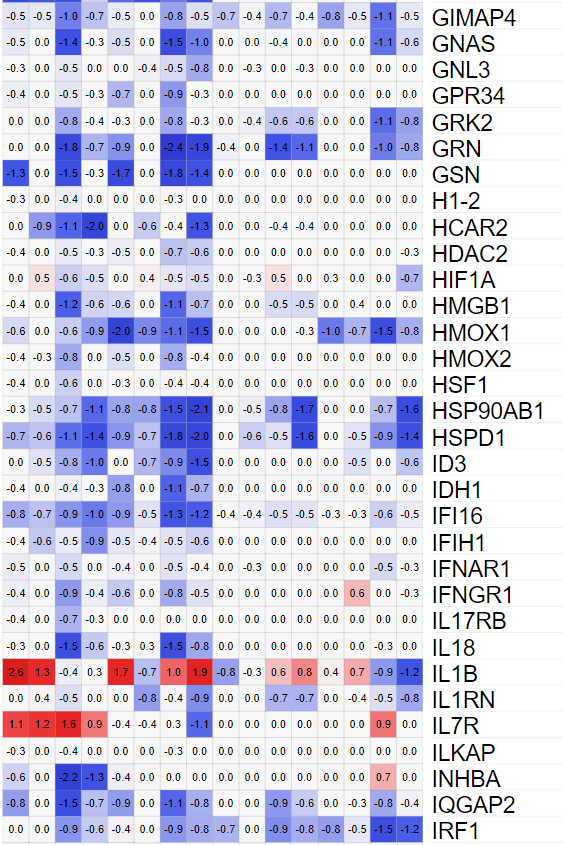

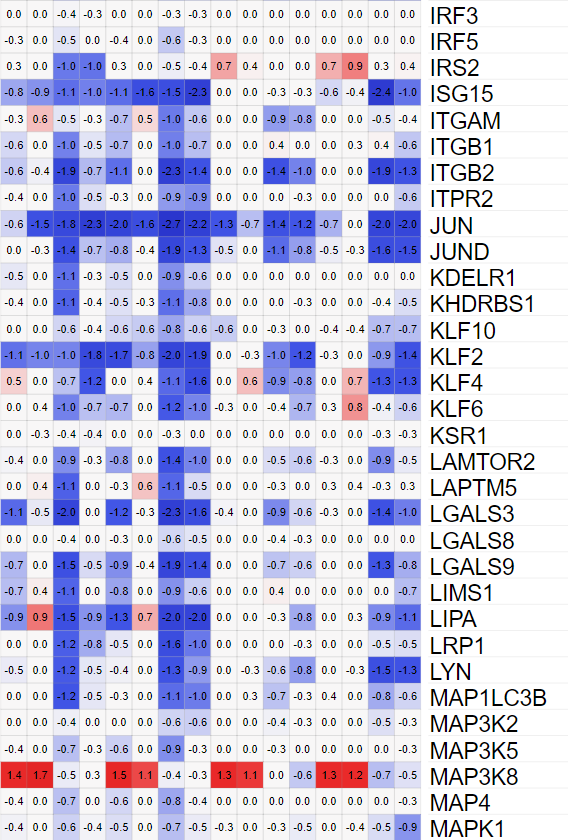

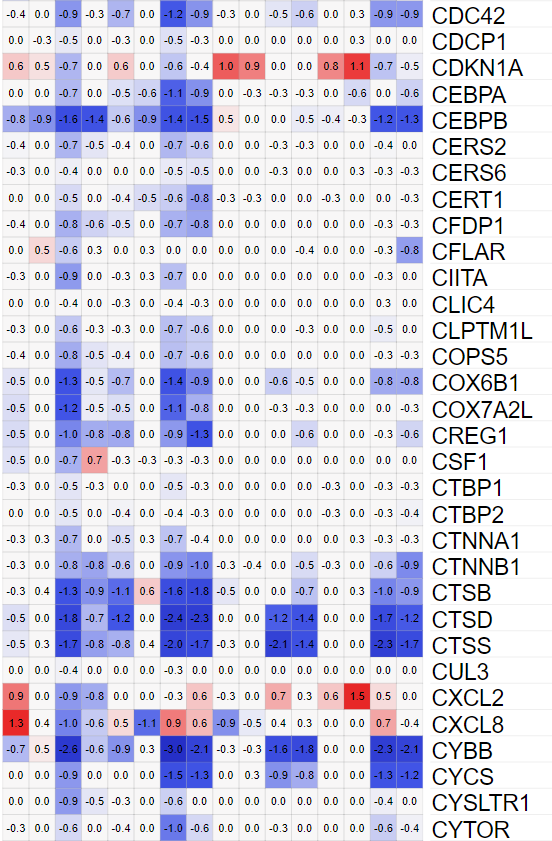

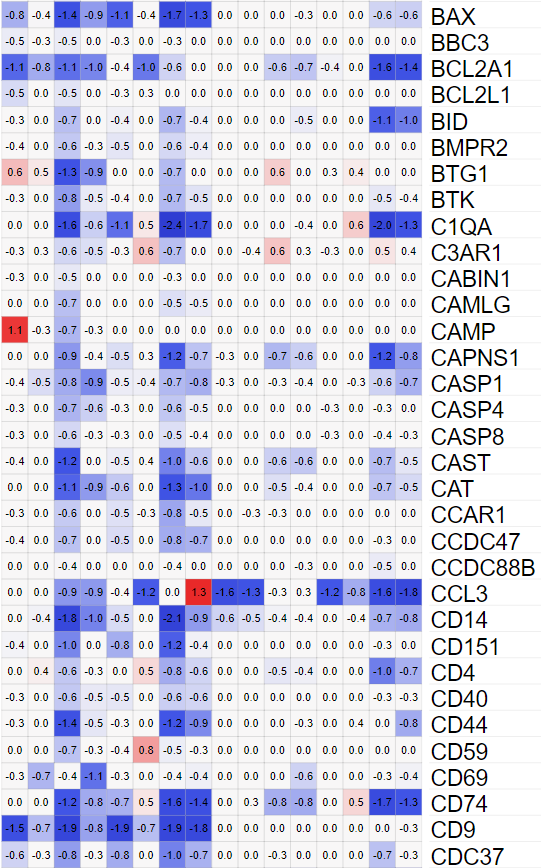

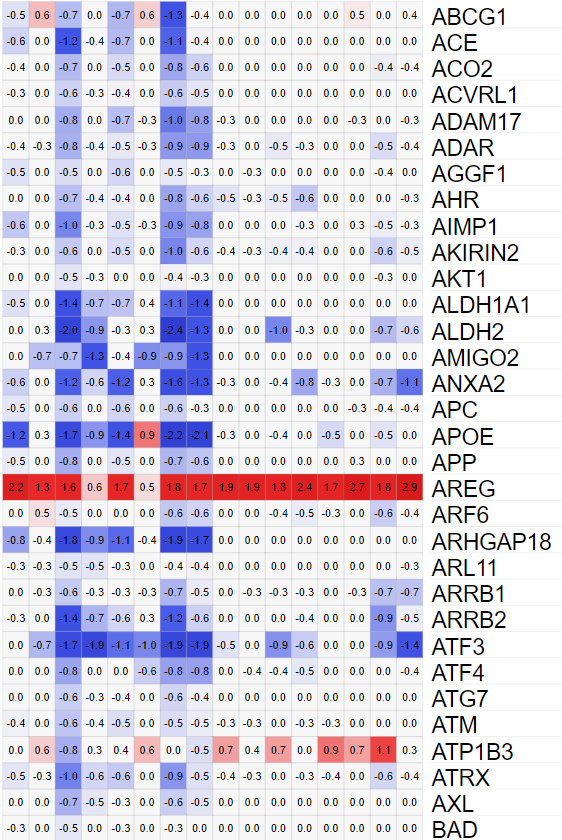

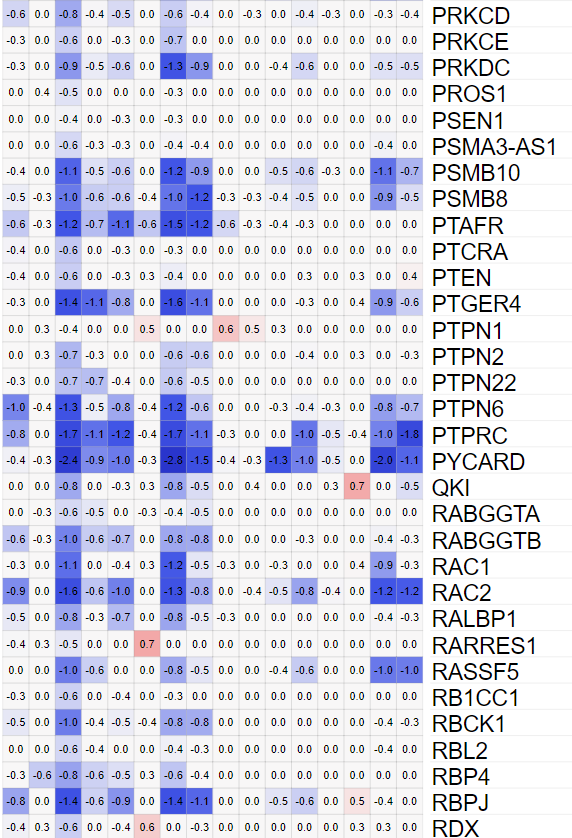

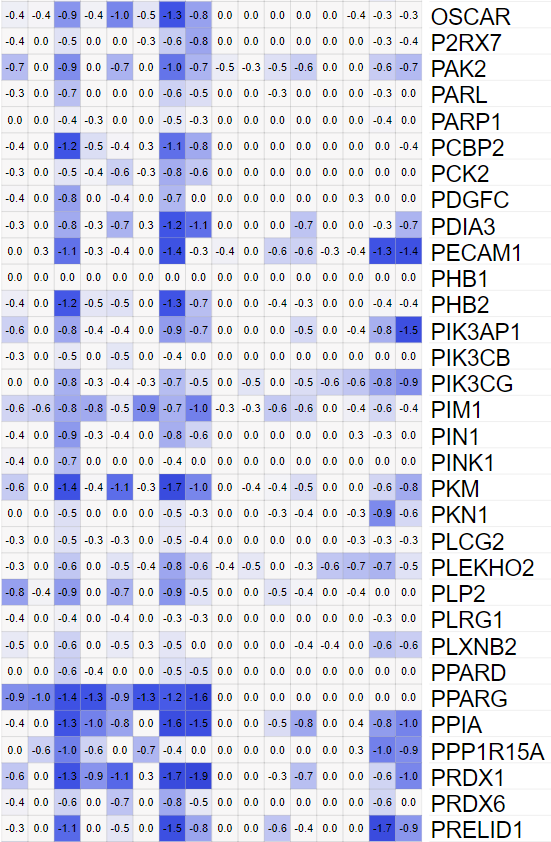

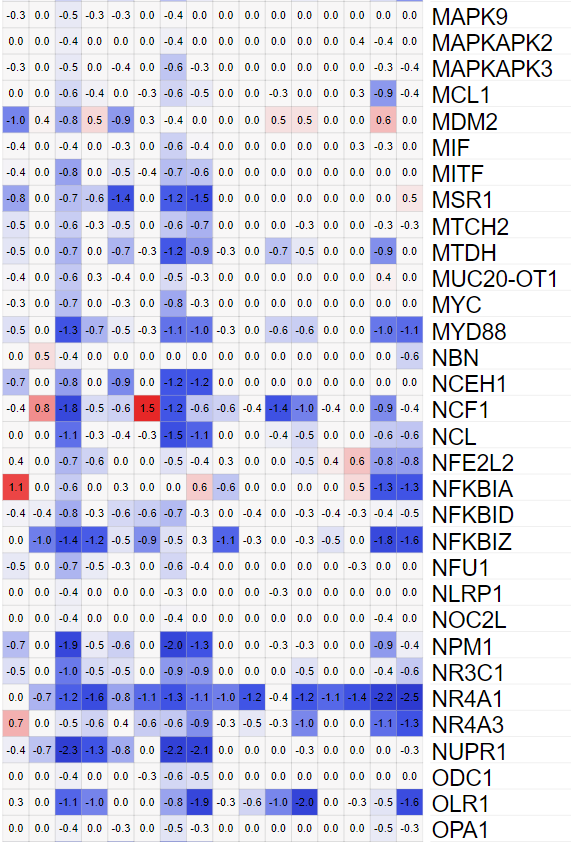

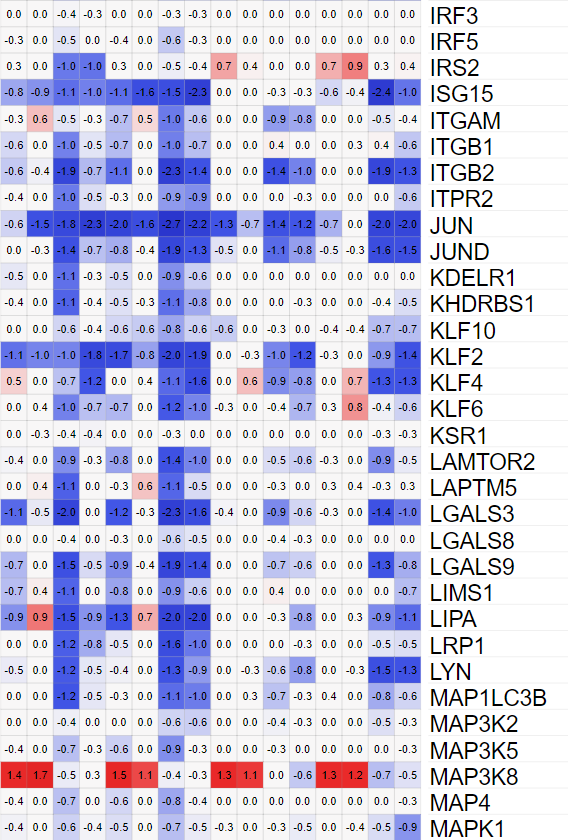

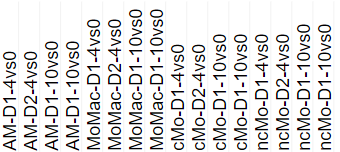

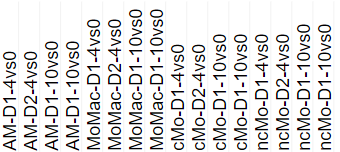


Myeloid cells – Alveolar macrophages, monocyte-derived macrophages, classical monocytes and non-classical monocytes – Apoptosis (page 2)


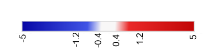

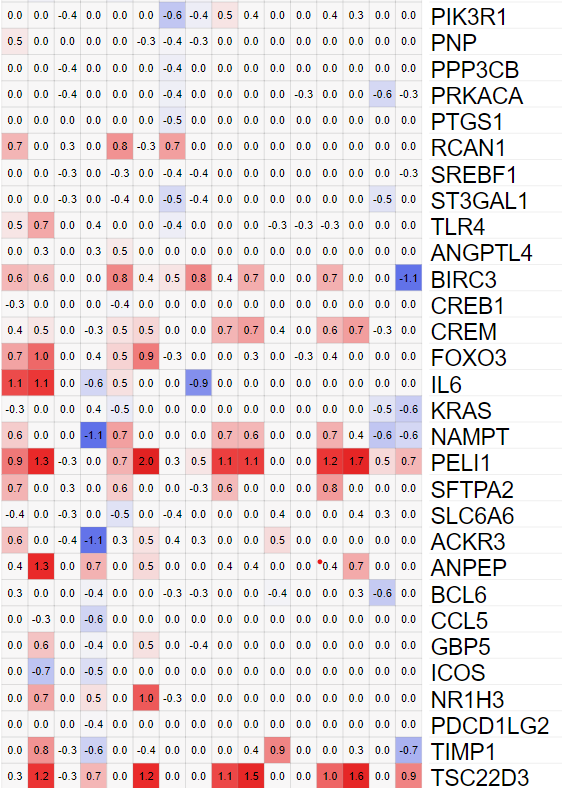

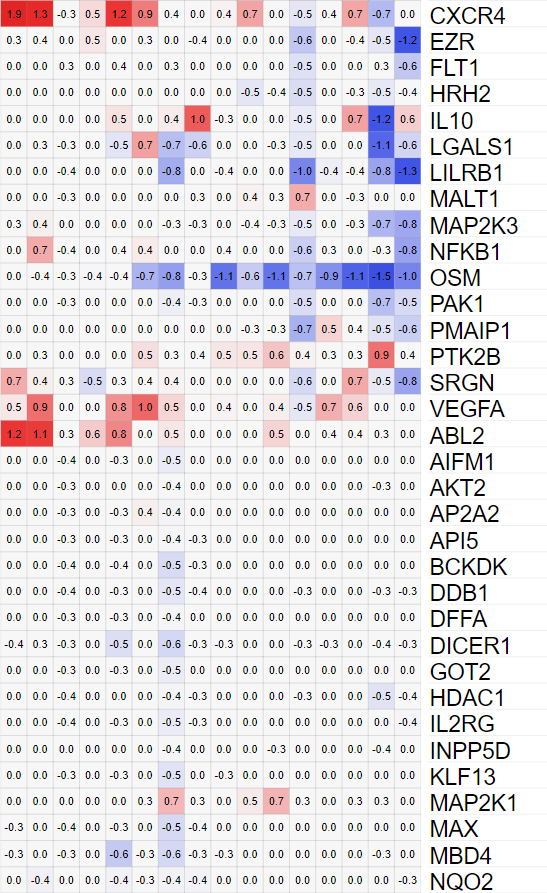

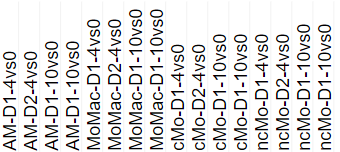

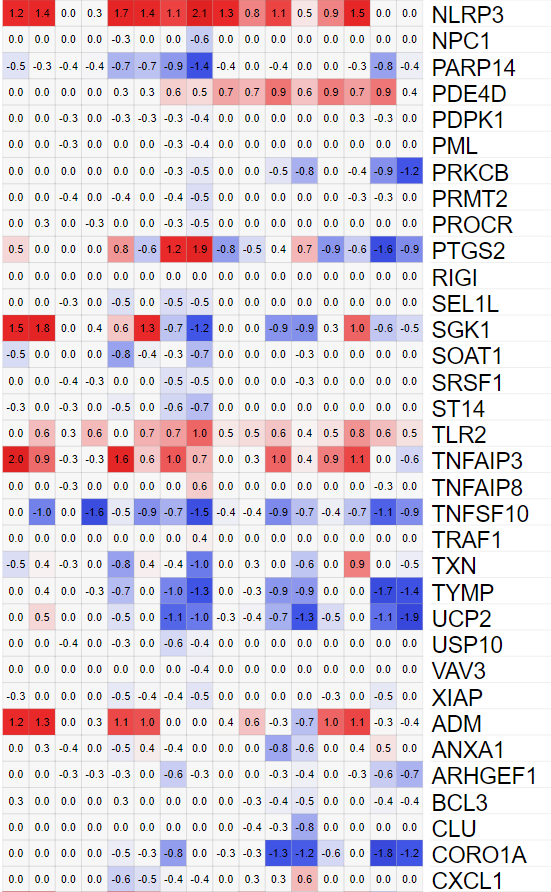

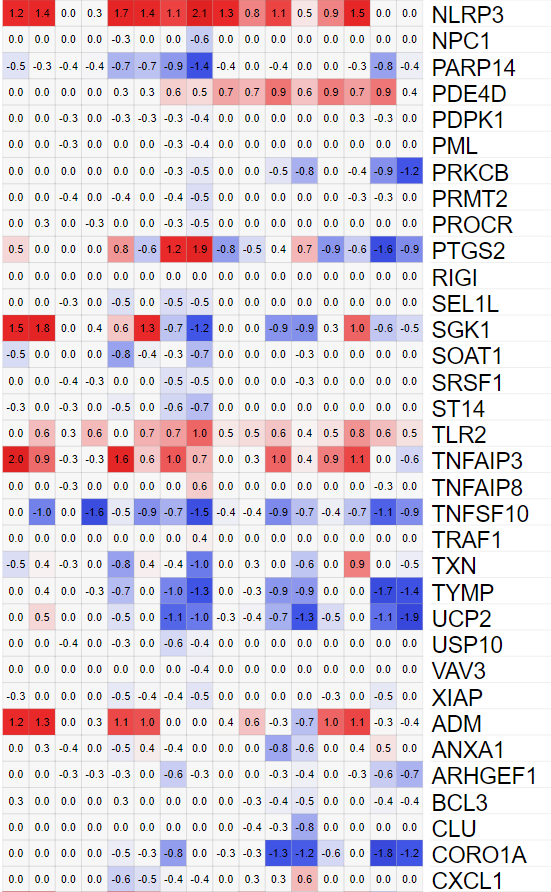

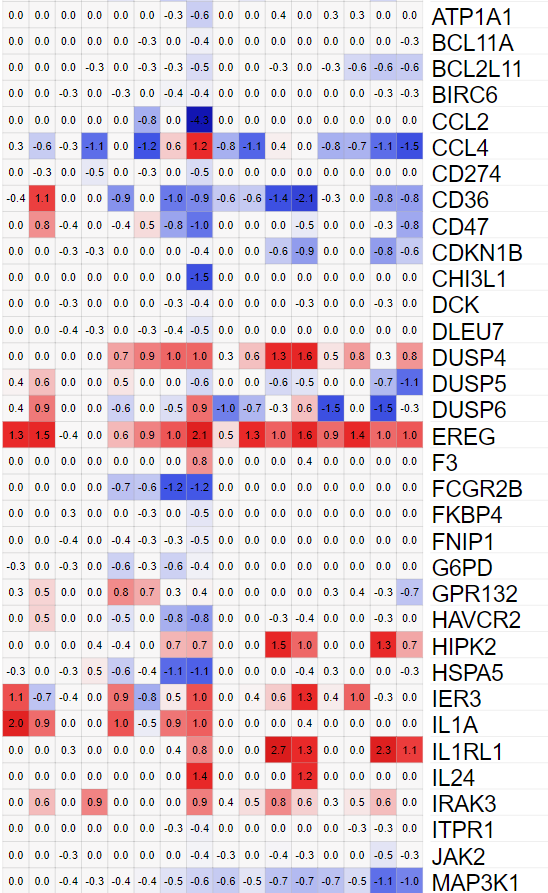

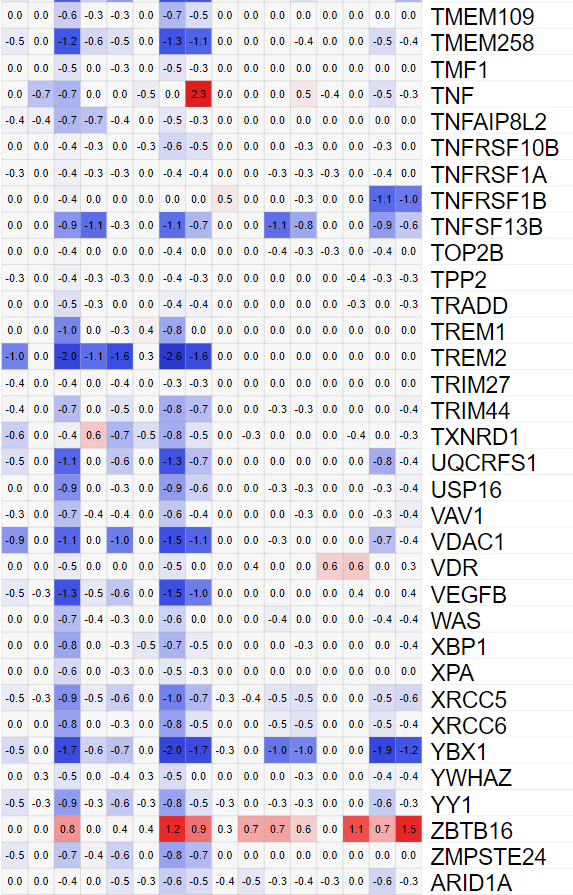

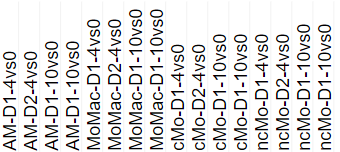

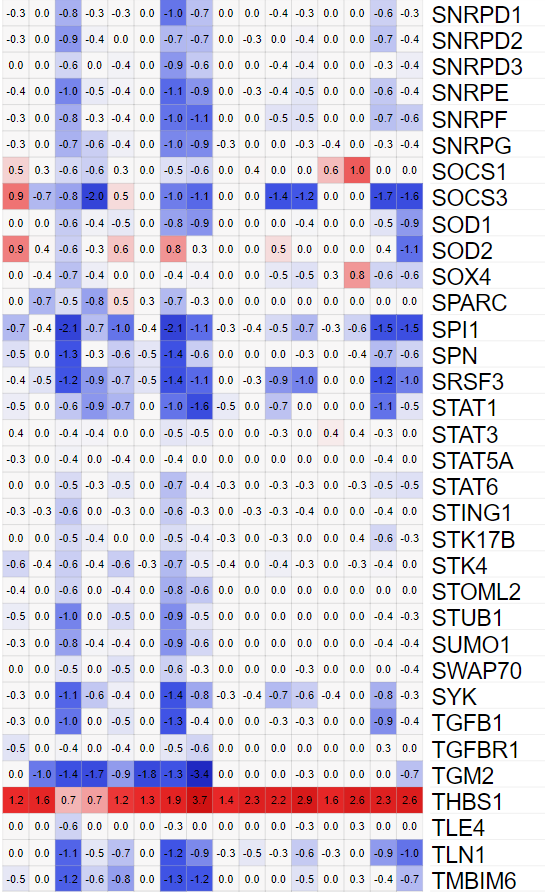

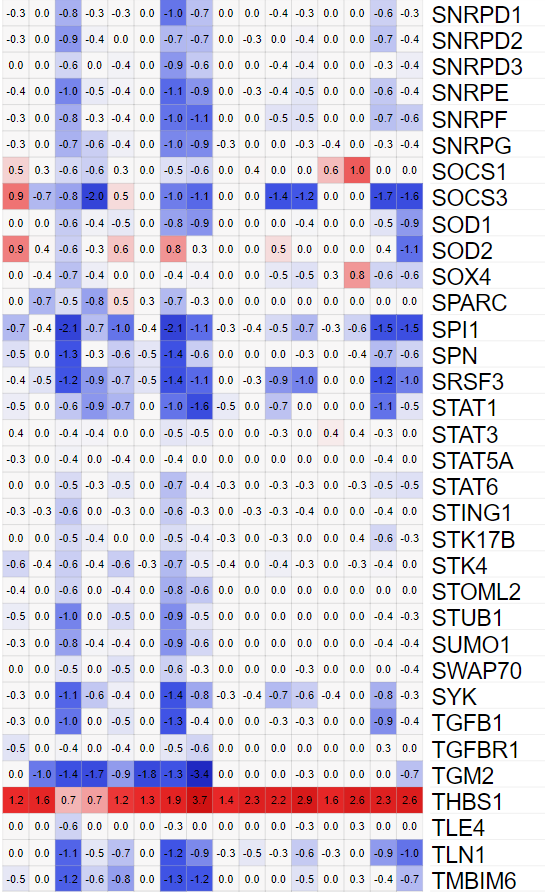

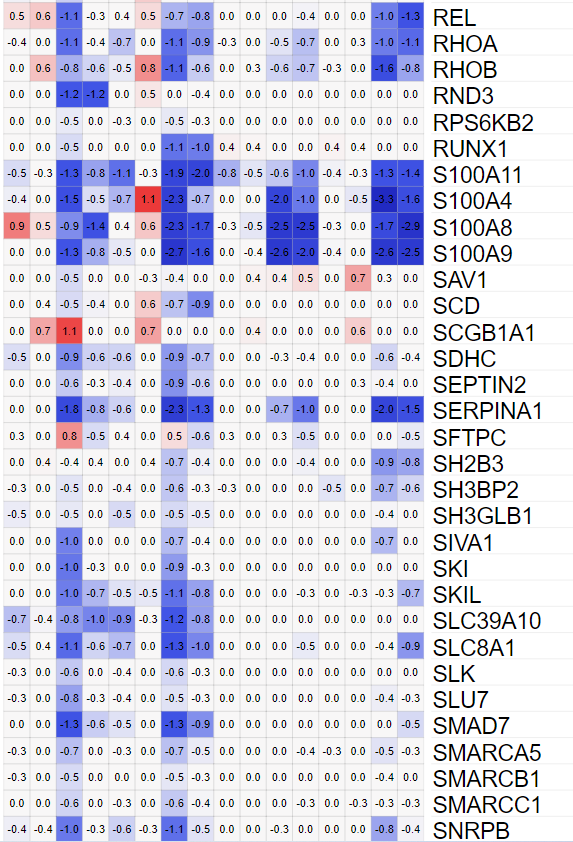

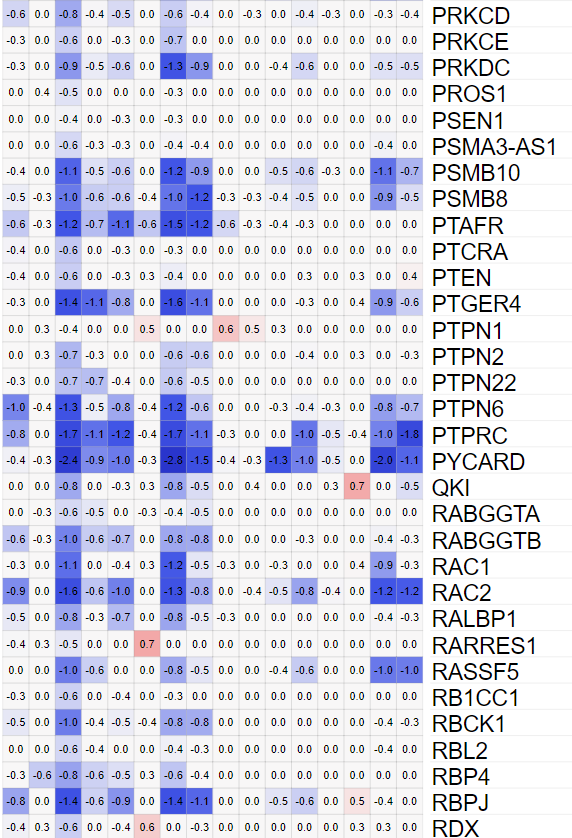

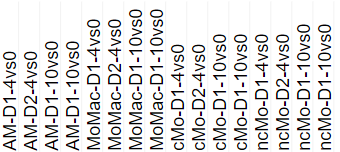


Myeloid cells – Alveolar macrophages, monocyte-derived macrophages, classical monocytes and non-classical monocytes – GNRH signaling


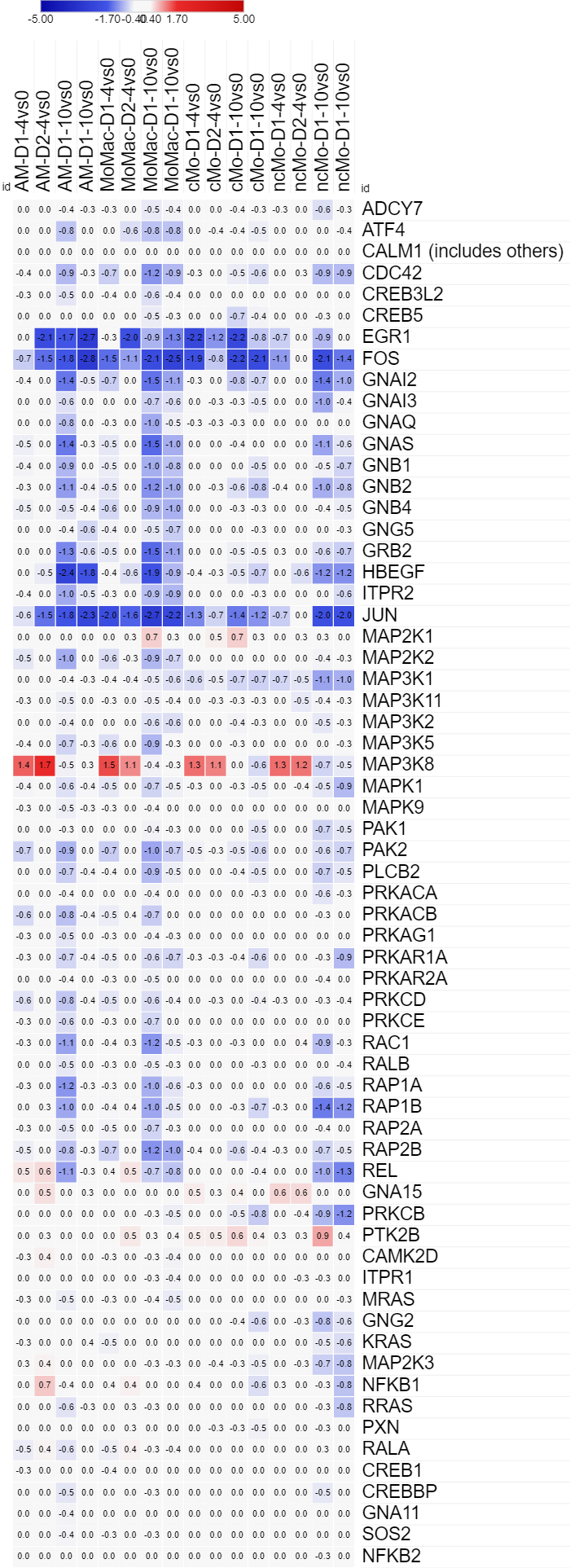


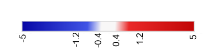


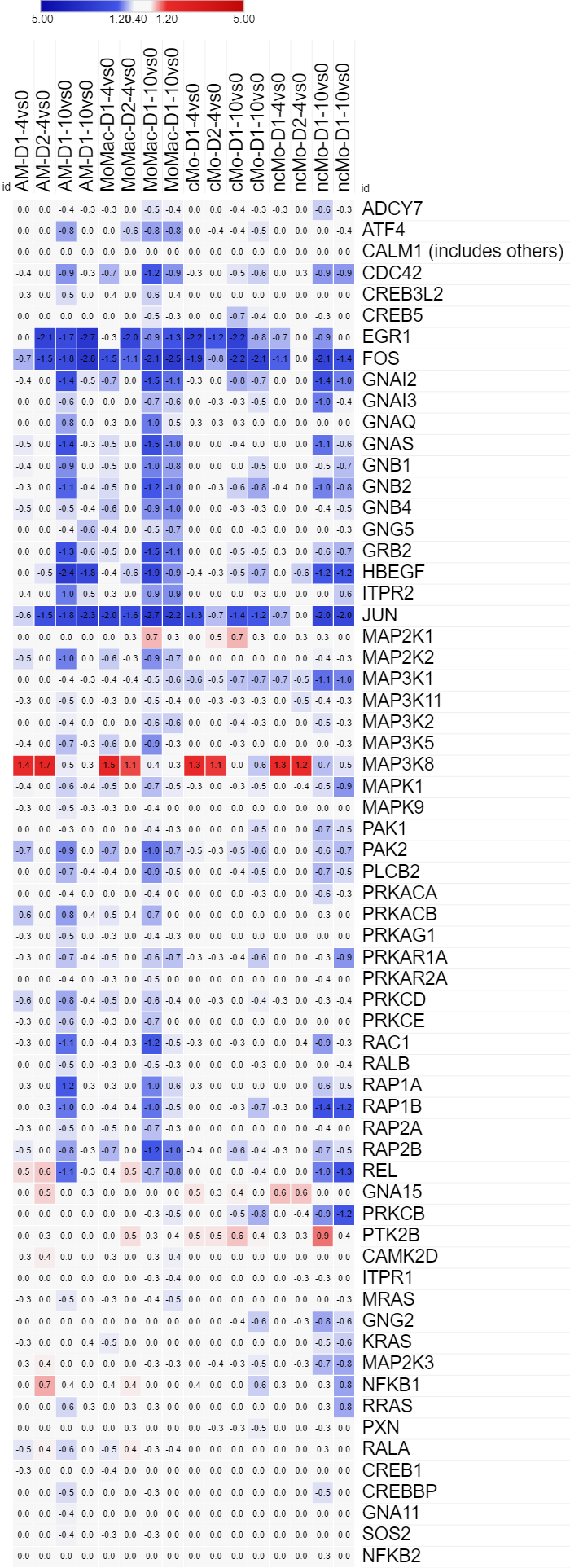


Myeloid cells – Conventional type 2 dendritic cells – Dendritic cell maturation


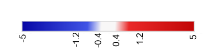

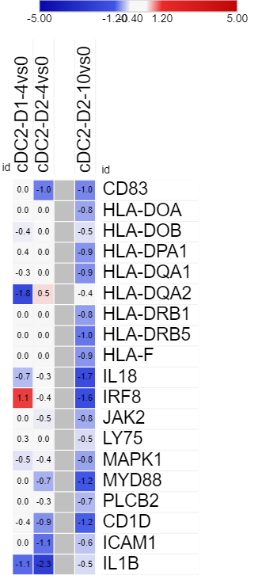

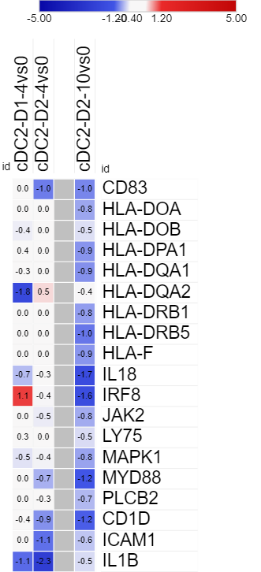

Supplement: Supplementary file 1 [file DataSheet_1.zip › Additional file-Data Sheet 1/Additional file 15-Contributing genes Mo-MP-DC.docx]
