# Supplementary material for: Cell type- and time-dependent biological responses in ex vivo perfused lung grafts
Source: Front Immunol. 2023 Jul 3;14:1142228. doi: 10.3389/fimmu.2023.1142228 (PMC10351384; doi:10.3389/fimmu.2023.1142228)
Supplement: Supplementary file 1 [file DataSheet_1.zip › Additional file-Data Sheet 1/Additional file 9-Proportion Identities.pptx]

## Slide 1
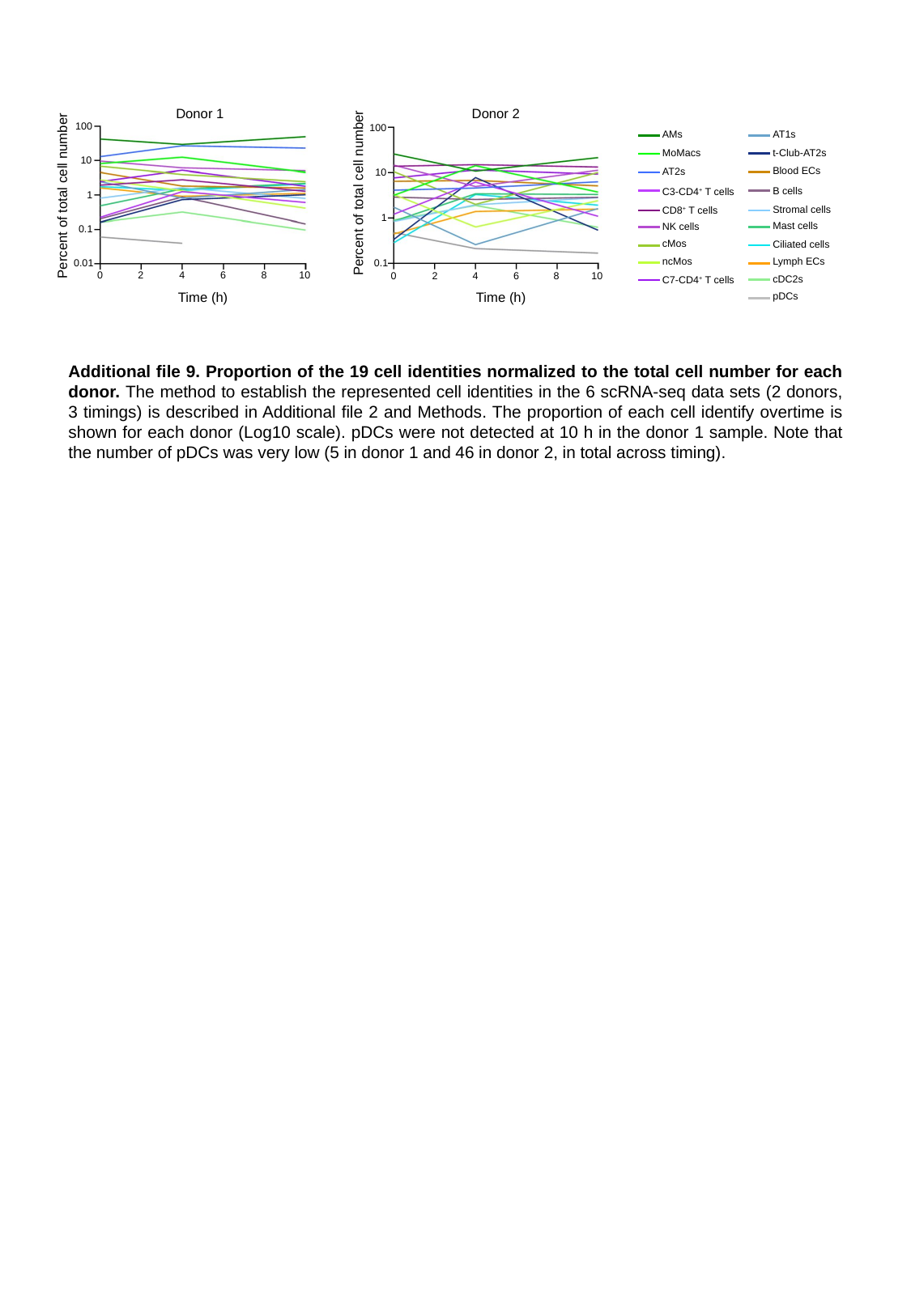

Donor 1
Donor 2
 100
 1
 0.1
 0.01
0
2
4
6
8
10
Time (h)
 100
AMs
AT1s
MoMacs
t-Club-AT2s
 10
Blood ECs
AT2s
 10
B cells
Percent of total cell number
C3-CD4+ T cells
Percent of total cell number
Stromal cells
CD8+ T cells
 1
Mast cells
NK cells
cMos
Ciliated cells
ncMos
Lymph ECs
 0.1
0
2
4
6
8
10
cDC2s
C7-CD4+ T cells
Time (h)
pDCs
Additional file 9. Proportion of the 19 cell identities normalized to the total cell number for each donor. The method to establish the represented cell identities in the 6 scRNA-seq data sets (2 donors, 3 timings) is described in Additional file 2 and Methods. The proportion of each cell identify overtime is shown for each donor (Log10 scale). pDCs were not detected at 10 h in the donor 1 sample. Note that the number of pDCs was very low (5 in donor 1 and 46 in donor 2, in total across timing).
