# Supplementary material for: Cell type- and time-dependent biological responses in ex vivo perfused lung grafts
Source: Front Immunol. 2023 Jul 3;14:1142228. doi: 10.3389/fimmu.2023.1142228 (PMC10351384; doi:10.3389/fimmu.2023.1142228)
Supplement: Supplementary file 1 [file DataSheet_1.zip › Additional file-Data Sheet 1/Additional file 8-Gene symbols.docx]

***Additional file 8. Gene symbols from NCBI and their expanded gene names***

*-ACTB, ACTG1: Actin B, Actin G1*

*-ARPC1A, 2, 3, 4, 5: Actin related Protein complex Subunit 1A/B, 2, 3, 4, 5*

*-ACTR2, ACTR3: Actin-Related 2, Actin-Related 3*

*-AGER: Advanced Glycosylation End-Product Specific Receptor*

*-BAX: BCL2 Associated X, Apoptosis Regulator*

*-BCL2L1, BCL2L10, BCL2L11A: BCL2-like 1, 10, 11A*

*-C1QB: Complement C1q B Chain)*

*-C5AR1: Complement C5a Receptor*

*-CASP4: Caspase 4*

*-CAV1Caveolin 1 => compléter à partir des tClubAT2*

*-CCL2, CCL14, CCL20, CCL21: C-C Motif Chemokine Ligand 2, C-C Motif Chemokine Ligand 14, C-C Motif Chemokine Ligand 20, C-C Motif Chemokine Ligand 21*

*-CD3D: CD3 Delta Subunit Of T-Cell Receptor Complex*

*-CEACAM6 : CEA Cell Adhesion Molecule 6*

*-CLEC10A: C-Type Lectin Domain Containing 10A*

*-CLIC3: Chloride Intracellular Channel 3*

*- CFP: Complement Factor Properdin*

*-COL1A2, COL6A2: collagen 1A2, collagen 6A2*

*-CPA3: Carboxypeptidase A3*

*-CSF3: Colony Stimulating Factor 3*

*-CTLA4: Cytotoxic T-Lymphocyte Associated Protein 4*

*-CXCL1, CXCL2, CXCL3, CXCL5, CXCL8, CXCL16: C-X-C- Motif Chemokine Ligand 1, C-X-C- Motif Chemokine Ligand 2, C-X-C- Motif Chemokine Ligand 3, C-X-C- Motif Chemokine Ligand 5, C-X-C- Motif Chemokine Ligand 8, C-X-C- Motif Chemokine Ligand 16*

*-EDNRB: Endothelin Receptor Type B*

*-EFNB2: Ephrin B2*

*-FABP4: Fatty Acid Binding Protein 4*

*-FBLN1: Fibulin 1*

*-FCN1: Ficolin 1*

*-FGL2 : Fibrinogen Like 2*

*-FCER1A: Fc Epsilon Receptor Ia*

*-GADD45A: Growth Arrest And DNA Damage Inducible Alpha*

*-GNLY: Granulysin*

*-GZMB,A: granzyme B, A*

*-IGKC, HC: Immunoglobulin Kappa Constant, Immunoglobulin Gamma Constant*

*-ICAM1: Intercellular Adhesion Molecule 1*

*-IFNG: interferon gamma*

*-IGFBP6: Insulin Like Growth Factor Binding Protein 6*

*-IL1A, IL1B, 6: Interleukin 1A, Interleukin 1B, Interleukin 6*

*-IL1R1 : Interleukin 1 Receptor 1*

*-IRF7, 8: Interferon Regulatory Factor 7, 8*

*-ITGA4: Integrin Subunit Alpha 4*

*-ITGB1, ITGB2: Integrin Subunit Beta 1, Integrin Subunit Beta 2*

*-KIT: KIT proto-oncogene*

*-LILRB2: Leukocyte Immunoglobulin Like Receptor B2*

*-LYVE1: Lymphatic Vessel Endothelial Hyaluronan Receptor 1*

*-MARCO: Macrophage Receptor With Collagenous Structure*

*-MIF: Macrophage Migration Inhibitory factor*

*-MGP: Matrix Gla Protein*

*-MRC1: Mannose Receptor C-Type 1*

*-MT1E, MT2A: Metallothionein 1E, Metallothionein 2A*

*-MYO5C: myosin 5C*

*-MYL: myosin-like genes*

*-NKG7: Natural Killer Cell Granule Protein 7*

*-NR2F2: Nuclear Receptor Subfamily 2 Group F Member 2*

*-PDPN:podoplanin*

*-PIFO:Primary Cilia Formation*

*-PIGR: Polymeric Immunoglobulin Receptor)*

*-PLAT: Plasminogen Activator, Tissue Type*

*-PLAUR: Plasminogen Activator, Urokinase Receptor*

*- PRKCE: Protein Kinase C Epsilon*

*-RHOA: Ras Homolog Family Member A*

*-S100A8: S100 Calcium Binding Protein A8*

*-SCGB3A1, SCGB1A1: Secretoglobin Family 3A Member 1, Secretoglobin Family 1A Member 1*

*-SOCS1 : Suppressor Of Cytokine Signaling 1*

*-SFTPB, C: Surfactant Protein B, C*

*-TCF4 : Transcription Factor 4*

*-TPSAB1, TPSB2 : Tryptase Alpha/Beta 1, Tryptase Beta 2*

*-TFPI:Tissue Factor Pathway Inhibitor*

*-TNFAIP3 : Alpha Induced Protein 3*

*-TRBC1: T Cell Receptor Beta Constant 1*

*-TNFA: Tumor Necrosis Factor Alpha*

*-TREM1: Triggering Receptor Expressed On Myeloid Cells 1*

*-VCAM1: Vascular Adhesion Molecule 1*

*-VCAN : Versican*

*-VIM: vimentin*

*-VWF: Von Willebrand Factor*
