# Supplementary material for: Cell type- and time-dependent biological responses in ex vivo perfused lung grafts
Source: Front Immunol. 2023 Jul 3;14:1142228. doi: 10.3389/fimmu.2023.1142228 (PMC10351384; doi:10.3389/fimmu.2023.1142228)
Supplement: Supplementary file 1 [file DataSheet_1.zip › Additional file-Data Sheet 1/Additional file 4-QC.pptx]

## Slide 1
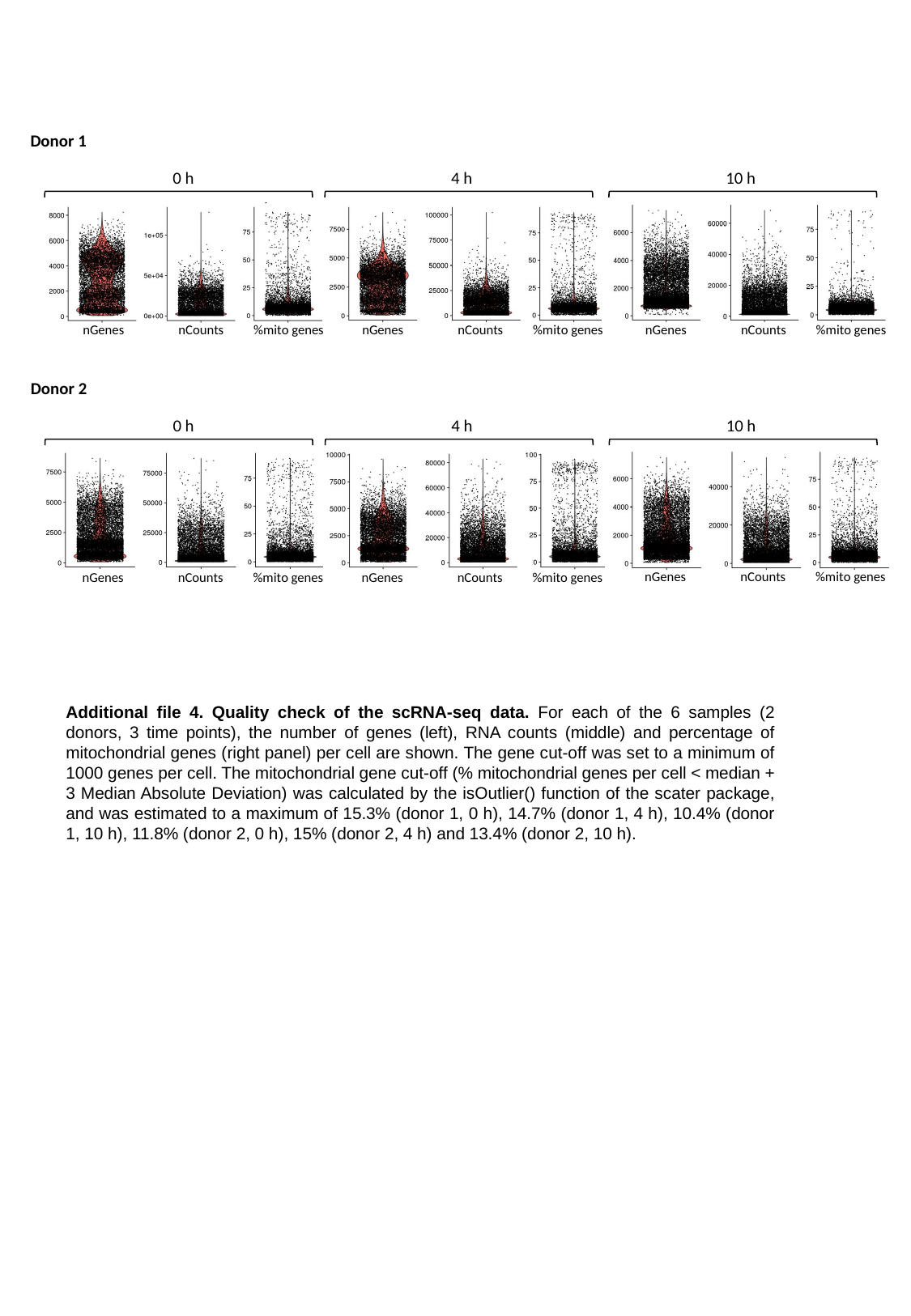

Donor 1
0 h
4 h
10 h
nGenes
nCounts
%mito genes
nGenes
nCounts
%mito genes
nGenes
nCounts
%mito genes
Donor 2
0 h
4 h
10 h
nGenes
nCounts
%mito genes
nGenes
nCounts
%mito genes
nGenes
nCounts
%mito genes
Additional file 4. Quality check of the scRNA-seq data. For each of the 6 samples (2 donors, 3 time points), the number of genes (left), RNA counts (middle) and percentage of mitochondrial genes (right panel) per cell are shown. The gene cut-off was set to a minimum of 1000 genes per cell. The mitochondrial gene cut-off (% mitochondrial genes per cell < median + 3 Median Absolute Deviation) was calculated by the isOutlier() function of the scater package, and was estimated to a maximum of 15.3% (donor 1, 0 h), 14.7% (donor 1, 4 h), 10.4% (donor 1, 10 h), 11.8% (donor 2, 0 h), 15% (donor 2, 4 h) and 13.4% (donor 2, 10 h).
