# Supplementary material for: Cell type- and time-dependent biological responses in ex vivo perfused lung grafts
Source: Front Immunol. 2023 Jul 3;14:1142228. doi: 10.3389/fimmu.2023.1142228 (PMC10351384; doi:10.3389/fimmu.2023.1142228)
Supplement: Supplementary file 1 [file DataSheet_1.zip › Additional file-Data Sheet 1/Additional file 6-Integrated UMAP-filtration.pptx]

## Slide 1
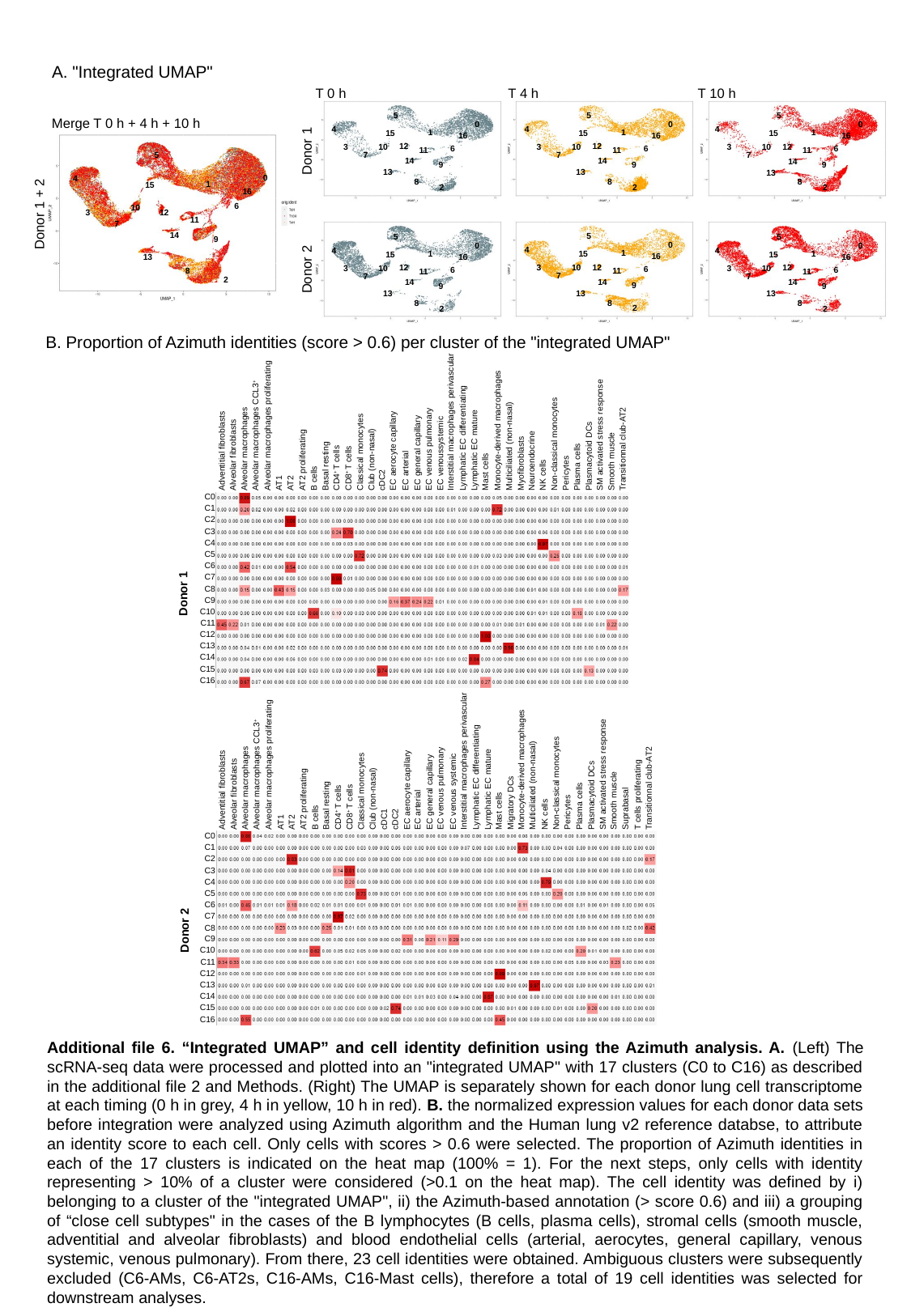

A. "Integrated UMAP"
T 4 h
T 0 h
T 10 h
5
0
4
1
15
16
12
10
3
6
11
7
14
9
13
8
2
5
0
4
1
15
16
12
10
3
6
11
7
14
9
13
8
2
5
0
4
1
15
16
12
10
3
6
11
7
14
9
13
8
2
Merge T 0 h + 4 h + 10 h
Donor 1
5
0
4
1
15
16
6
10
Donor 1 + 2
3
12
11
7
14
5
0
4
1
15
16
12
10
3
6
11
7
14
9
13
8
2
5
0
4
1
15
16
12
10
3
6
11
7
14
9
13
8
2
5
0
4
1
15
16
12
10
3
6
11
7
14
9
13
8
2
9
13
Donor 2
8
2
B. Proportion of Azimuth identities (score > 0.6) per cluster of the "integrated UMAP"
Alveolar macrophages proliferating
AT1
AT2
AT2 proliferating
B cells
Basal resting
CD4+ T cells
CD8+ T cells
Classical monocytes
Club (non-nasal)
cDC2
EC aerocyte capillary
EC arterial
EC general capillary
EC venous pulmonary
EC venoussystemic
Interstitial macrophages perivascular
Lymphatic EC differentiating
Lymphatic EC mature
Mast cells
Monocyte-derived macrophages
Multiciliated (non-nasal)
Myofibroblasts
Neuroendocrine
NK cells
Non-classical monocytes
Pericytes
Plasma cells
Plasmacytoid DCs
SM activated stress response
Smooth muscle
Transitionnal club-AT2
Alveolar macrophages CCL3+
Adventitial fibroblasts
Alveolar fibroblasts
Alveolar macrophages
C0
C1
C2
C3
C4
C5
C6
C7
C8
C9
C10
C11
C12
C13
C14
C15
C16
Adventitial fibroblasts
Alveolar fibroblasts
Alveolar macrophages
Alveolar macrophages CCL3+
Alveolar macrophages proliferating
AT1
AT2
AT2 proliferating
B cells
Basal resting
CD4+ T cells
CD8+ T cells
Classical monocytes
Club (non-nasal)
cDC1
cDC2
EC aerocyte capillary
EC arterial
EC general capillary
EC venous pulmonary
EC venous systemic
Interstitial macrophages perivascular
Lymphatic EC differentiating
Lymphatic EC mature
Mast cells
Migratory DCs
Monocyte-derived macrophages
Multiciliated (non-nasal)
NK cells
Non-classical monocytes
Pericytes
Plasma cells
Plasmacytoid DCs
SM activated stress response
Smooth muscle
Suprabasal
T cells proliferating
Transitionnal club-AT2
Donor 1
C0
C1
C2
C3
C4
C5
C6
C7
C8
C9
C10
C11
C12
C13
C14
C15
C16
Donor 2
Additional file 6. “Integrated UMAP” and cell identity definition using the Azimuth analysis. A. (Left) The scRNA-seq data were processed and plotted into an "integrated UMAP" with 17 clusters (C0 to C16) as described in the additional file 2 and Methods. (Right) The UMAP is separately shown for each donor lung cell transcriptome at each timing (0 h in grey, 4 h in yellow, 10 h in red). B. the normalized expression values for each donor data sets before integration were analyzed using Azimuth algorithm and the Human lung v2 reference databse, to attribute an identity score to each cell. Only cells with scores > 0.6 were selected. The proportion of Azimuth identities in each of the 17 clusters is indicated on the heat map (100% = 1). For the next steps, only cells with identity representing > 10% of a cluster were considered (>0.1 on the heat map). The cell identity was defined by i) belonging to a cluster of the "integrated UMAP", ii) the Azimuth-based annotation (> score 0.6) and iii) a grouping of “close cell subtypes" in the cases of the B lymphocytes (B cells, plasma cells), stromal cells (smooth muscle, adventitial and alveolar fibroblasts) and blood endothelial cells (arterial, aerocytes, general capillary, venous systemic, venous pulmonary). From there, 23 cell identities were obtained. Ambiguous clusters were subsequently excluded (C6-AMs, C6-AT2s, C16-AMs, C16-Mast cells), therefore a total of 19 cell identities was selected for downstream analyses.
