# Supplementary material for: Cell type- and time-dependent biological responses in ex vivo perfused lung grafts
Source: Front Immunol. 2023 Jul 3;14:1142228. doi: 10.3389/fimmu.2023.1142228 (PMC10351384; doi:10.3389/fimmu.2023.1142228)
Supplement: Supplementary file 1 [file DataSheet_1.zip › Additional file-Data Sheet 1/Additional file 2-WorkFlow.pptx]

## Slide 1
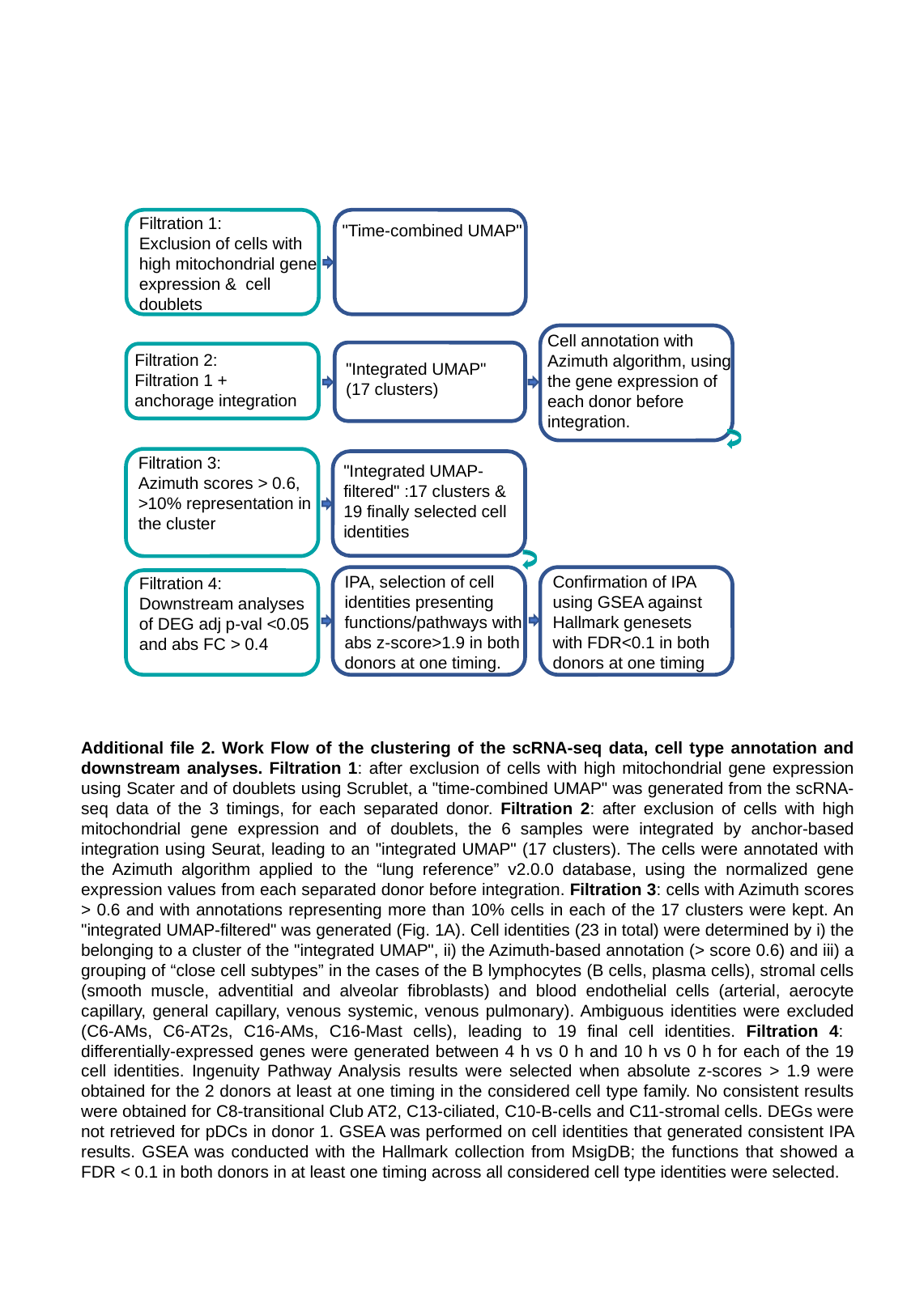

Filtration 1:
Exclusion of cells with high mitochondrial gene expression & cell doublets
"Time-combined UMAP"
Cell annotation with Azimuth algorithm, using the gene expression of each donor before integration.
Filtration 2:
Filtration 1 + anchorage integration
"Integrated UMAP" (17 clusters)
Filtration 3:
Azimuth scores > 0.6, >10% representation in the cluster
"Integrated UMAP-filtered" :17 clusters & 19 finally selected cell identities
IPA, selection of cell identities presenting functions/pathways with abs z-score>1.9 in both donors at one timing.
Confirmation of IPA using GSEA against Hallmark genesets with FDR<0.1 in both donors at one timing
Filtration 4:
Downstream analyses of DEG adj p-val <0.05 and abs FC > 0.4
Additional file 2. Work Flow of the clustering of the scRNA-seq data, cell type annotation and downstream analyses. Filtration 1: after exclusion of cells with high mitochondrial gene expression using Scater and of doublets using Scrublet, a "time-combined UMAP" was generated from the scRNA-seq data of the 3 timings, for each separated donor. Filtration 2: after exclusion of cells with high mitochondrial gene expression and of doublets, the 6 samples were integrated by anchor-based integration using Seurat, leading to an "integrated UMAP" (17 clusters). The cells were annotated with the Azimuth algorithm applied to the “lung reference” v2.0.0 database, using the normalized gene expression values from each separated donor before integration. Filtration 3: cells with Azimuth scores > 0.6 and with annotations representing more than 10% cells in each of the 17 clusters were kept. An "integrated UMAP-filtered" was generated (Fig. 1A). Cell identities (23 in total) were determined by i) the belonging to a cluster of the "integrated UMAP", ii) the Azimuth-based annotation (> score 0.6) and iii) a grouping of “close cell subtypes” in the cases of the B lymphocytes (B cells, plasma cells), stromal cells (smooth muscle, adventitial and alveolar fibroblasts) and blood endothelial cells (arterial, aerocyte capillary, general capillary, venous systemic, venous pulmonary). Ambiguous identities were excluded (C6-AMs, C6-AT2s, C16-AMs, C16-Mast cells), leading to 19 final cell identities. Filtration 4: differentially-expressed genes were generated between 4 h vs 0 h and 10 h vs 0 h for each of the 19 cell identities. Ingenuity Pathway Analysis results were selected when absolute z-scores > 1.9 were obtained for the 2 donors at least at one timing in the considered cell type family. No consistent results were obtained for C8-transitional Club AT2, C13-ciliated, C10-B-cells and C11-stromal cells. DEGs were not retrieved for pDCs in donor 1. GSEA was performed on cell identities that generated consistent IPA results. GSEA was conducted with the Hallmark collection from MsigDB; the functions that showed a FDR < 0.1 in both donors in at least one timing across all considered cell type identities were selected.
