# Supplementary material for: Cell type- and time-dependent biological responses in ex vivo perfused lung grafts
Source: Front Immunol. 2023 Jul 3;14:1142228. doi: 10.3389/fimmu.2023.1142228 (PMC10351384; doi:10.3389/fimmu.2023.1142228)

**Additional file 14. Heat map of the expression modulation of the genes contributing to selected IPA pathways and functions across the cell subtypes of the lymphoid cell family**. For pathways and functions of the IPA results mentioned in the main body text, a list of contributing genes was established from the union of the cases with absolute z-scores > 1.9. The gene expression fold changes (log2) of the contributing gene list is illustrated as a heat map, based on the shown scale. The pathways/functions illustrated are: Activation of Lymphocytes, Cytotoxicity of Lymphocytes, CTLA4 Signaling of cytotoxic T Lymphocytes. No z-score > 1.9 was obtained for Apoptosis of T lymphocytes. Arrows point to the genes mentioned in the main text.

Lymphoid cells – CD4^+^ T cells, CD8^+^ T cells and NK cells– Activation of Lymphocytes


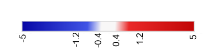

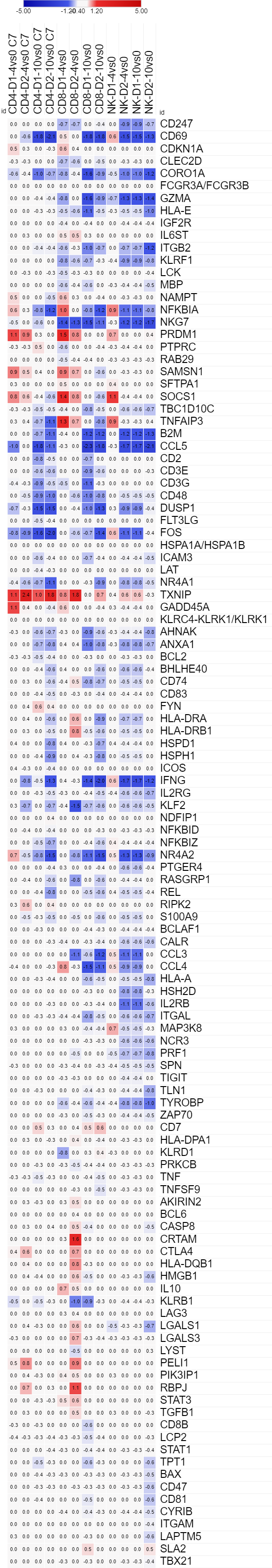

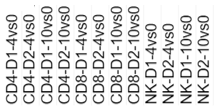

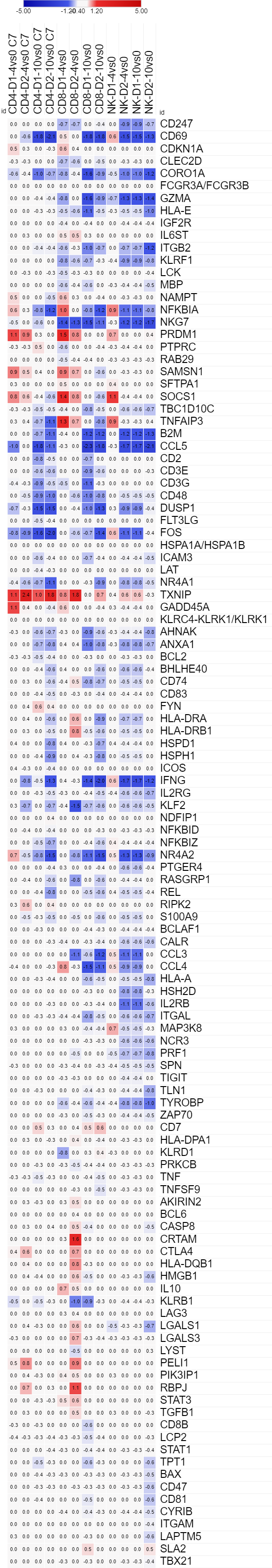

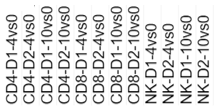


Lymphoid cells – CD4^+^ T cells, CD8^+^ T cells and NK cells– Cytotoxicity of Lymphocytes


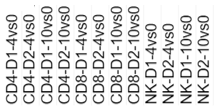


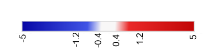


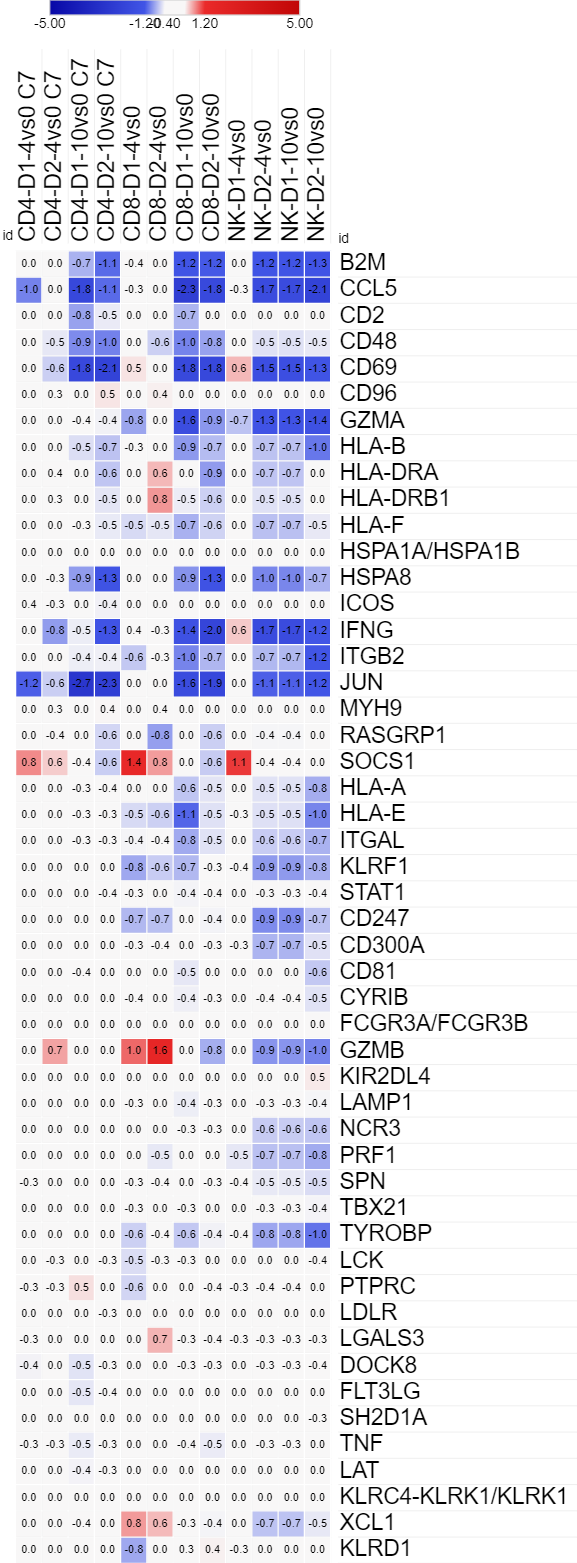


Lymphoid cells – CD4^+^ T cells, CD8^+^ T cells and NK cells– CTLA4 signaling in cytotoxic T lymphocytes


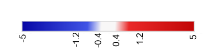

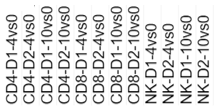

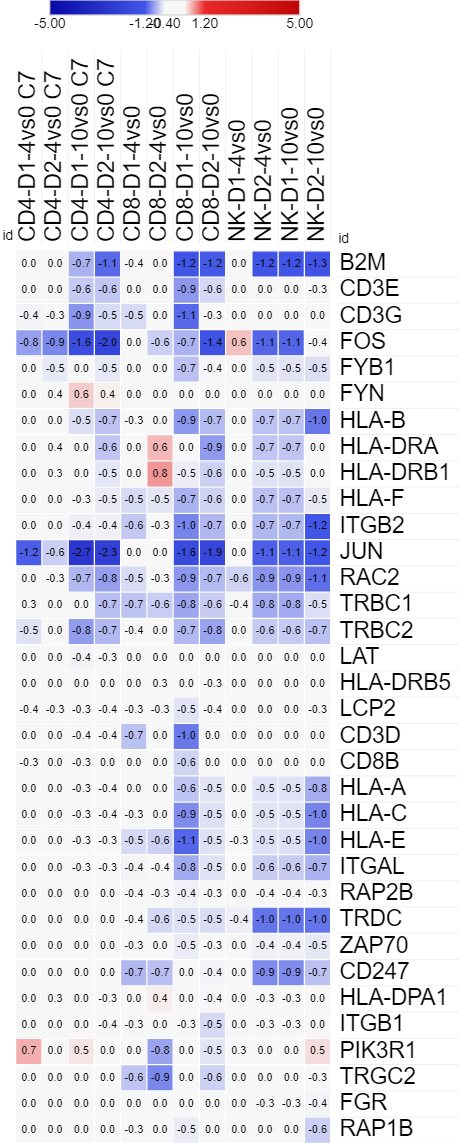

Supplement: Supplementary file 1 [file DataSheet_1.zip › Additional file-Data Sheet 1/Additional file 14-Contributing genes Ly-NK.docx]
