# Supplementary material for: Cell type- and time-dependent biological responses in ex vivo perfused lung grafts
Source: Front Immunol. 2023 Jul 3;14:1142228. doi: 10.3389/fimmu.2023.1142228 (PMC10351384; doi:10.3389/fimmu.2023.1142228)
Supplement: Supplementary file 1 [file DataSheet_1.zip › Additional file-Data Sheet 1/Additional file 1-donors.docx]

**Additional file 1. Donor characteristics and oxygenation function of their lungs**

**A. Donor characteristics**

|  | Donor 1 |  | Donor 2 |
| --- | --- | --- | --- |
| Cold ischemia | 2 h |  | 6 h |
| Warm ischemia | none |  | none |
| Age | 56 yr |  | 84 yr |
| Sex | Male |  | Female |
| Tobacco | Yes (low, 15 packs/yr) |  | No |
| Disease | High blood pressure |  | High blood pressure |
| Cause of death | Drug induced- hyperkalemia and brain death |  | Stroke |
| Intensive care | 5 days |  | 1 day |
| Reason for  decline | Blood AB+ (no recipient) |  | Age |

**B. Oxygenation function**

**Oxygenation function during EVLP for donor 1 and 2**. The PO2 in the left atrial (reported to 100% oxygen) was monitored during the 10 h EVLP (red for donor 1, black for donor 2).
