# Supplementary material for: Cell type- and time-dependent biological responses in ex vivo perfused lung grafts
Source: Front Immunol. 2023 Jul 3;14:1142228. doi: 10.3389/fimmu.2023.1142228 (PMC10351384; doi:10.3389/fimmu.2023.1142228)

**Additional file 13. Heat map of the expression modulation of the genes contributing to selected IPA pathways and functions across the cell subtypes of the endothelial cell family**. For pathways and functions of the IPA results mentioned in the main body text, a list of contributing genes was established from the union of the cases with absolute z-scores > 1.9. The gene expression fold changes (log2) of the contributing gene list is illustrated as a heat map, based on the shown scale. The pathways/functions illustrated are: Inflammatory responses, Migration of Phagocytes, Synthesis of eicosanoids, Apoptosis, IL-17 Signaling, Pathogen-induced Cytokine Storm Signaling, Endothelin-1 Signaling, Apelin Endothelial Signaling. Arrows point to genes mentioned in the main text.

Endothelial cells – Blood endothelial cells and lymphatic endothelial cells - Inflammatory response


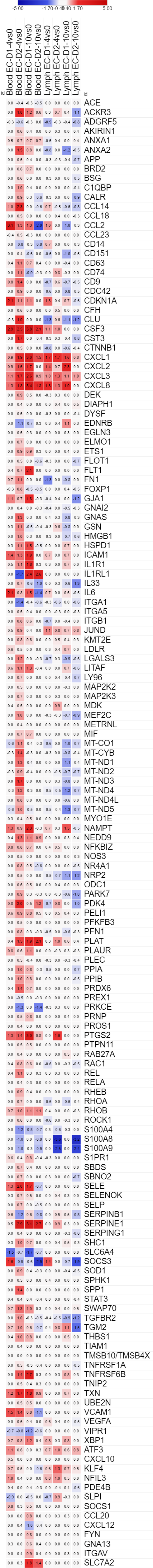

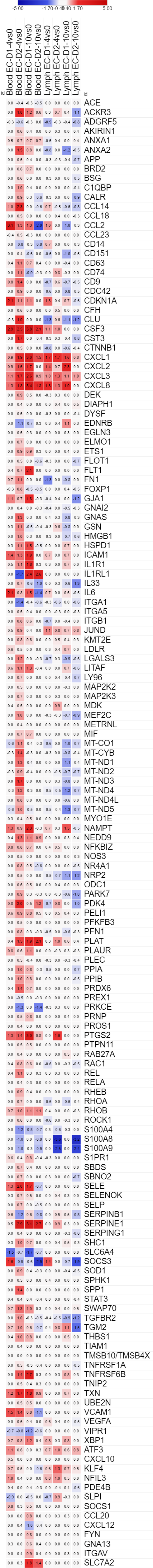


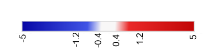


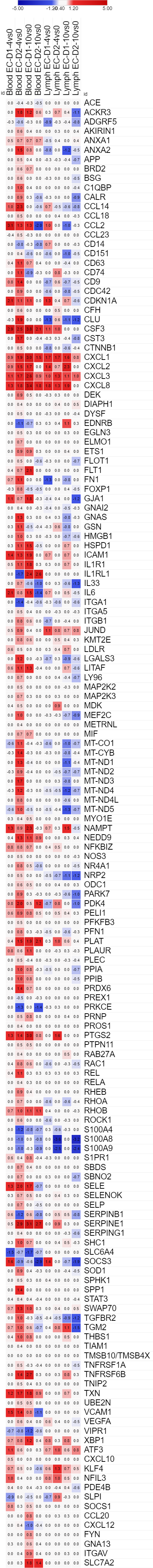

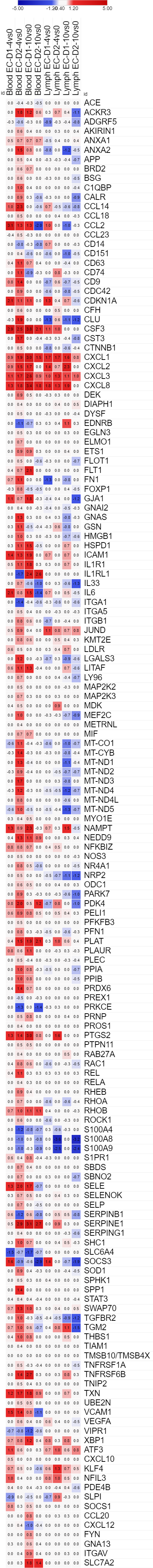


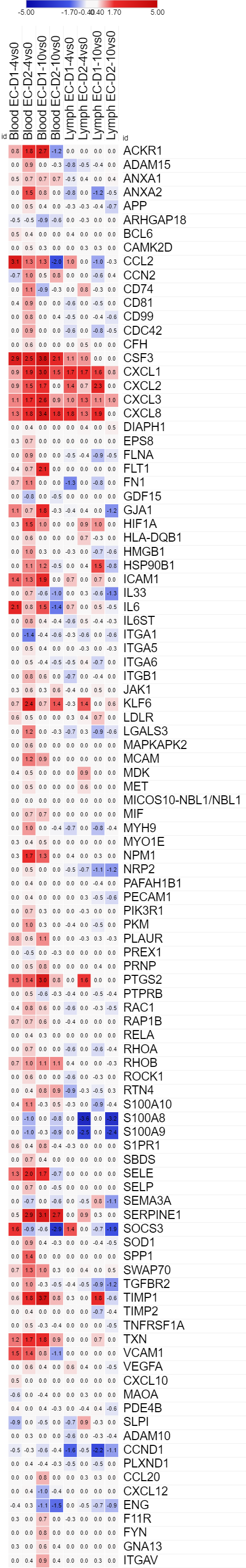
Endothelial cells – Blood endothelial cells and lymphatic endothelial cells – Migration of phagocytes


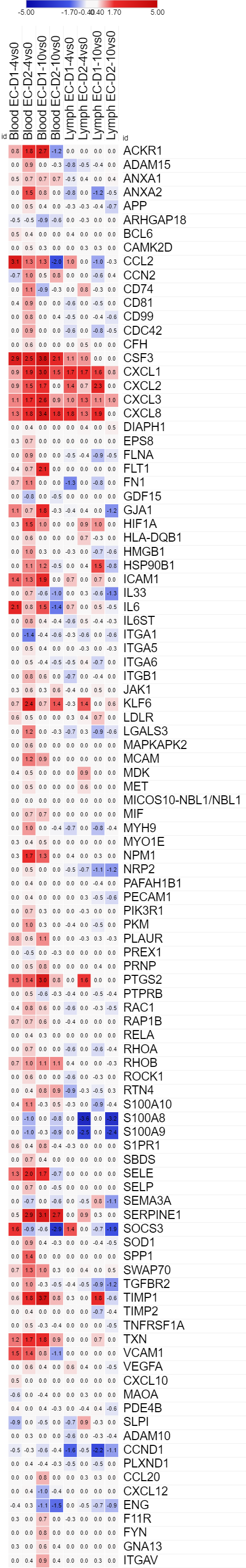


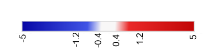


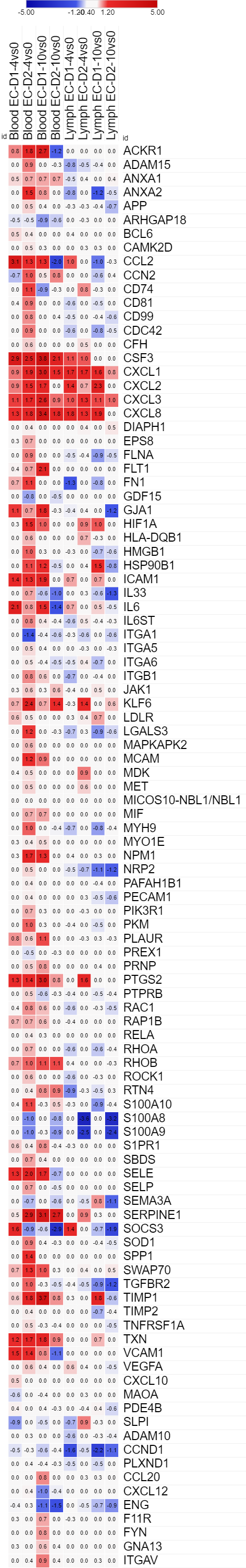

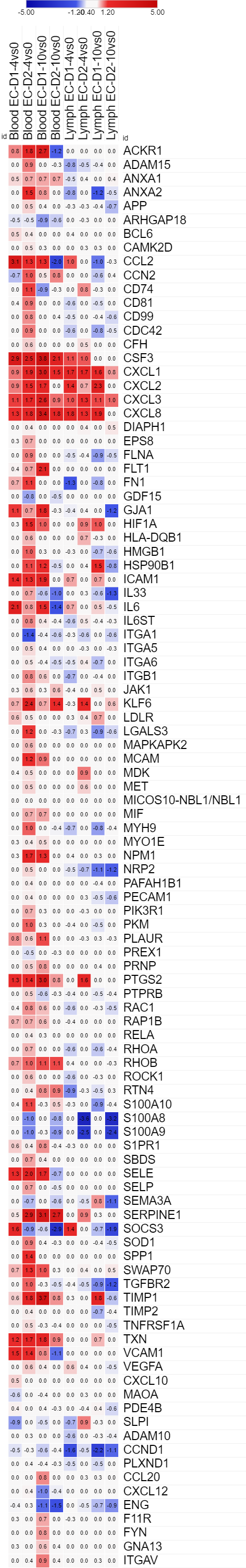


Endothelial cells – Blood endothelial cells and lymphatic endothelial cells –Synthesis of eicosanoid


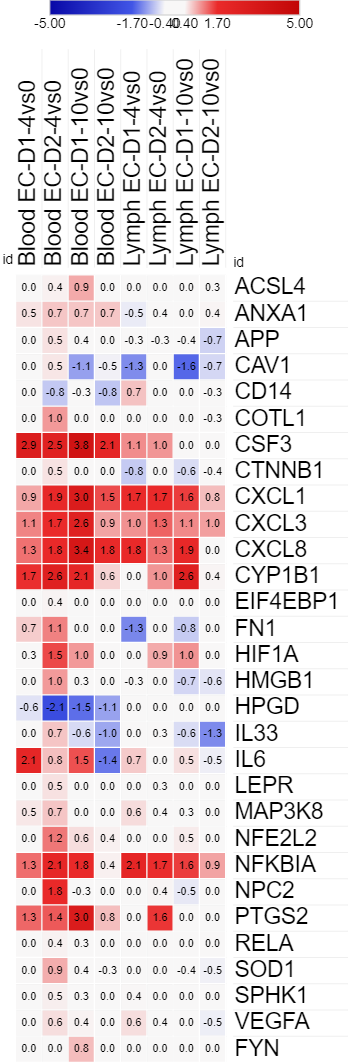


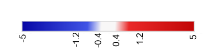


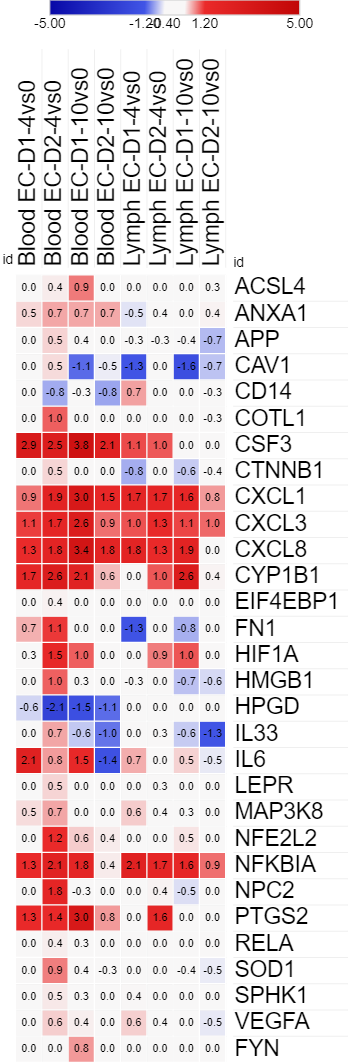


Endothelial cells – Blood endothelial cells and lymphatic endothelial cells - Apoptosis


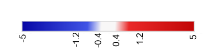

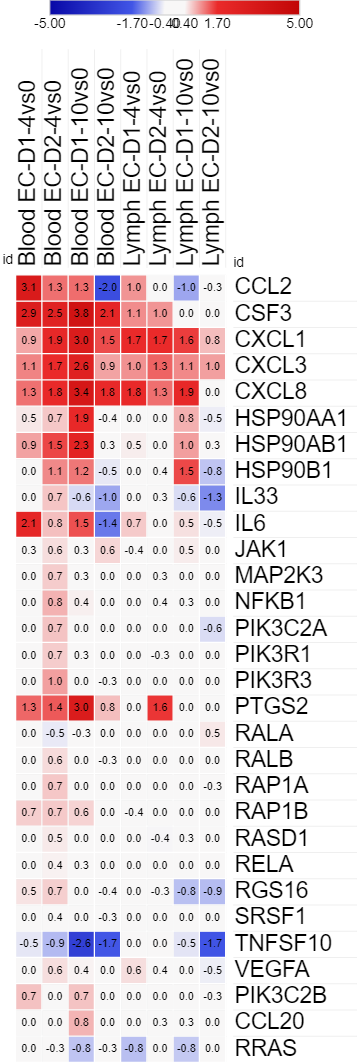

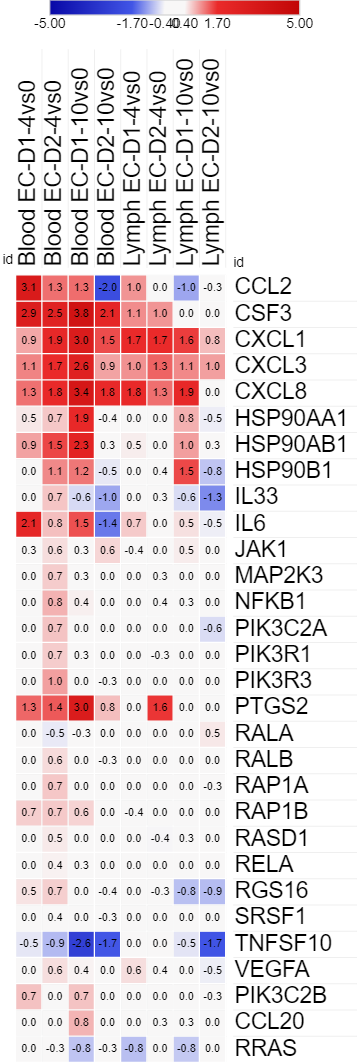

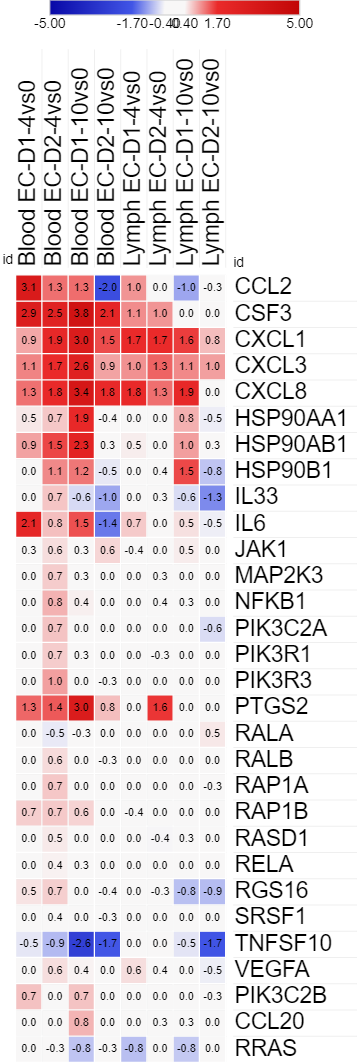

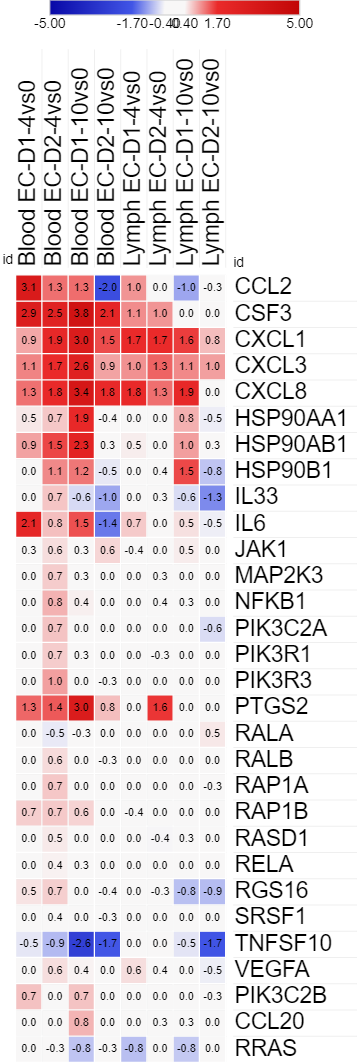

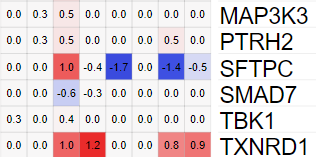

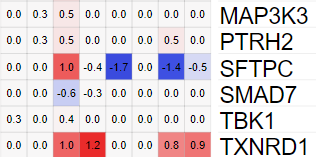

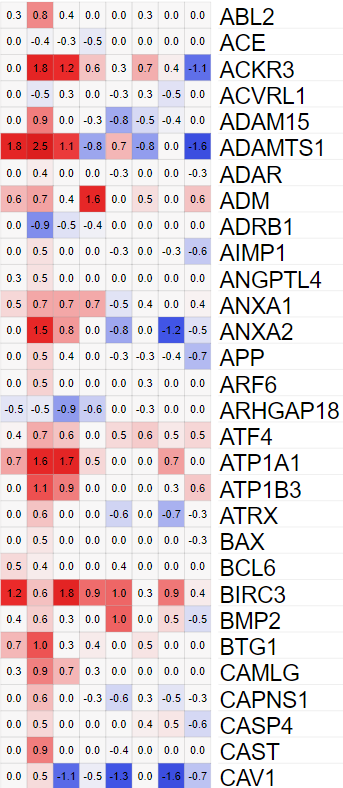

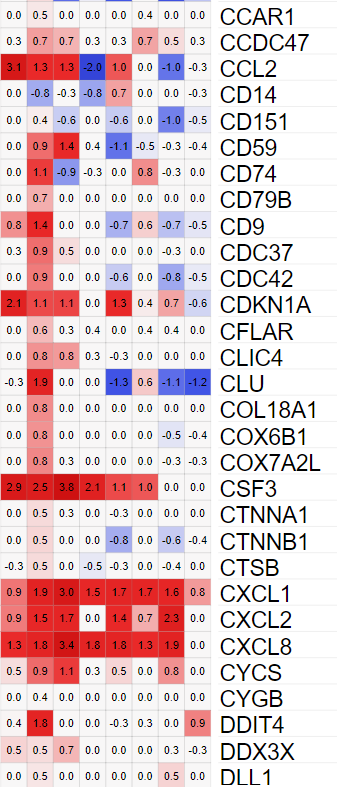

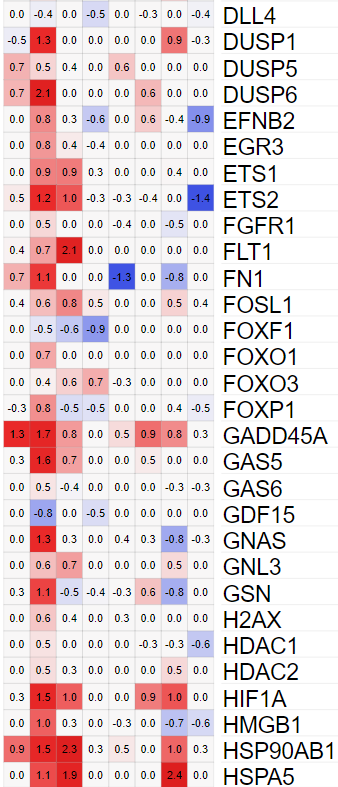

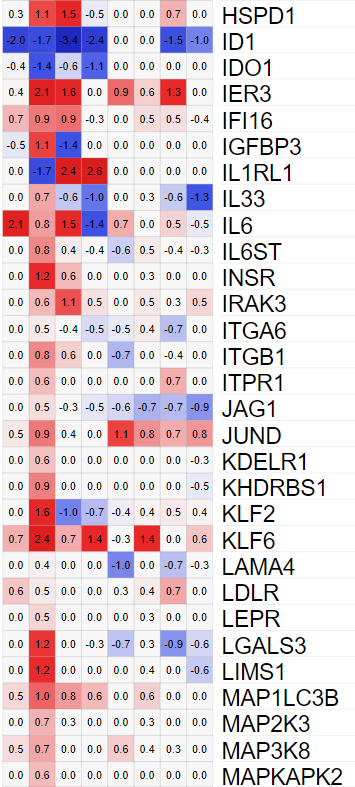

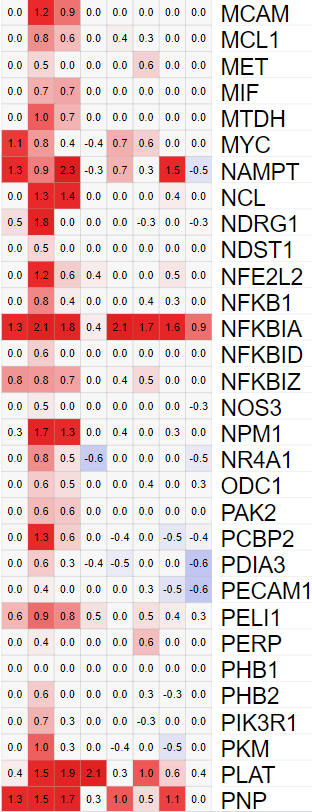

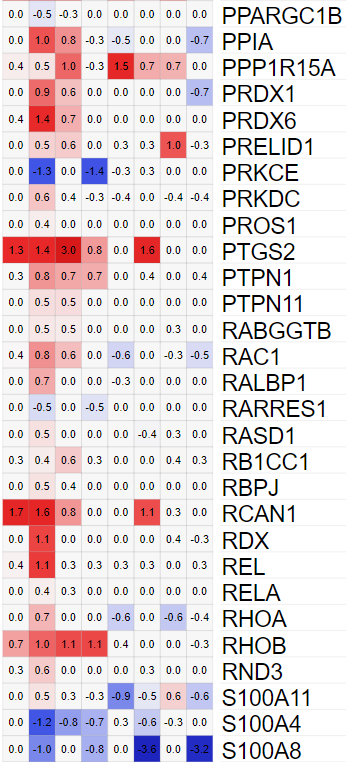

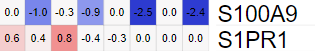

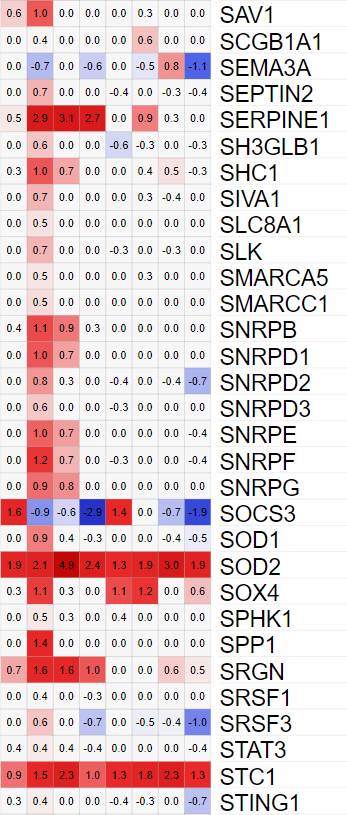

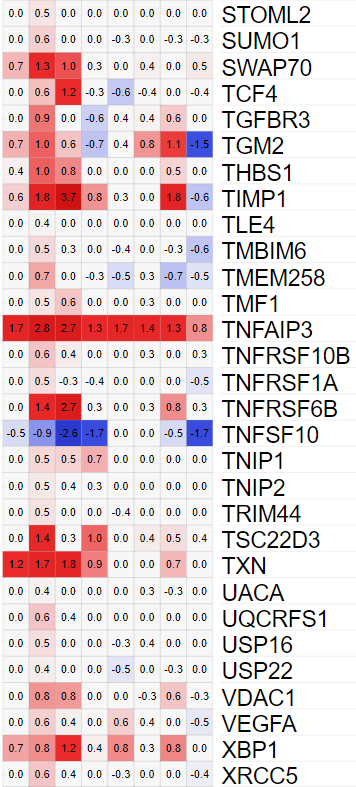

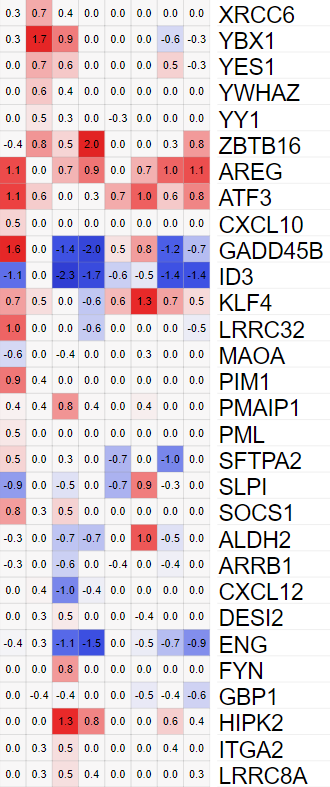


Endothelial cells – Blood endothelial cells and lymphatic endothelial cells – IL-17 Signaling


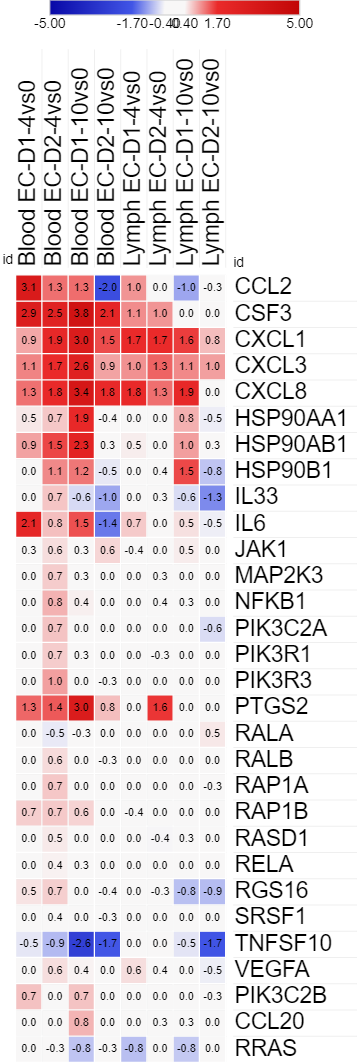


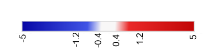


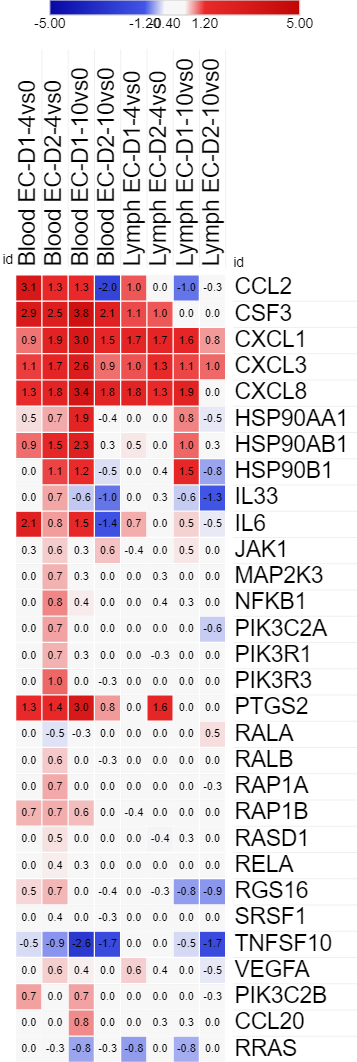


Endothelial cells – Blood endothelial cells and lymphatic endothelial cells – Pathogen Induced Cytokine Storm Signaling Pathway


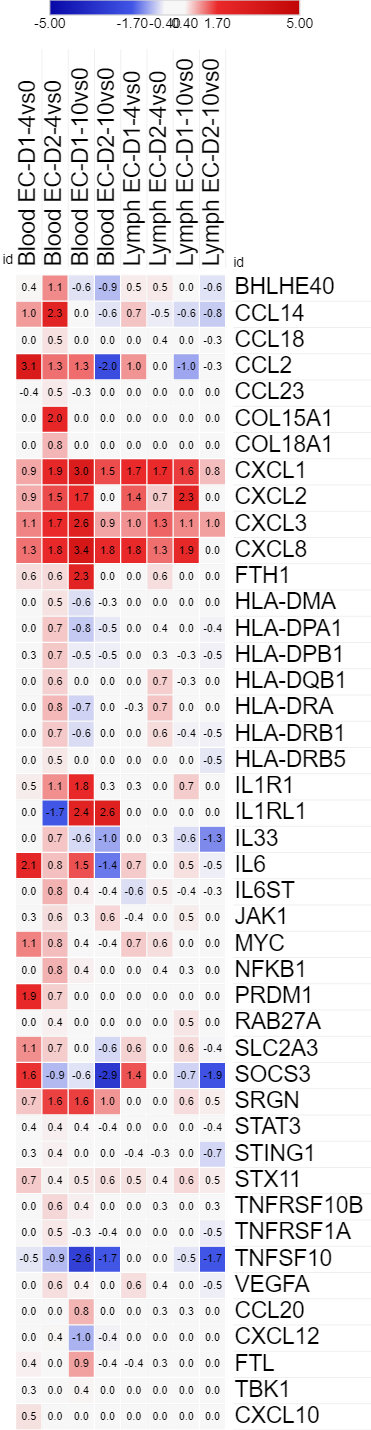


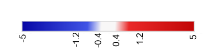


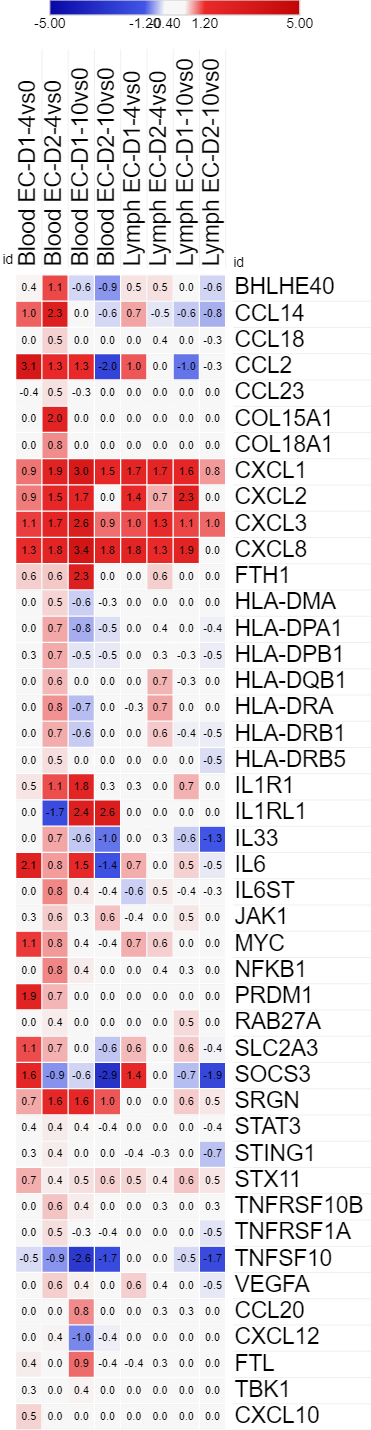


Endothelial cells – Blood endothelial cells and lymphatic endothelial cells – Endothelin-1 Signaling


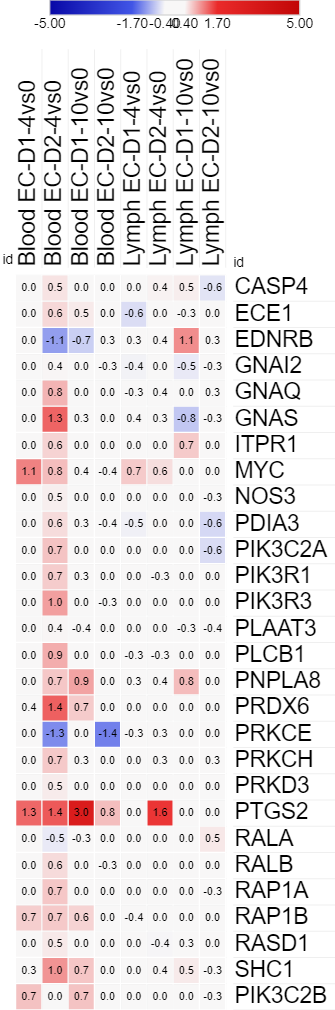


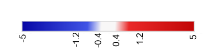


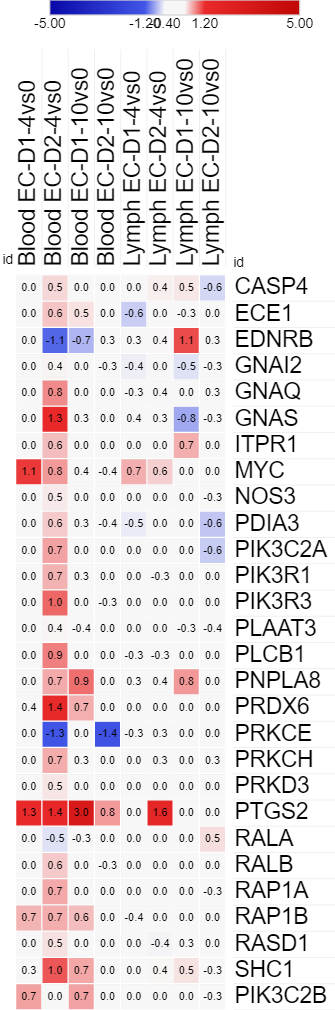


Endothelial cells – Blood endothelial cells and lymphatic endothelial cells – Apelin endothelial signaling pathway


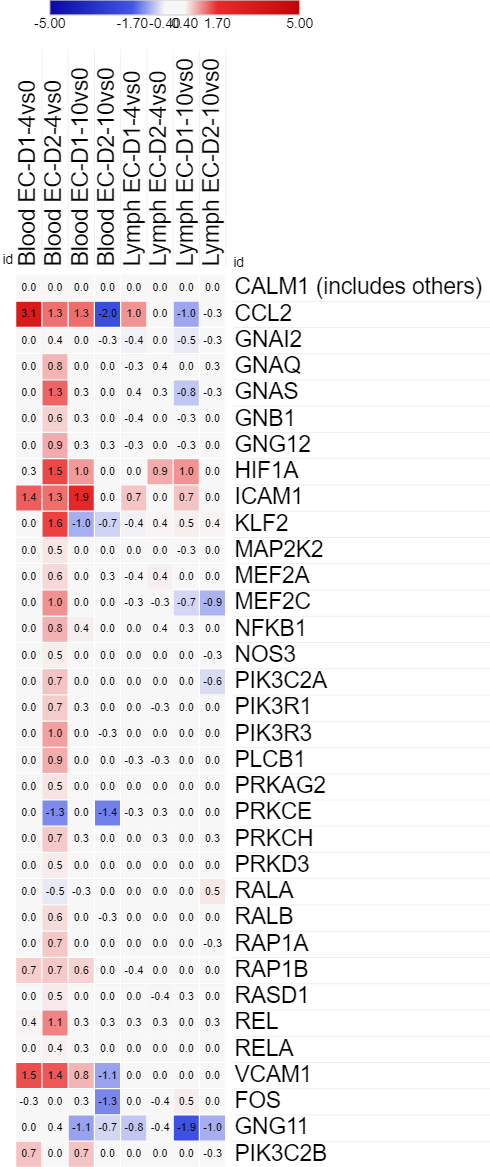


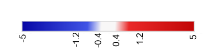

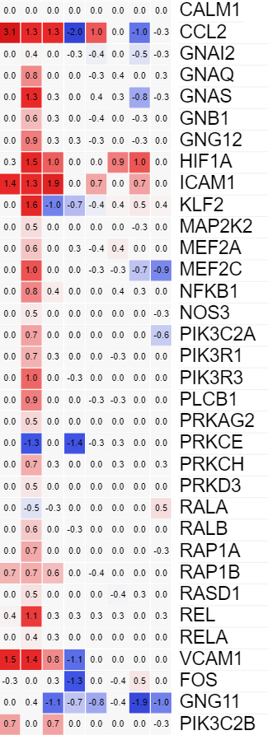

Supplement: Supplementary file 1 [file DataSheet_1.zip › Additional file-Data Sheet 1/Additional file 13-Contributing genes EC.docx]
