# Supplementary material for: Cell type- and time-dependent biological responses in ex vivo perfused lung grafts
Source: Front Immunol. 2023 Jul 3;14:1142228. doi: 10.3389/fimmu.2023.1142228 (PMC10351384; doi:10.3389/fimmu.2023.1142228)

**Additional file 11. Heat maps of the expression modulation of the genes contributing to selected IPA pathways and functions across the cell subtypes of the epithelial cell family**. For pathways and functions of the IPA results mentioned in the main body text, a list of contributing genes was established from the union of the cases with absolute z-scores > 1.9. The gene expression fold changes (log2) of the contributing gene list is illustrated as a heat map, based on the shown scale. The pathways/functions illustrated are: Migration of phagocytes, Apoptosis, Th17 Activation Pathways, Pathogen-Induced Cytokine Storm Signaling, Actin Cytoskeleton Signaling, GNRH Signaling. Arrows point to genes mentioned in the main text.

Epithelial cells – Alveolar type 2/Alveolar type 1 – Migration of phagocytes


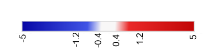

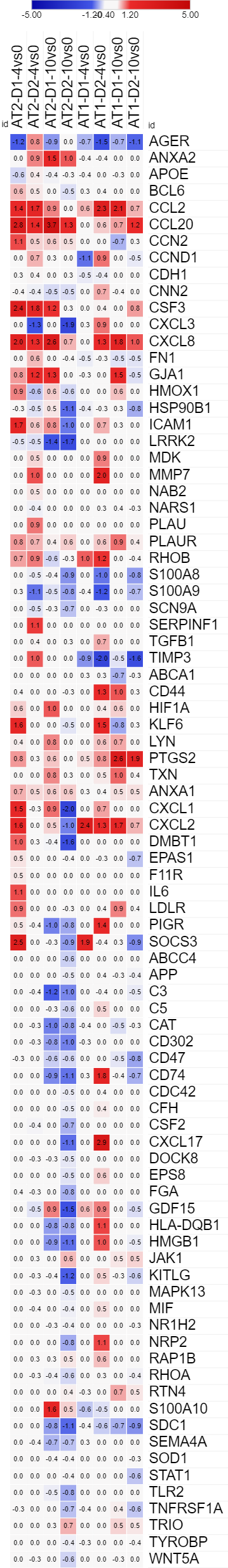


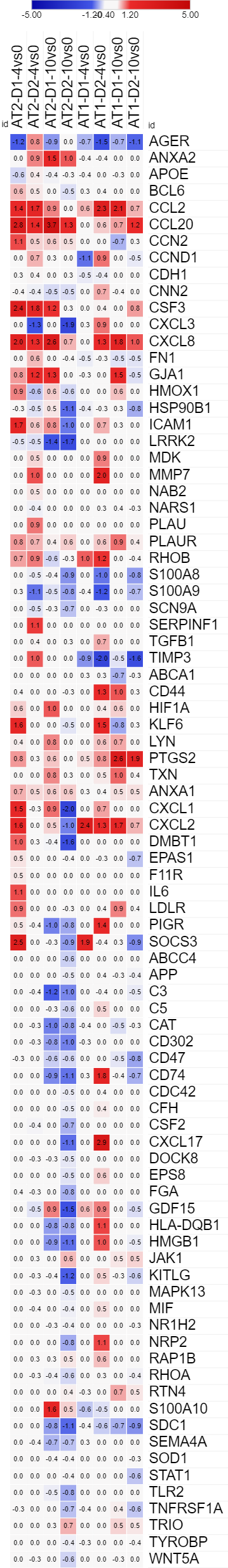


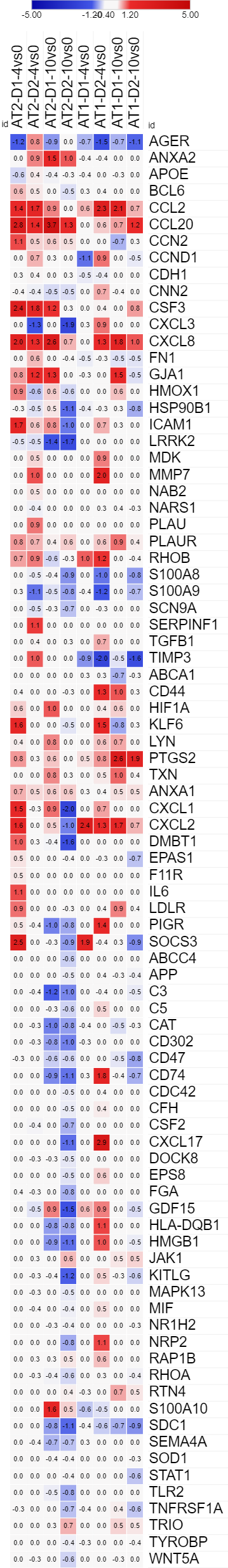

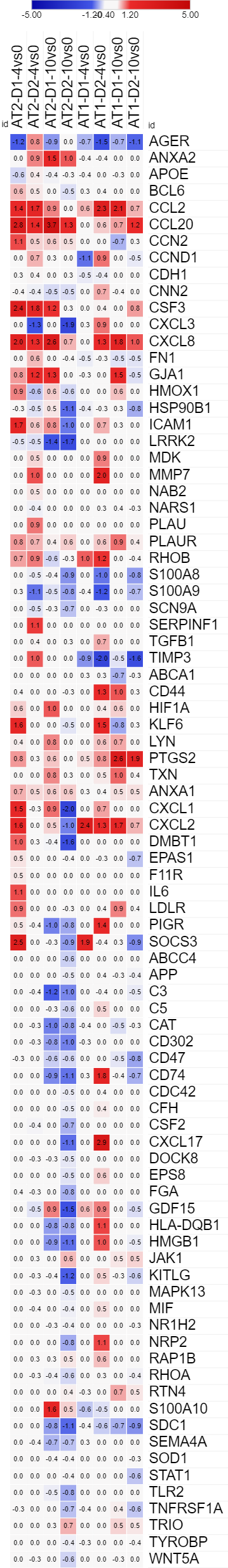
Epithelial cells – Alveolar type 2/Alveolar type 1 – Apoptosis


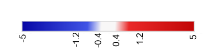


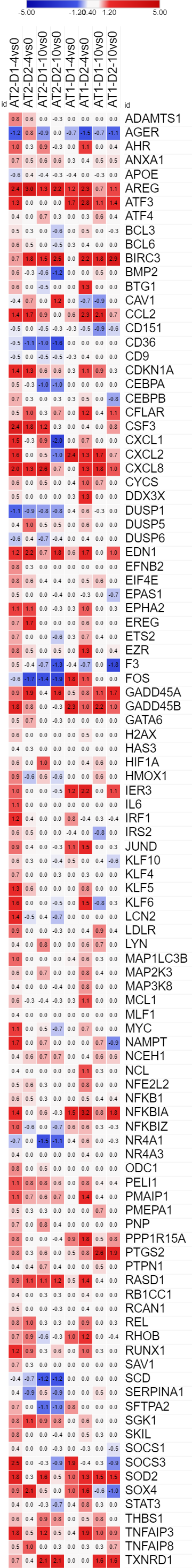

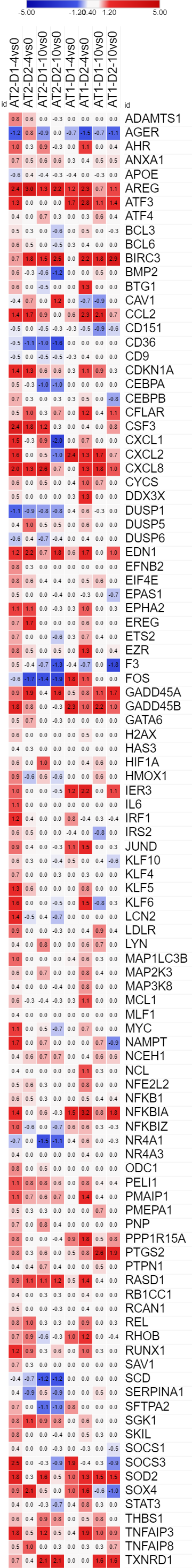


Epithelial cells – Alveolar type 2/Alveolar type 1 – Th17 activation pathway


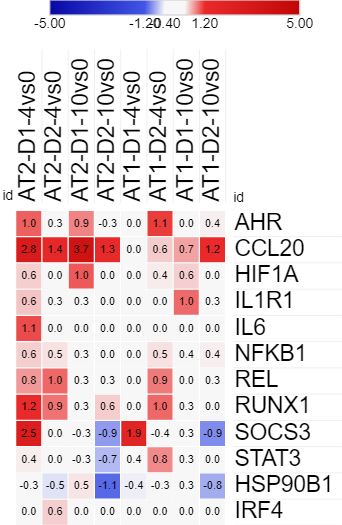


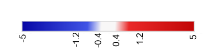


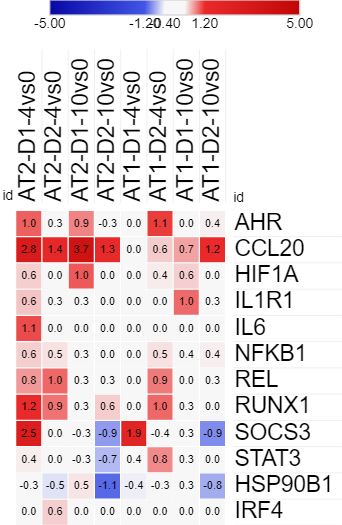


Epithelial cells – Alveolar type 2/Alveolar type 1 – Pathogen induced cytokine storm signaling pathway


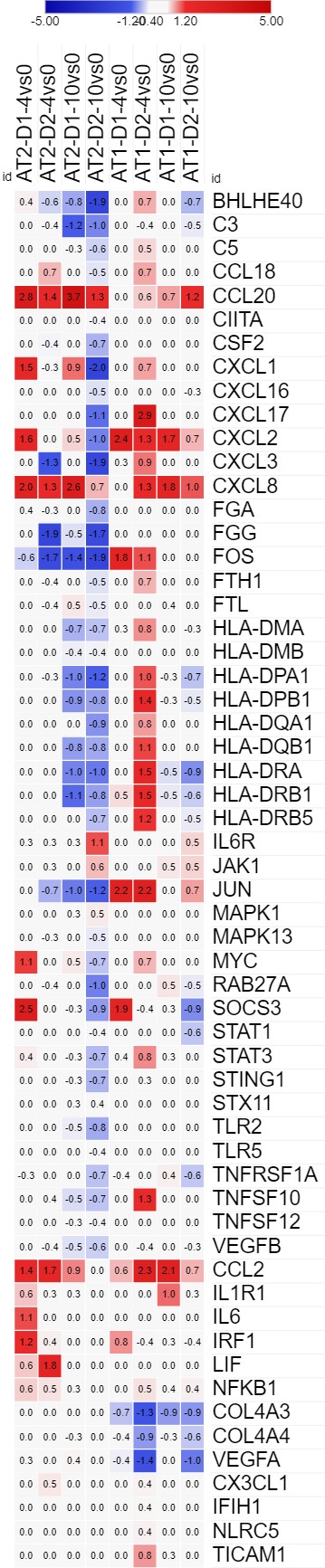


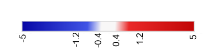


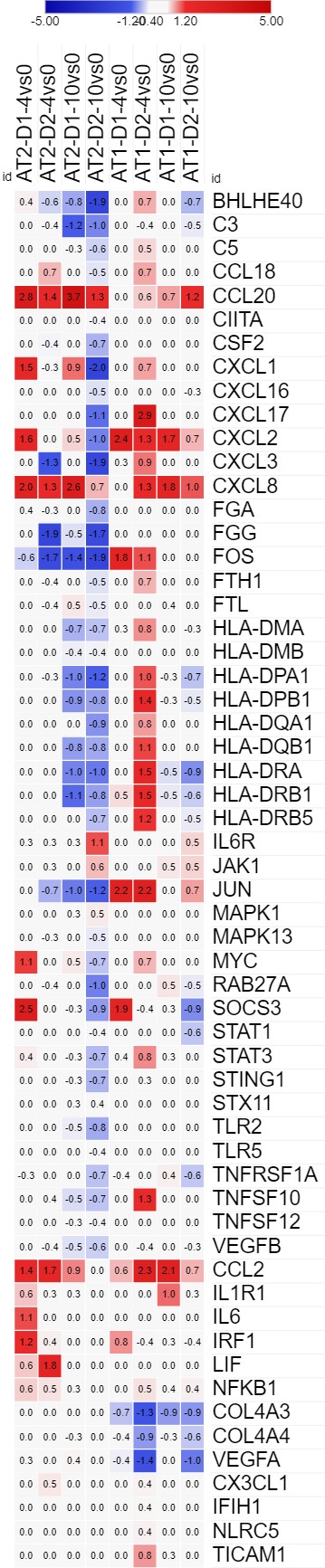


Epithelial cells – Alveolar type 2/Alveolar type 1 – Actin cytoskeleton signaling


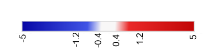

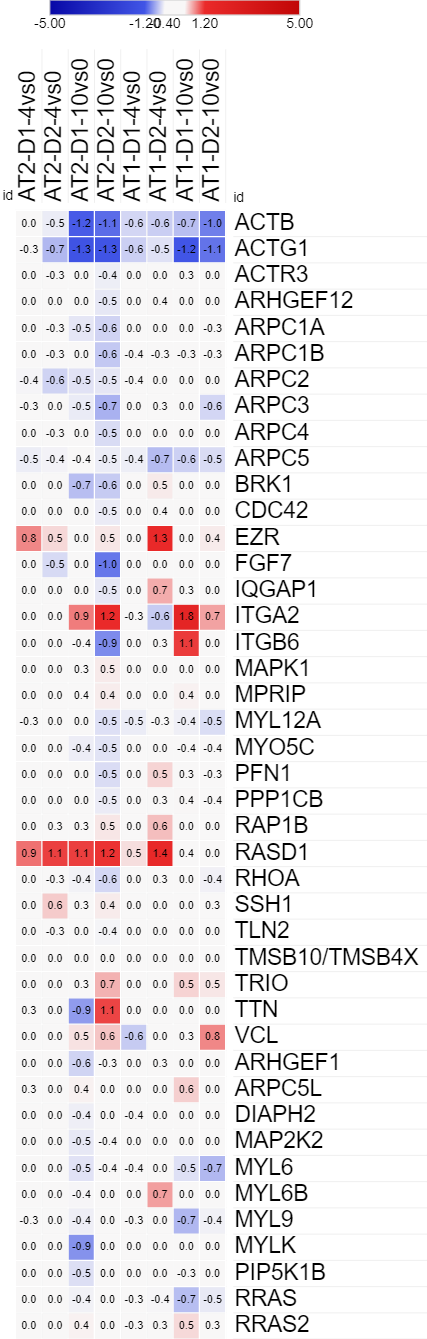


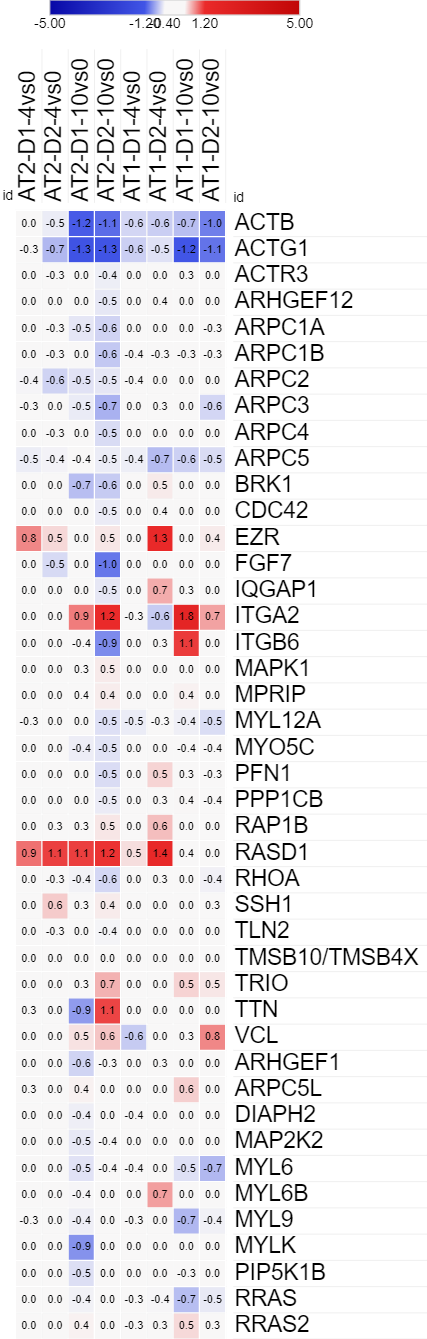


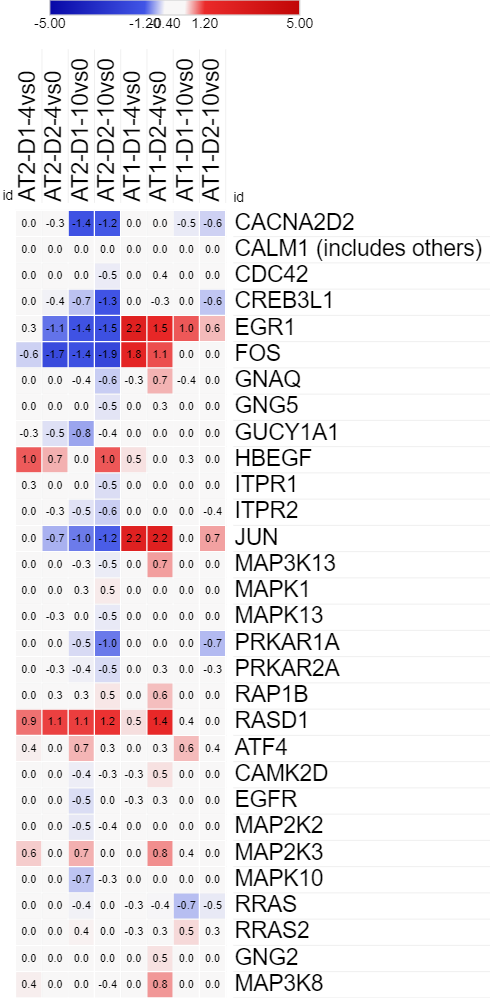
Epithelial cells – Alveolar type 2/Alveolar type 1 – GNRH signaling


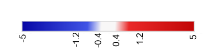

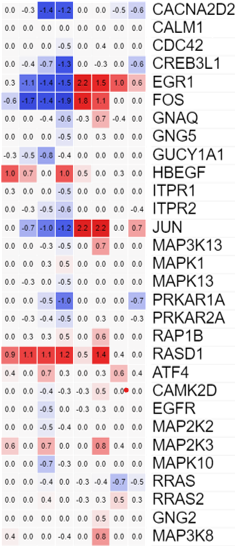

Supplement: Supplementary file 1 [file DataSheet_1.zip › Additional file-Data Sheet 1/Additional file 11-Contributing genes AT1-AT2.docx]
