# Supplementary material for: Cell type- and time-dependent biological responses in ex vivo perfused lung grafts
Source: Front Immunol. 2023 Jul 3;14:1142228. doi: 10.3389/fimmu.2023.1142228 (PMC10351384; doi:10.3389/fimmu.2023.1142228)
Supplement: Supplementary file 1 [file DataSheet_1.zip › Additional file-Data Sheet 1/Additional file 5-Time-combined UMAPs.pptx]

## Slide 1
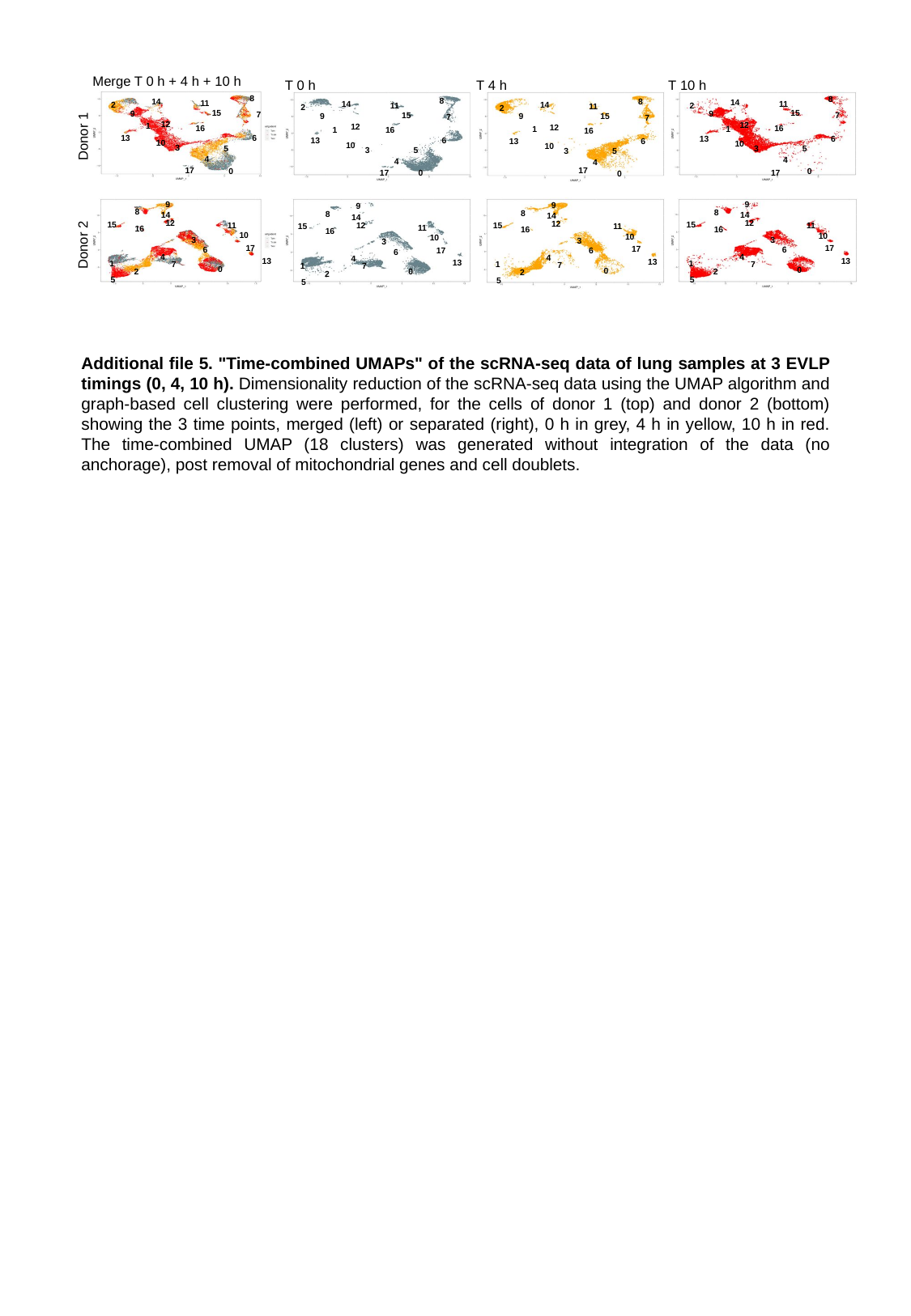

Merge T 0 h + 4 h + 10 h
T 0 h
T 4 h
T 10 h
8
14
11
2
15
9
7
12
16
6
13
10
3
5
4
0
8
14
11
2
15
9
7
12
16
6
13
10
3
5
4
0
8
14
11
2
15
9
7
12
16
6
13
10
3
5
4
0
8
14
11
2
15
9
7
12
16
6
13
10
3
5
4
0
18
1
1
1
1
1
Donor 1
17
17
17
17
17
9
8
14
12
15
11
16
10
3
17
6
4
13
1
7
0
2
5
9
8
14
12
15
11
16
10
3
17
6
4
13
1
7
0
2
5
9
8
14
12
15
11
16
10
3
17
6
4
13
1
7
0
2
5
9
8
14
12
15
11
16
10
3
17
6
4
13
1
7
0
2
5
Donor 2
Additional file 5. "Time-combined UMAPs" of the scRNA-seq data of lung samples at 3 EVLP timings (0, 4, 10 h). Dimensionality reduction of the scRNA-seq data using the UMAP algorithm and graph-based cell clustering were performed, for the cells of donor 1 (top) and donor 2 (bottom) showing the 3 time points, merged (left) or separated (right), 0 h in grey, 4 h in yellow, 10 h in red. The time-combined UMAP (18 clusters) was generated without integration of the data (no anchorage), post removal of mitochondrial genes and cell doublets.
